# Chemical Modulation of the 1-(Piperidin-4-yl)-1,3-dihydro-2*H*-benzo[d]imidazole-2-one Scaffold as a Novel NLRP3 Inhibitor

**DOI:** 10.3390/molecules26133975

**Published:** 2021-06-29

**Authors:** Simone Gastaldi, Valentina Boscaro, Eleonora Gianquinto, Christina F. Sandall, Marta Giorgis, Elisabetta Marini, Federica Blua, Margherita Gallicchio, Francesca Spyrakis, Justin A. MacDonald, Massimo Bertinaria

**Affiliations:** 1 Department of Drug Science and Technology, University of Turin, Via Giuria 9, 10125 Torino, Italy; simone.gastaldi@unito.it (S.G.); valentina.boscaro@unito.it (V.B.); eleonora.gianquinto@unito.it (E.G.); marta.giorgis@unito.it (M.G.); elisabetta.marini@unito.it (E.M.); federica.blua@unito.it (F.B.); margherita.gallicchio@unito.it (M.G.); francesca.spyrakis@unito.it (F.S.); 2 Department of Biochemistry & Molecular Biology, Cumming School of Medicine, University of Calgary, 3280 Hospital Drive NW, Calgary, AB T2N 4Z6, Canada; cfsandal@ucalgary.ca (C.F.S.); jmacdo@ucalgary.ca (J.A.M.)

**Keywords:** NLRP3 inhibitors, pyroptosis, interleukin-1β, ATP hydrolysis, MD simulations

## Abstract

In the search for new chemical scaffolds able to afford NLRP3 inflammasome inhibitors, we used a pharmacophore-hybridization strategy by combining the structure of the acrylic acid derivative INF39 with the 1-(piperidin-4-yl)1,3-dihydro-2*H*-benzo[d]imidazole-2-one substructure present in HS203873, a recently identified NLRP3 binder. A series of differently modulated benzo[d]imidazole-2-one derivatives were designed and synthesised. The obtained compounds were screened in vitro to test their ability to inhibit NLRP3-dependent pyroptosis and IL-1β release in PMA-differentiated THP-1 cells stimulated with LPS/ATP. The selected compounds were evaluated for their ability to reduce the ATPase activity of human recombinant NLRP3 using a newly developed assay. From this screening, compounds **9**, **13** and **18**, able to concentration-dependently inhibit IL-1β release in LPS/ATP-stimulated human macrophages, emerged as the most promising NLRP3 inhibitors of the series. Computational simulations were applied for building the first complete model of the NLRP3 inactive state and for identifying possible binding sites available to the tested compounds. The analyses led us to suggest a mechanism of protein–ligand binding that might explain the activity of the compounds.

## 1. Introduction

The nucleotide-binding oligomerization domain leucine rich repeat and pyrin domain containing protein 3 (NLRP3) inflammasome is a cytosolic pattern recognition receptor (PRR) that plays a fundamental role in the response to exogenous and endogenous stimuli. The NLRP3 inflammasome belongs to the NOD-like receptor (NLR) family of PRR; it is a multiprotein complex constituted by the NLRP3 protein, the apoptosis-associated speck-like protein containing a caspase-recruiting domain (ASC) and procaspase-1 [1,2,3]. The NLRP3 protein can be seen as the central core of the NLRP3 inflammasome. It is constituted by three domains: the C-terminal leucine-rich repeat domain (LRR), the nucleotide-binding and oligomerization domain (NACHT) and the N-terminal pyrin domain (PYD). Each of these domains play a different role. The role of the LRR domain is still to be fully clarified: LRR appears to be dispensable for NLRP3 activation [4], however, by binding to NIMA-related kinase 7 (NEK7), it facilitates the ATP-dependent activation of NLRP3 [2,5]; the NACHT domain is mainly responsible for oligomerization and possesses ATP-binding and hydrolysis abilities [6]; finally, the PYD domain interacts with ASC through homotypic (PYD–PYD) interactions [7]. NLRP3 expression is induced following the stimulation by danger-associated molecular patterns (DAMPs) or pathogen-associated molecular patterns (PAMPs) of Toll-like receptors (TLRs), such as TLR4 receptors, or cytokine receptors, such as TNF receptor. This process, known as the *priming step*, is not mandatory in any cell types [8]. NLRP3 is mainly expressed in monocytes, macrophages, lymphocytes, neutrophils and dendritic cells; however, it is also expressed in microglia, astrocytes and epithelial cells [9,10]. NLRP3 usually needs a second signal, generally referred to as an *activation signal*, to be fully activated. Different signals can lead to inflammasome activation [7,11,12,13]. Nigericin, ATP, molecular particulates and crystals induce, through different mechanisms, a K^+^ efflux that is considered a common trigger for NLRP3 activation [14]. However, altered Ca^2+^ homeostasis and other ion fluxes (e.g., Na^+^ and Cl^−^) have also been implicated in NLRP3 activation [15,16]. Several studies demonstrated that reactive oxygen species (ROS) production, lysosomal destabilization and post-translational modifications are other cellular triggers able to promote the activation and assembly of NLRP3 inflammasome [17,18,19,20,21]. Once activated, NLRP3 undergoes a conformational change and then oligomerizes to give rise to a functional inflammasome, which can trigger the auto-proteolytic cleavage of pro-caspase-1 into the active caspase-1 [22]. Finally, this cysteine protease converts the pro-inflammatory cytokines pro-interleukin (IL)-1β and pro-IL-18 into their active counterparts IL-1β and IL-18. The activated caspase-1 is also able to cleave the protein gasdermin-D (GSDMD). The cytotoxic GSDMD-N terminal fragments can assemble in large circular structures that, by localizing into the cellular membrane, cause the formation of pores that leads to the membrane rupture and the pyroptotic cell death [23]. This process, known as pyroptosis, contributes to the release of IL-1β and other intracellular material into the extracellular space, thus exacerbating the inflammatory process.

The aberrant activation of NLRP3 inflammasome is involved in the onset and progression of a wide range of human diseases [24], among which auto-inflammatory [25], inflammatory and autoimmune [26,27,28,29], neurodegenerative [30,31], cardiovascular [32,33,34] and metabolic diseases [35,36,37] are the most studied. It is not surprising that a concerted research effort toward the discovery of small molecules able to block NLRP3 activation is being performed both by industry and academia. These efforts have enabled the discovery of interesting compounds, some of which are now in clinical development [38,39,40].

**Figure 1:**
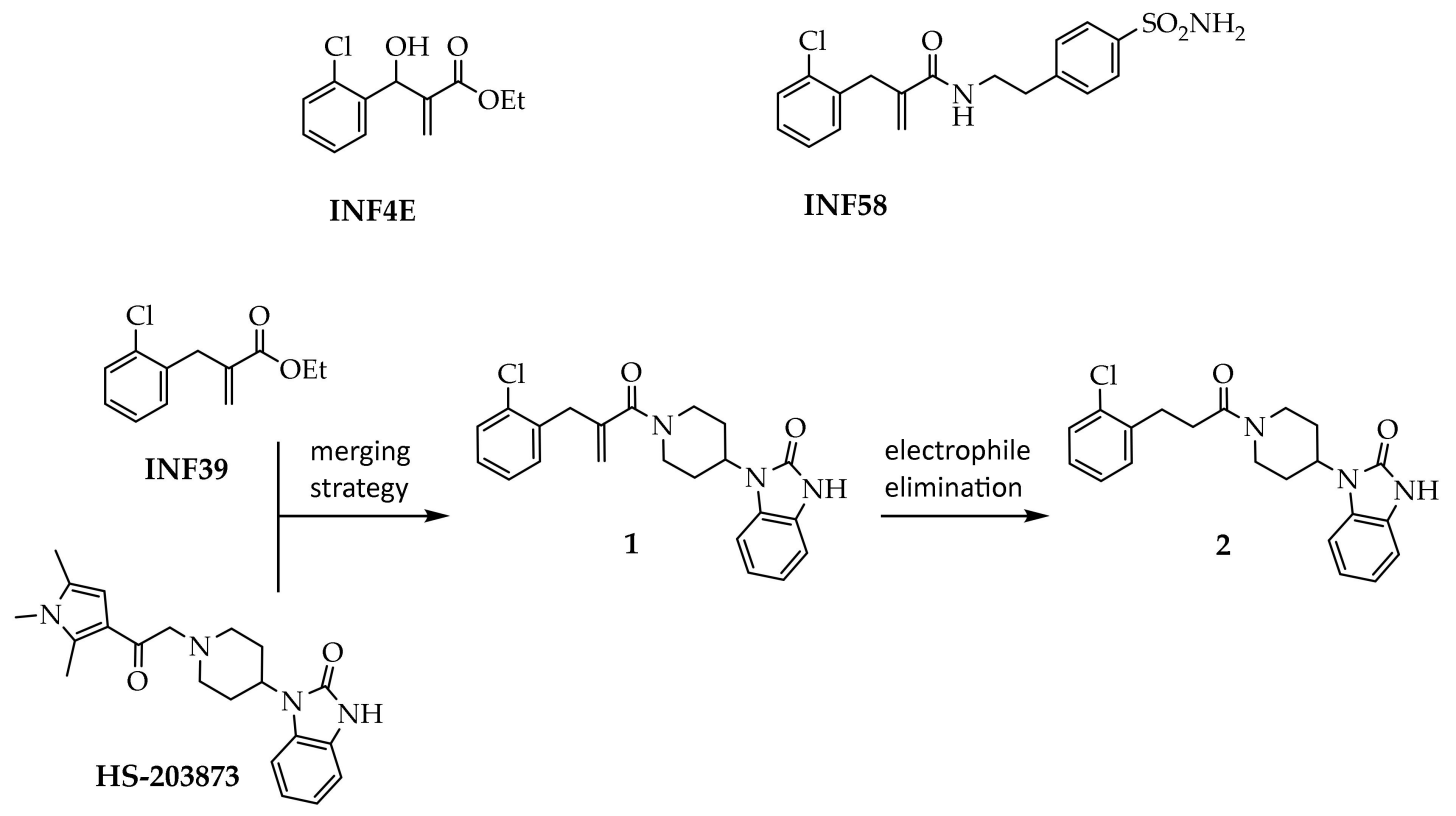
Structures of previously developed NLRP3 inhibitors INF4E, INF39, INF58 and newly designed model compounds **1** and **2**.

Recently, our research group developed covalent NLRP3 inhibitors based on the acrylic acid scaffold (e.g., INF4E, INF39, INF58; Figure 1). These compounds bear an electrophilic moiety with a properly tuned reactivity toward nucleophiles and are able to block NLRP3 activation, NLRP3-dependent pyroptosis and the consequent release of IL-1β [41,42,43]. One compound, namely INF39, proved able to prevent DNBS-induced colitis in vivo after oral administration in mice at both 50 and 25 mg/kg [43,44]. INF39 inhibited ATP hydrolysis by isolated recombinant NLRP3 and prevented conformational changes necessary for NLRP3 activation in NLRP3 expressing HEK293 cells [43]. A recent report demonstrated that INF39 is also able to inhibit the NEK7–NLRP3 interaction attenuating NLRP3 assembly in macrophages [45].

A benzo[*d*]imidazole-2-one derivative, HS203873 (Figure 1), was recently identified using a fluorescent-linked enzyme chemoproteomic strategy (FLECS). HS203873 was able to bind to NLRP3 and its ability to inhibit NLRP3 activation and IL-1β release in differentiated THP-1 cells was confirmed [46]. To discover new molecular scaffolds endowed with NLRP3 inhibition potential, we applied a pharmacophore-hybridization strategy based on merging two molecular probes: the acrylate INF39 and HS203873. The latter molecules have been conjugated to obtain compound **1**, bearing an electrophilic acrylamide substructure and is still able to inhibit pyroptotic cell death and IL-1β release in human macrophages. To investigate the possibility of generating compounds devoid of electrophilic properties, and thus to obtain non-covalent NLRP3 inhibitors, derivative **2** was synthesised. Since compound **2** showed reduced activity, we further modulated this scaffold to design more active non-covalent compounds able to block NLRP3 inflammasome activity. 

**Figure 2:**
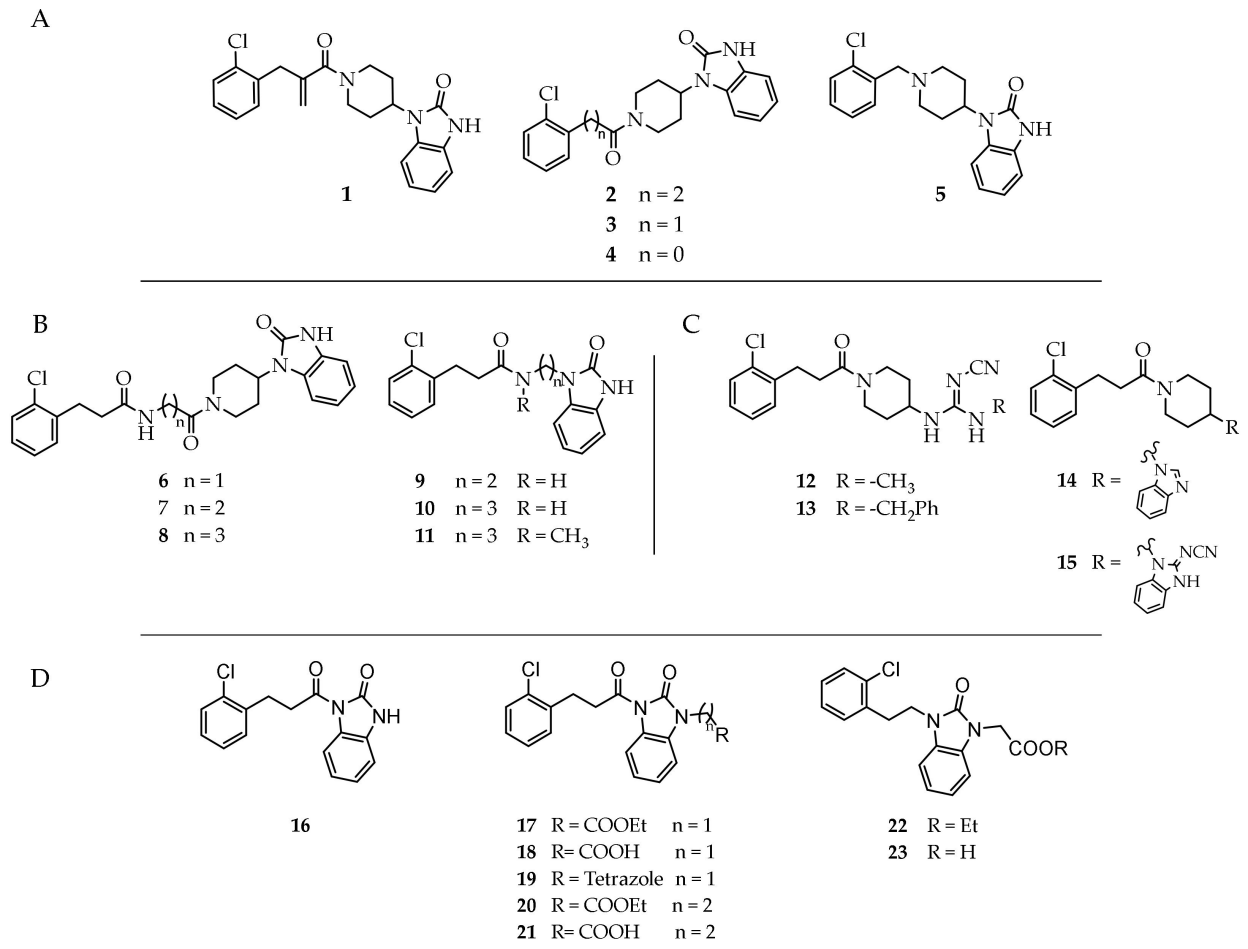
Chemical structures of synthesised compounds. (**A**) Modulation of the aliphatic chain linking the 2-chlorobenzene moiety to the piperidine ring (**1**–**5**); (**B**) Modulation of the amide substituent and of the benzoimidazol-2-one linker (**6**–**11**); (**C**) Replacement of the benzimidazol-2-one moiety with urea-like substructures (**12**–**15**); (**D**) Elimination of the piperidine ring and benzoimidazol-2-one functionalization (**16**–**23**).

In this work, we describe the synthesis and the preliminary in vitro pharmacological screening of a new series of benzo[d]imidazole-2-one derivatives (Figure 2) as potential inhibitors of NLRP3-dependent IL-1β release and pyroptotic cell death in differentiated THP-1 cells, we report data on the ability of the selected compounds to attenuate the ATPase activity of immobilised NLRP3 protein, and we present a possible model of interaction with NLRP3 protein.

## 2. Results and Discussion

### 2.1. Chemistry

**Scheme 1:**
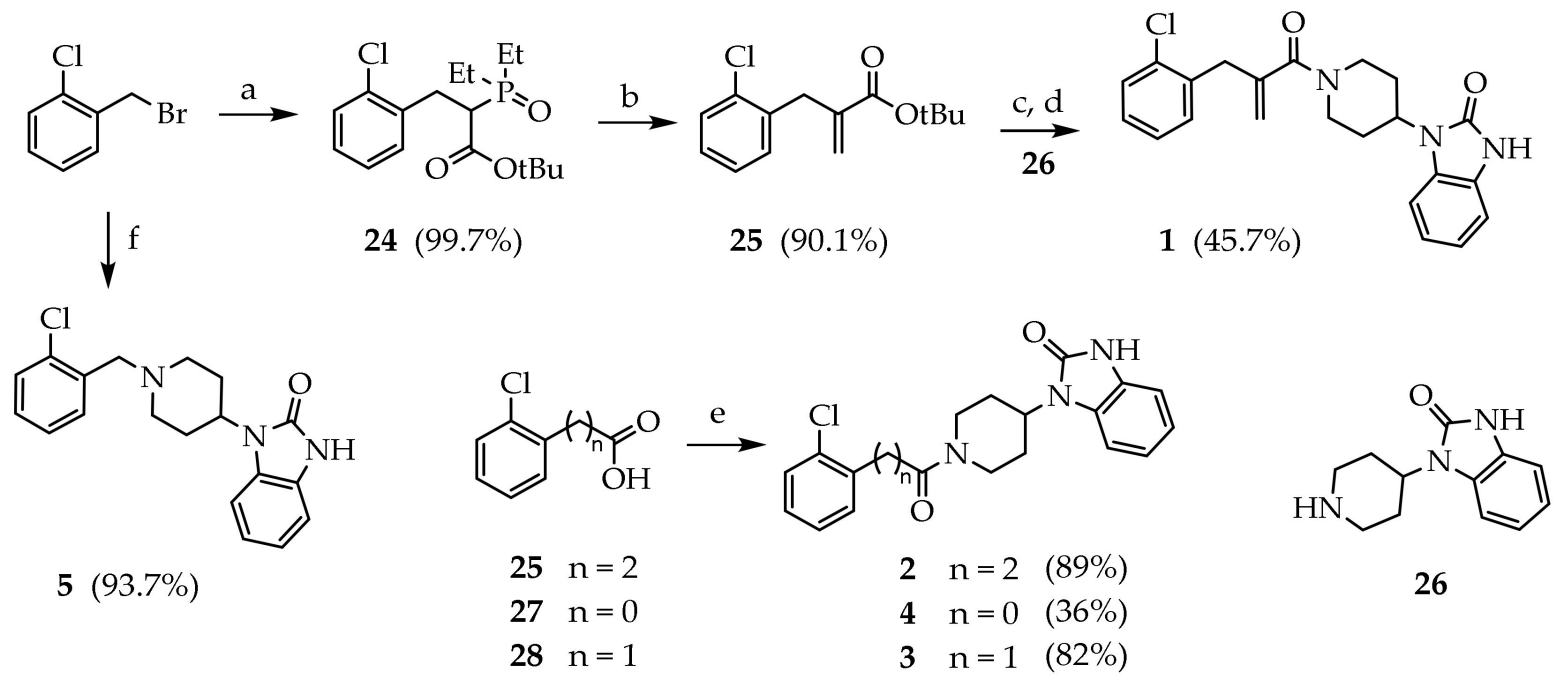
Reagents and conditions: (a) NaH (1 eq), tert-butyl diethylphosphonoacetate (1.2 eq) in DMF 0 °C to rt, 2 h; (b) paraformaldehyde (15 eq), K_2_CO_3_ (2 eq) in water, 12 h; (c) TFA/DCM 10%, 18 h; (d) **26** (1.1 eq), HBTU (1.5 eq), HOBt (0.1 eq), DIPEA (3 eq) in DMF, 18 h; (e) CDI (1 eq), CH_2_Cl_2_, rt, 30′; **26** (1 eq), rt, 16 h. (f) **26** (1 eq), TEA (1 eq) in ACN 2 h.

The reference compound **1** was synthesised according to Scheme 1. The intermediate phosphonate **24** was obtained by the reaction of p-chlorobenzyl bromide with tert-butyl diethylphosphonoacetate in the presence of sodium hydride and then reacted with formaldehyde in basic aqueous solution to yield compound **25**. After the deprotection of t-butyl ester with trifluoroacetic acid (TFA) 10% in CH_2_Cl_2_, the resulting acid was coupled with commercially available 1-(piperidin-4-yl)-2,3-dihydro-1,3-benzodiazol-2-one (**26**) using hydroxybenzotriazole (HOBt) and *N*,*N*,*N*′,*N*′-Tetramethyl-*O*-(1*H*-benzotriazol-1-yl)uronium hexafluorophosphate (HBTU) as the activating agents and diisopropylethylamine (DIPEA) as the base to yield **1** in a 23% overall yield. Compounds **2**–**4** were obtained by coupling of the appropriate carboxylic acid with **26**. In this case, carbonyldiimidazole (CDI) in CH_2_Cl_2_ was used to promote the amide bond formation. 

Derivative **5**, lacking the amide function, was obtained in high yield by alkylation of 2-chlorobenzyl bromide with **26** in basic medium (triethylamine, TEA) in acetonitrile (ACN) as reaction solvent.

**Scheme 2:**
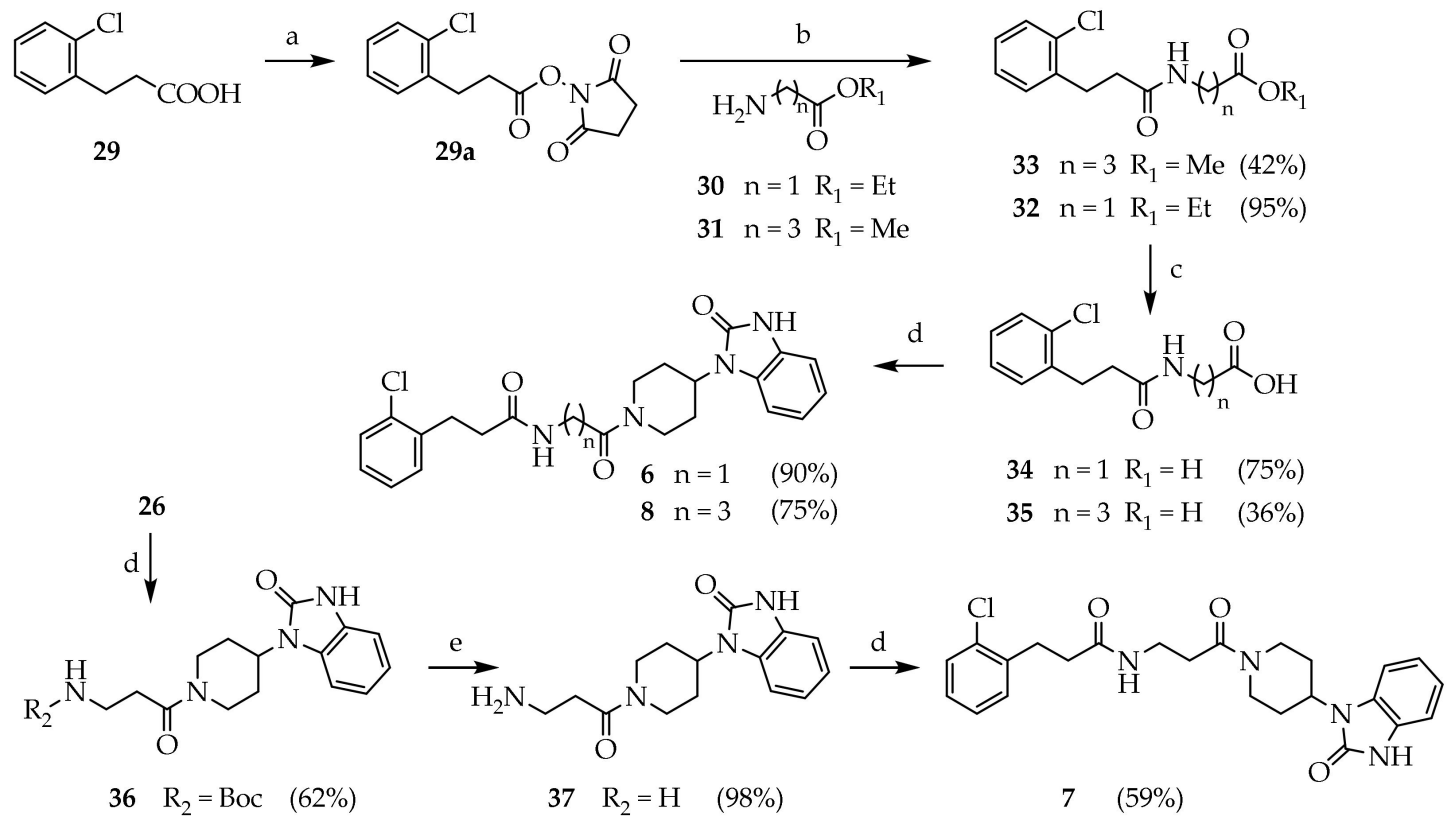
Reagents and conditions: (a) DCC (1 eq), NHS (1.5 eq), THF, 0 °C to rt, 18 h. (b) R-NH_2_ (1.1 eq), Et_3_N (3 eq), CH_2_Cl_2_, rt, 18 h. (c) NaOH 2.5 M (2.5 eq), EtOH or MeOH, rt, 18 h. (d) HBTU (1.5 eq), HOBt (0.15 eq), DIPEA (3 eq), DMF, rt, 18 h. (e) TFA/DCM 10%, rt, 18 h.

The second series of molecules (derivatives **6**–**8**), bearing a spacer between the amide group and the piperidine ring, was synthesised using the synthetic approaches depicted in Scheme 2. The acid **29** was converted into its O-hydroxysuccinimide ester **29a** using dicyclohexylcarbodiimide (DCC) and *N*-hydroxysuccinimide (NHS) and then reacted with the aminoesters **30**–**31** to afford the intermediates **32** and **33**, which were hydrolysed in basic medium (NaOH 2.5 M) to give the acids **34** and **35**. These acids were coupled with **26** under the HBTU/HOBt/DIPEA-mediated conditions described above to give the desired derivatives **6** and **8**. The synthesis of **7** was attained with a slightly different procedure. The piperidine derivative **26** was first coupled with Boc-protected β-alanine using HBTU/HOBt/DIPEA as activating agents. The tert-butyl protected intermediate **36** was cleaved in 10% TFA in CH_2_Cl_2_ to afford the free amine **37**, which was subsequently coupled with hydroxysuccinimide ester **29a**, under the previously described conditions to afford **7** in 36% overall yield. 

**Scheme 3:**
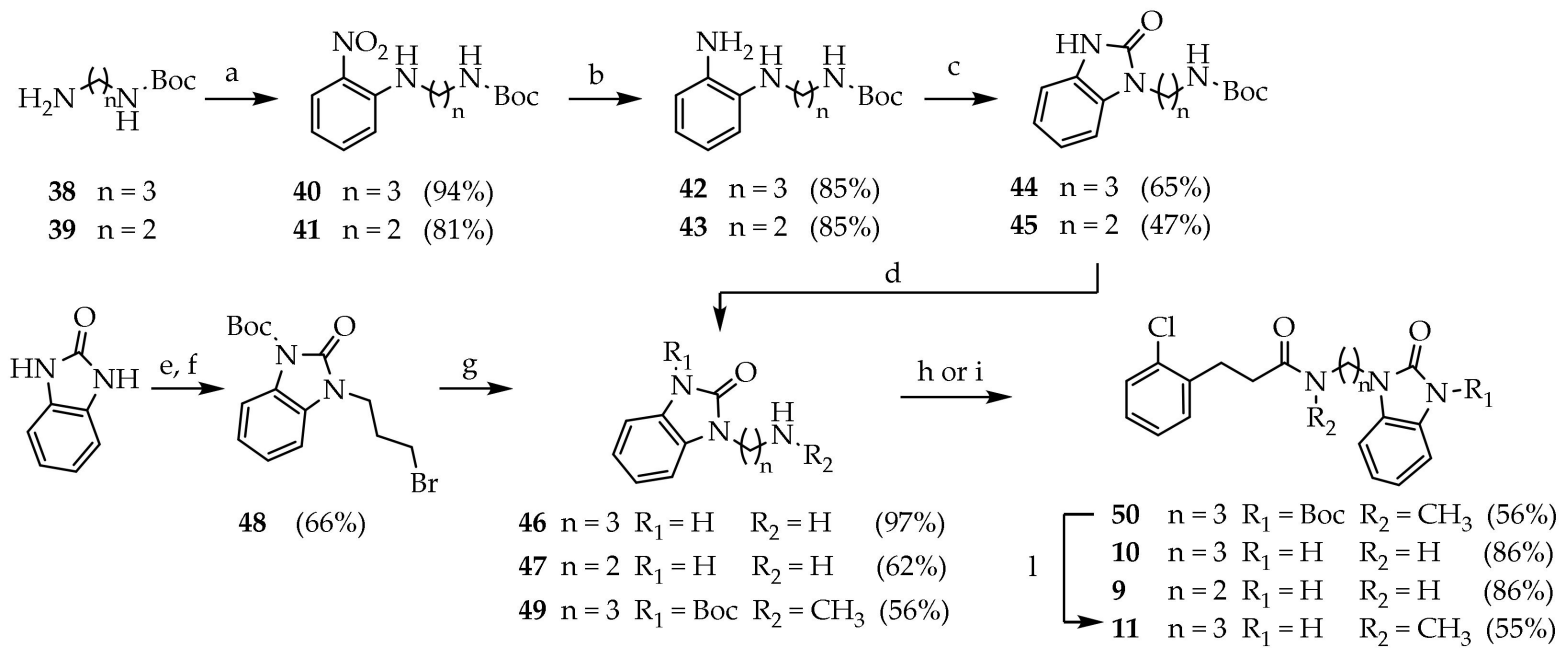
Reagents and conditions: (a) 1-fluoro-2-nitrobenzene (1 eq), **38** or **39** (1.1 eq), K_2_CO_3_ (2.2 eq), DMF, N_2_, 70 °C, 18 h. (b) Pd/C (0.1 eq), H_2_, THF, rt, 18 h. (c) CDI (1.1 eq), THF, rt, 18 h. (d) TFA/DCM 10% (10 eq), rt, 4 h. (e) 1,3-dihydro-2H-benzo[d]imidazol-2-one (1 eq), Boc_2_O (1 eq), NaH (1.1 eq), dry DMF, 0 °C to rt, 18 h. (f) Br(CH_2_)_3_Br (10 eq), K_2_CO_3_ (5 eq), KI (0.1 eq), ACN, rt 18 h. (g) Methylamine 40% aq (20 eq), THF, rt, 1 h. (h) CDI (1 eq), DCM, rt, 30’; **29** (1 eq), rt, 18 h. (i) **29a** (1 eq), DIPEA (1.5 eq), DMF, rt, 18 h. (l) TFA/DCM 10% (10 eq), rt, 18 h.

The third series of molecules, formally derived by the opening of the piperidine ring, was synthesised according to Scheme 3. The aminoalkylbenzimidazol-2-one derivatives **44** and **45** were obtained through a nucleophilic substitution of the fluorine atom on 1-fluoro-2-nitrobenzene using the Boc-protected alkylamines **38** and **39** to afford nitrobenzene derivatives **40** and **41**. Catalytic hydrogenation over Pd/C 10% allowed the reduction of the nitro group to afford aniline derivatives **42** and **43**, which were cyclised using CDI to afford benzimidazolone derivatives **44** and **45** in good overall yields. Deprotection with TFA 10% in CH_2_Cl_2_ afforded the free amines **46** and **47**, which were reacted with the activated ester **29a** in DIPEA/DMF to afford the final compounds **9** and **10**. To obtain the N-methyl substituted derivative **11**, a different approach was used. The benzimidazol-2-one was converted into the intermediate **48** by protection with di-tert-butyldicarbonate and subsequent alkylation with excess 1,3-dibromopropane. Nucleophilic substitution of the bromine with aqueous methylamine afforded **49**, which, in its turn, was coupled with **29** using CDI to afford the protected intermediate **50**. The usual deprotection with TFA 10% in CH_2_Cl_2_ gave the desired **11** in modest yield. 

**Scheme 4:**
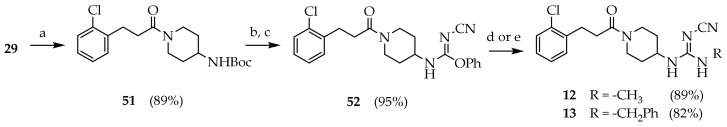
Reagents and conditions: (a) SOCl_2_ (2.5 eq), DMF (0.1 eq), DCM, rt, 2 h; t-butyl piperidin-4-ylcarbamate (1.1 eq), DIPEA (2 eq), DCM, rt, 3 h. (b) TFA/DCM 10% (10 eq), rt, 18 h. (c) diphenyl cyanocarbonimidate (1.1 eq), DCM, rt, 3 h. (d) methylamine (33% in EtOH, 20 eq), rt, 3 h. (e) benzylamine (5 eq), iPrOH, rt, 18 h.

Compounds **12** and **13**, bearing a cyanoguanidine residue in place of the imidazole-2-one ring, were obtained in high yield according to the pathway described in Scheme 4. The acid **29** was converted into the corresponding acyl chloride using SOCl_2_/DMF at room temperature and immediately reacted with commercially available tert-butyl piperidin-4-ylcarbamate to give **51** in nearly quantitative yield. The amino group was deprotected and reacted with diphenyl cyanocarbonimidate to afford the O-phenylisourea derivative **52**. Displacement of the phenoxy group using excess methylamine or benzylamine afforded the final compounds **12** and **13**, respectively.

**Scheme 5:**
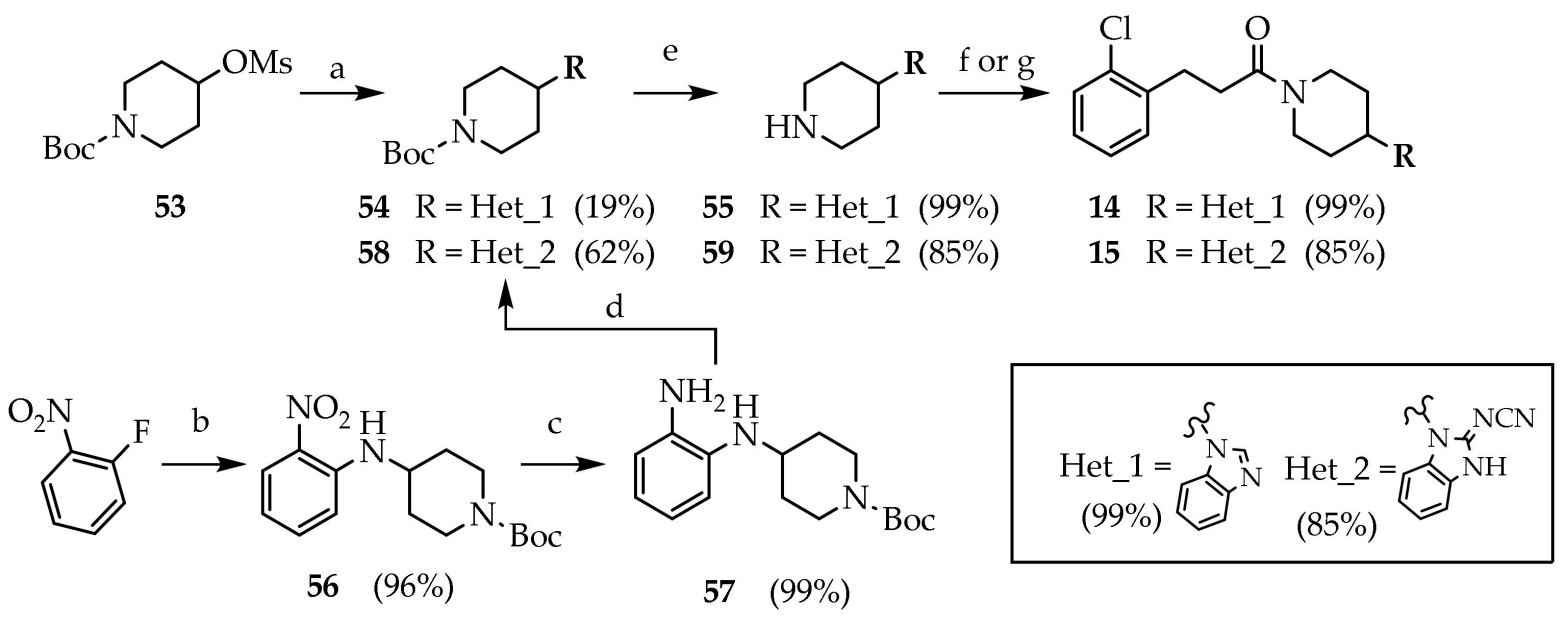
Reagents and conditions: (a) benzimidazole (1 eq), K_2_CO_3_ (2 eq), DMF, 100 °C, 18 h. (b) t-bu 4-aminopiperidine-1-carboxylate (1.4 eq), K_2_CO_3_ (2.2 eq), DMF, N2, 70 °C, 18 h. (c) Pd/C (0.1 eq), H_2_, THF, rt, 18 h. (d) diphenyl cyanocarbonimidate (1 eq), DIPEA (2 eq), ACN, N_2_, reflux, 18 h. (e) TFA/DCM 10% (10 eq), rt, 18 h. (f) **29a** (1 eq), DIPEA (3 eq), DMF, rt, 18 h. (g) **29** (1 eq), CDI (1 eq), DIPEA (5 eq), DCM, rt, 30′; rt, 18 h.

Compounds belonging to the fourth series, bearing the benzimidazole and the cyclic cyanoguanidine moieties at the terminal position, were synthesised using tert-butyl 4-((methylsulfonyl)oxy)piperidine-1-carboxylate (**53**) as the starting material (Scheme 5). Compound **53** was subjected to a nucleophilic substitution using benzimidazole/K_2_CO_3_ in DMF at 100 °C to give **54**, albeit in poor yield. Deprotection and subsequent coupling with the preformed activated ester **29a** afforded the desired **14**. To obtain **15**, the synthesis of the intermediate **57** was needed. This intermediate was obtained through nucleophilic aromatic substitution of the tert-butyl piperidin-4-ylcarbamate on 1-fluoro-2-nitrobenzene to give **56** followed by catalytic hydrogenation of the nitro group to give the substituted benzendiamine derivative **57** in 95% yield over two steps. Cyclization of **57** with diphenyl cyanocarbonimidate afforded tert-butyl 4-(2-(cyanoimino)-2,3-dihydro-1*H*-benzo[d]imidazol-1-yl)piperidine-1-carboxylate (**58**). Finally, Boc-deprotection and CDI-mediated coupling with the acid **29** afforded the desired **15**.

**Scheme 6:**
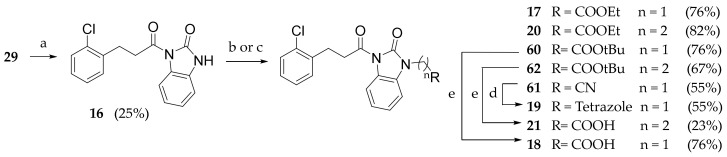
Reagents and conditions: (a) I. **29** (1 eq), SOCl_2_, 85 °C, 1 h. II. benzo[d]imidazole-2-one (1 eq), NaH 60% (1 eq), dry THF, nitrogen atmosphere, rt, 1 h. III. dry DMF, rt, 3 h. (b) DBU (2 eq), ethyl or tBu bromoacetate or bromoacetonitrile (2.5 eq), THF, rt, 18 h. (c) DBU (2 eq), ethyl or tBu acrylate (solvent), rt, 18 h. (d) NaN_3_ (3 eq), NH_4_Cl, DMF, 120 °C, 2 h. (e) TFA (10 eq), DCM, rt, 18 h.

The series of compounds **16**–**21** was designed with the 3-(2-chlorophenyl)-propanoyl moiety directly linked to the benzimidazol-2-one moiety, to generate an acyl-urea substructure. Compounds were synthesised according to Scheme 6. The synthesis started from the deprotonation of the 1,3-dihydro-2*H*-benzo[d]imidazol-2-one, which was achieved with sodium hydride and its reaction with the in-situ-generated 3-(2-chlorophenyl)-propanoyl chloride. By this procedure, we were able to isolate compound **16** in low yield. Compound **16** was then reacted with 1,8-diazabicyclo[5.4.0]undec-7-ene (DBU) and alkylated with the appropriate α-bromo carbonyl derivative (ethyl 2-bromoacetate, tert-butyl 2-bromoacetate or 2-bromoacetonitrile) to give the final compound **17**, the tert-butyl-protected intermediate **60** and the nitrile **61**, respectively. The intermediate **60** was deprotected in TFA 10% to afford the desired acetic acid derivative **18**, while the nitrile **61** was reacted with sodium azide and NH_4_Cl to furnish the tetrazole isostere **19**. The same alkylation using bromoderivatives bearing a longer alkyl chain gave only trace amounts of the desired N-alkylated compounds, therefore compound **16** was alkylated using ethyl or tert-butyl acrylate, in a Michael reaction, to afford **20** and the protected intermediate **62**. The latter was hydrolysed to give the desired **21** in reasonable yield.

**Scheme 7:**
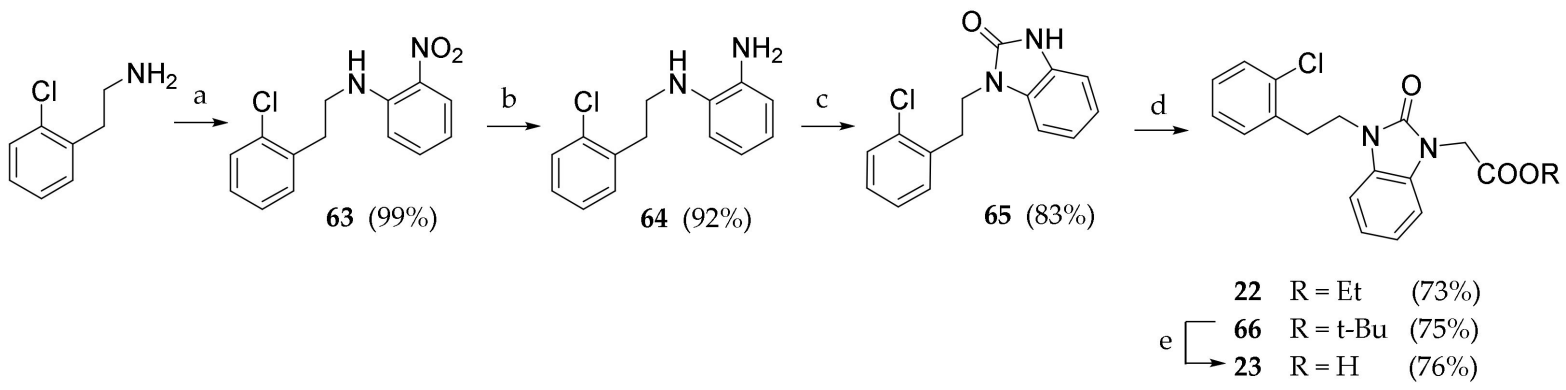
Reagents and conditions: (a) 2-(2-Chlorophenyl)ethylamine (1.3 eq), 1-Fluoro-2-nitrobenzene (1 eq), K_2_CO_3_ (2 eq), DMF, N_2_, 70 °C, 18 h. (b) Pd/C (10%mol), H_2_, THF, rt, 16 h. (c) CDI (2 eq), DCM, rt, 18 h. (d) DBU (2 eq), ethyl or tBu bromoacetate (2.5 eq), THF, rt, 18 h. (e) TFA (10 eq), DCM, rt, 18 h.

Finally, compounds **22** and **23** were synthesised according to Scheme 7. Nucleophilic aromatic substitution with 2-(2-chlorophenyl)ethylamine on 1-fluoro-2-nitrobenzene, followed by reduction and cyclization were performed using the same synthetic steps as described above to afford the intermediate 1-(2-chlorophenethyl)-1,3-dihydro-2*H*-benzo[d]imidazol-2-one (**65**). This intermediate was alkylated in good yields using ethyl 2-bromoacetate and tert-butyl 2-bromoacetate in basic medium (DBU) to afford **22** and **66**, respectively. The latter was then hydrolysed to afford the desired acetic acid derivative **23**.

### 2.2. Biological Evaluation

Different techniques could be used to study NLRP3 inhibitors [47]; the use of differentiated THP-1 cells treated with NLRP3 activating stimuli is suitable for the screening of large series of compounds [48]. All the synthesised compounds (Figure 2) were evaluated for their NLRP3 inhibitory activity by measuring their ability to prevent NLRP3-dependent pyroptosis in differentiated THP-1 cells. THP-1 cells were differentiated into macrophages by treatment with phorbol-myristate acetate (PMA; 50 nM; 24 h) and then treated with lipopolysaccharide (LPS; 10 µg/mL; 4 h) to promote NLRP3 expression. The cells were then incubated with test compounds at the fixed concentration of 10 µM for 1 h. Test compounds were dissolved in serum free medium containing 0.1% DMSO as the vehicle. NLRP3 was activated by the addition of ATP (5 mM), and the pyroptotic cell death was evaluated after 1.5 h by measuring the lactate dehydrogenase (LDH) released in the cell supernatants. The obtained results, expressed as pyroptosis% decrease with respect to vehicle-treated cells, are collected in Table 1. In the same experiments, the release of IL-1β was measured via an ELISA assay and the results, expressed as % inhibition of IL-1β release versus vehicle-treated cells, are reported in Table 1. Finally, the cytotoxicity of the synthesised compounds was evaluated by MTT assay after treatment of THP-1 cells with increasing concentration of test compounds (0.1–100 µM) for 72 h. The results, expressed as TC_50_, are reported in Table 1. Analysis of the obtained results showed that by merging the structure of INF39 with the 1-(piperidin-4-yl)-2,3-dihydro-1,3-benzoimidazol-2-one moiety present in HS-203873, to obtain **1**, a compound able to prevent pyroptosis (24.9 ± 6.3%) and IL-1β release (19.4 ± 0.4%) to a similar extent at 10 µM was generated. When the electrophilic substructure was eliminated (compound **2**), the anti-pyroptotic activity dropped down to 14.9 ± 5.8% inhibition (*p* = 0.198); however, the effect of compound **2** appeared to be concentration-dependent as the pyroptosis inhibition increased to 29.1 ± 4.8% (*p*< 0.05) at 50 µM. The structural modulation of compound **2** was then carried out in order to understand whether it was possible to restore/increase the activity without the re-introduction of a Michael acceptor substructure.

We first modulated the carbon chain linker used to conjugate the 2-chlorobenzene moiety to the piperidine ring by synthesising three model compounds (derivatives **3**–**5**, Figure 2). Among these compounds, only derivative **3**, bearing an acetamide bridge, showed an anti-pyroptotic activity (Table 1), while further shortening of the linker (**4**) or elimination of the carbonyl group (**5**) led to inactive compounds at the tested concentration (10 µM). When the 1-(piperidin-4-yl)-2,3-dihydro-1,3-benzodiazol-2-one moiety was conjugated with 2-chlorophenylacetamide through a C1-C3 spacer (derivatives **6**–**8**, Figure 2), two compounds endowed with the ability to prevent about 35% of pyroptotic cell death and to decrease IL-1β by approximately 18–21% were obtained (compounds **6**, **7**; Figure 2, Table 1). Compound **2** was also modulated by opening the piperidine ring linking the benzo[d]imidazole-2-one to the phenylacetamido moiety in order to check whether the removal of conformational constrains (i.e., increased flexibility) could improve the interaction with the putative target. To this aim, compounds **9**–**11** (Figure 2) were synthesised. Results showed that only compound **9** maintained the anti-pyroptotic activity (39.2 ± 6.6% inhibition) and the IL-1β inhibition (20.3 ± 1.3%), while **10** and **11** were inactive at 10 µM.

To understand the role played by the benzo[d]imidazole-2-one substructure, compounds **12**–**15** (Scheme 4 and Scheme 5) were synthesised. The replacement of benzimidazol-2-one with a benzimidazole afforded an inactive compound (derivative **14**, Figure 2). The use of a cyanoguanidine group in place of the ureidic moiety present in the benzoimidazol-2-one ring gave interesting results. Compounds **12** and **13** (Figure 2), bearing a methyl- and benzyl-substituted cyanoguanidine residue at the terminal position were able to inhibit both NLRP3-dependent pyroptosis and IL-1β release in LPS/ATP-treated macrophages. Surprisingly, compound **15**, bearing the di-substituted cyanoguanidine constrained into a 1,3-dihydro-2*H*-benzo[d]imidazol-2-ylidene)cyanamide was inactive (Table 1).

The last series of compounds encompasses derivatives **16**–**23** (Figure 2). These compounds were deprived of the piperidine ring and the benzoimidazol-2-one structure was directly linked to the 2-chlorophenyl substructure through a propanoyl or an ethyl linker. In this series of compounds, the benzimidazol-2-one ring was substituted with an acidic group or with an ethyl ester, used as the carboxylic acid prodrug, at the terminal nitrogen. The biological results, reported in Table 1, showed that the 1-(3-(2-chlorophenyl)propanoyl)-1,3-dihydro-2*H*-benzo[d]imidazol-2-one scaffold could be the minimal requirement for NLRP3 inhibition (compound **16**: 37.7 ± 7.6% pyroptosis reduction; 14.9 ± 8.8% IL-1β inhibition). When this scaffold was functionalised with an acetic acid residue (**18**) or a tetrazol-5-yl-methyl residue (**19**), two active compounds were obtained (Table 1). Interestingly, the conversion of **18** into the corresponding ethyl ester **17** maintained the activity as well as the removal of the carbonyl group in compound **23**. When the carboxylic group was further spaced apart from the benzoimidazol-2-one ring, the activity was suppressed (derivative **21**). It is difficult to rationalize the behaviour of compounds **20** and **22**.

All the synthesised compounds did not show relevant toxicity in THP-1 cells (Table 1), with the partial exception of compound **1** (TC_50_ 32.9 ± 19 µM), possibly because of the electrophilic character of this derivative.

**Figure 3:**
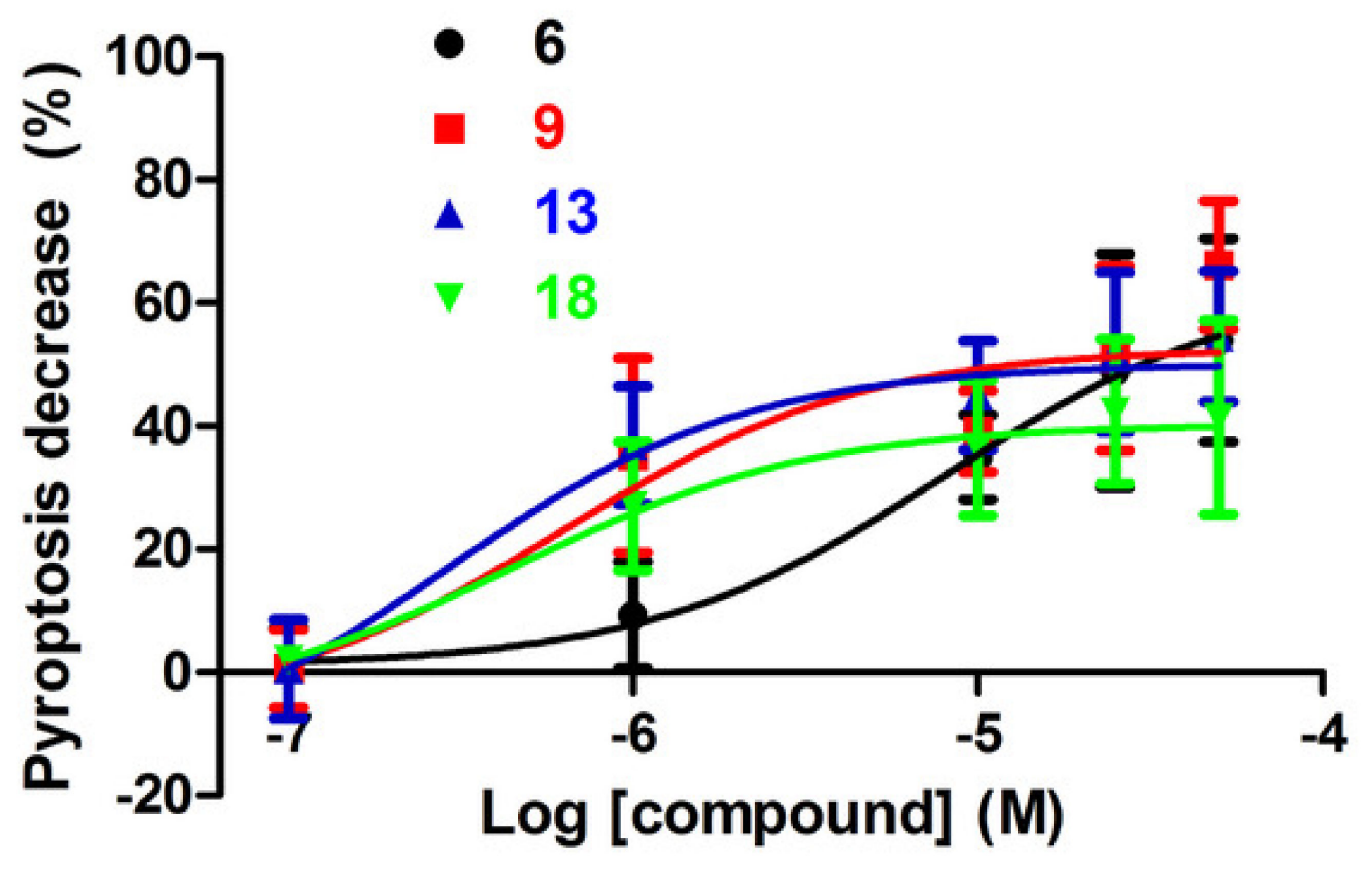
Concentration-response curves for the effects of compounds **6**, **9**, **13** and **18** on the ATP-triggered cell death of differentiated THP-1 cells. PMA-differentiated and LPS-primed THP-1 cells were pre-treated with vehicle alone or indicated compound. Each compound was added at increasing concentrations (0.1−50 μM) 1 h before exposure to ATP (5 mM; 90 min). LDH activity was measured in the collected supernatant by a colorimetric assay. Data are expressed as decrease of cell death vs. vehicle alone and are the mean ± SEM of at least three experiments run in triplicate.

From the preliminary screening, compounds **6**, **9**, **13** and **18** were selected for further biological evaluation. The anti-pyroptotic effect of the selected compounds was determined in human macrophages using increasing concentrations (0.1–50 µM) of test compounds (Figure 3). In these conditions, **6**, **9**, **13** and **18** showed a conc.-dependent inhibition of NLRP3-dependent pyroptosis with a maximal inhibition ranging between 40 and 60%. Interestingly, compounds **9**, **13** and **18** maintained a significant ability to inhibit pyroptosis down to 1 µM (approx. 20–30% inhibition).

### 2.3. Inhibition of NLRP3 ATPase Activity

**Figure 4:**
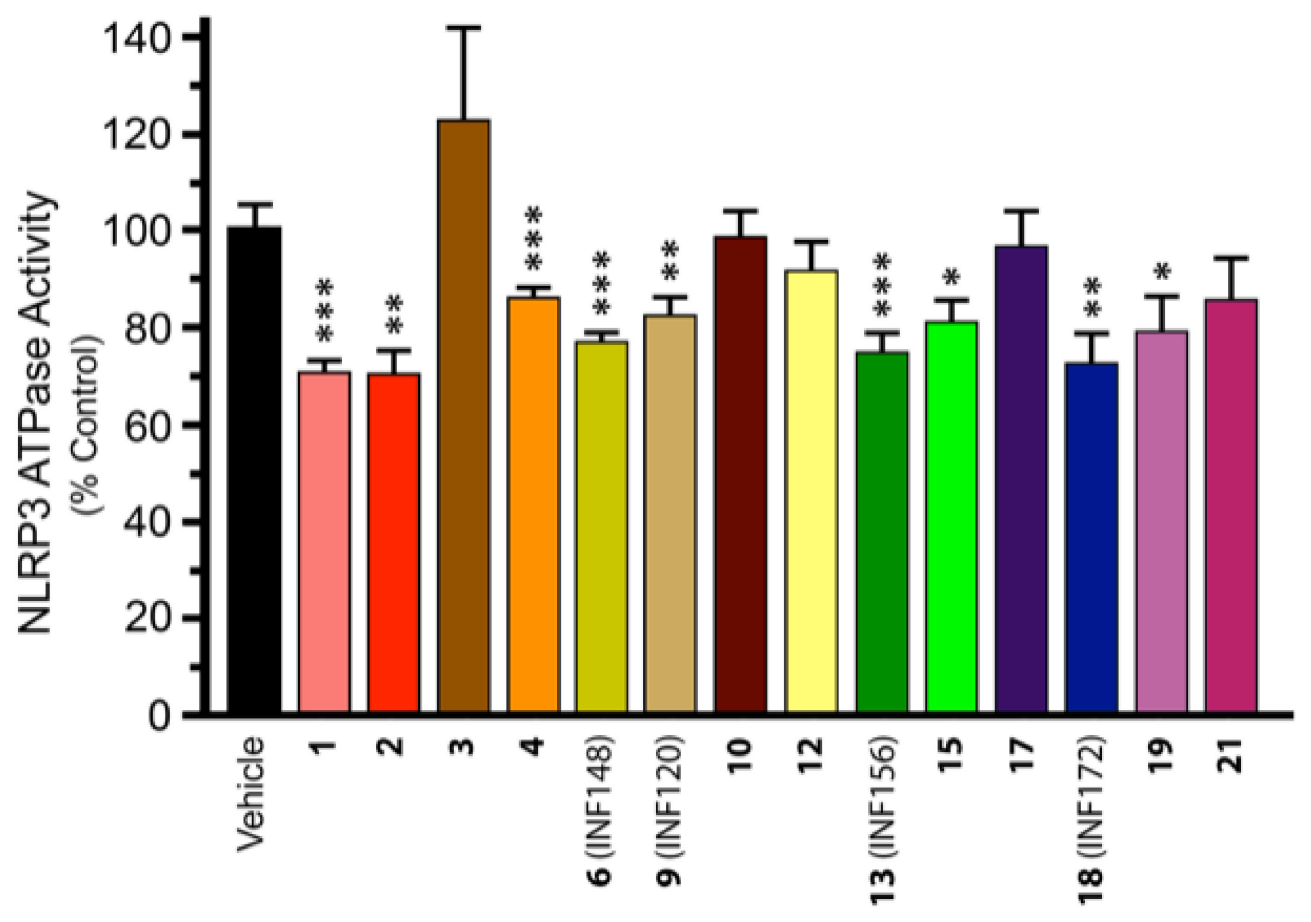
Inhibition of NLRP3 ATPase activity by selected compounds. Inhibition was calculated from the relative luminescence units (RLU) obtained with each compound/RLU of vehicle control. All compounds were added at concentrations of 100 μM and the ATP concentration in all assays was 800 μM. Mean ± SEM; Significantly different from vehicle control using unpaired Student’s *t*-test with Welsch’s correction. * *p* ≤ 0.05, ** *p* ≤ < 0.005, *** *p* ≤ 0.0005; *n* = 4 assays per condition.

Selected compounds were further assessed for their ability to directly inhibit the ATP hydrolysis activity of the NLRP3 enzyme in vitro. The in vitro assays of NLRP3 ATPase activity were completed using 100 μM compound concentrations in order to provide the maximal inhibitory potential for the pre-incubation conditions used in the assay. We choose a rather high concentration for the inhibitors, expecting a low general effect if they were acting in a competitive manner toward substrate ATP. The collected results, expressed as % activity decrease with respect to vehicle-treatment, are presented in Figure 4. Compound **1**, a conjugation of the acrylate INF39 and HS203873, displayed inhibitory potential toward the enzymatic activity of NLRP3 with a 28.4 ± 2.6% reduction in ATP hydrolysis relative to vehicle control. Likewise, derivative **2** was also able to suppress the ATPase activity by 28.6 ± 5.5%. Compounds **6** (INF148), **9** (INF120), **13** (INF156) and **18** (INF172) demonstrated similar inhibitory potential when used in the in vitro ATPase assay. In this case, the compounds were associated with 22.5 ± 1.4%, 18.3 ± 3.2%, 26.5 ± 3.1% and 27.2 ± 6.3% inhibition, respectively.

Shortening the carbon-chain linker appeared to reduce (compound **4**) or abolish (compound **3**) the inhibitory potential when compared to **1** and **2**. When the piperidine ring in compound **2** was replaced by a three-methylene chain (compound **10**) no effect on ATPase inhibition was detected, while the use of a two-methylene chain (compound **9**, INF120) restored the ATPase inhibition. Among the three cyanoguanidine-containing compounds **12, 13** (INF156) and **15**, only compounds **13** and **15** showed a significant inhibition of ATPase activity. Finally, among the compounds belonging to series D (Figure 2), the ethyl ester derivative **17** was inactive while the corresponding acid **18** (INF172) was able to reduce ATPase activity. In this series of derivatives, both the lengthening of the chain bearing the COOH group (compound **21**) or the replacement of the COOH with a tetrazol-5-yl (compound **19**) reduced the inhibitory potential. This observation indicates that the presence of an acidic function in a correct spatial orientation might be important for the inhibition of the ATPase activity in this series of NLRP3 inhibitors.

**Figure 5:**
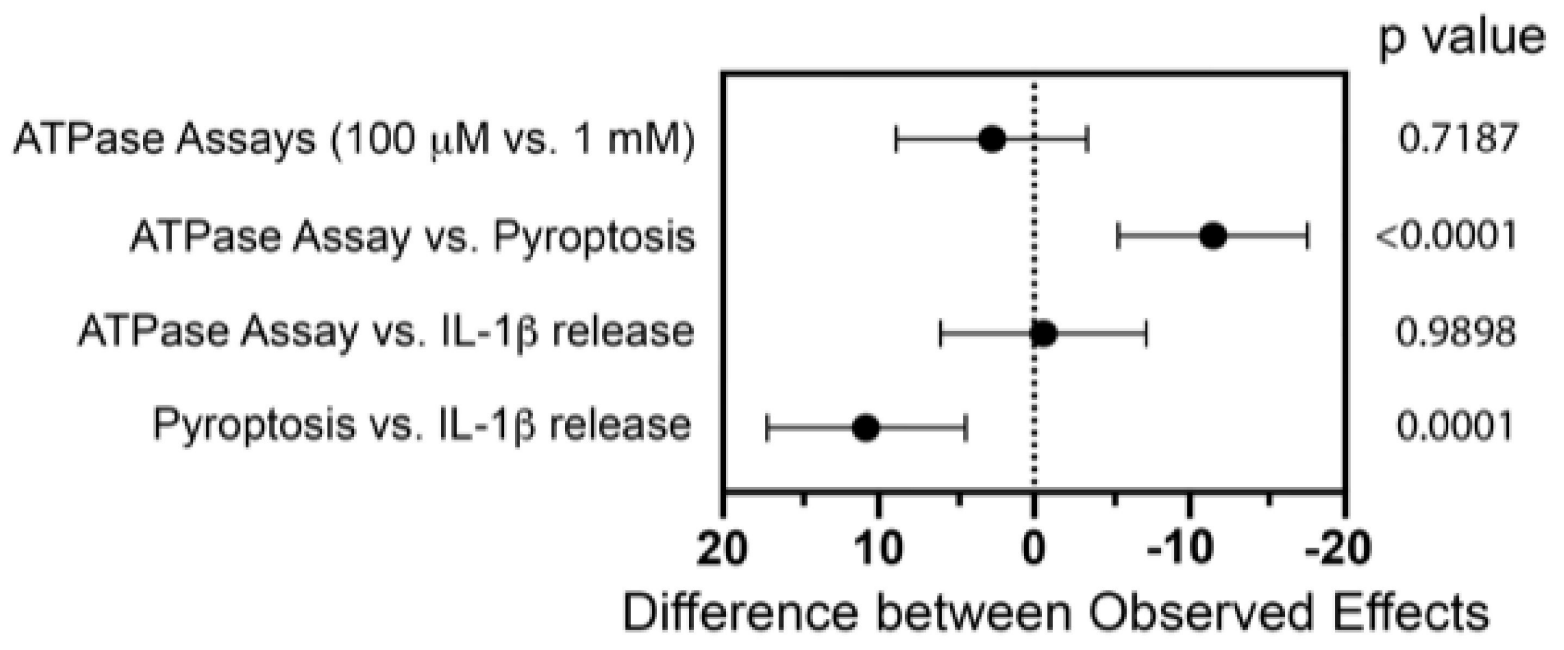
Effect of the experimental method on NLRP3 inflammasome attenuation. The impact of selected compounds on inflammasome outputs (i.e., pyroptosis, ATPase activity, IL-1β secretion) was assessed by two-way ANOVA with Tukey’s multiple comparison test showing 95% CI. No difference between the observed effects suggests the INF compound administration was similar in outcome. Only those compounds which had been analysed by all three methods were included in the statistical analysis.

The inhibitory potentials of selected compounds were also assessed at 1 mM (data not shown). The effective inhibition was found to be similar at both concentrations (Figure 5). Specifically, no significant differences were identified between the two concentrations for compounds **6** (INF148), **9** (INF120), **13** (INF156) and **18** (INF172). A non-competitive inhibition vs. ATP, together with a low apparent Ki might be at the basis of this behaviour. Moreover, we completed a comparison of the impact of the different experimental methods (i.e., IL-1β maturation, ATPase inhibition and pyroptosis). Although not all compounds were examined by all experimental methods, those compounds demonstrating inhibitory potential in the ATPase assay were also associated with effective attenuation of IL-1β release (Figure 5). However, compounds that suppressed pyroptosis were generally not well aligned with inhibitory effects on either IL-1β secretion or enzymatic ATPase activity.

### 2.4. Molecular Modelling

**Figure 6:**
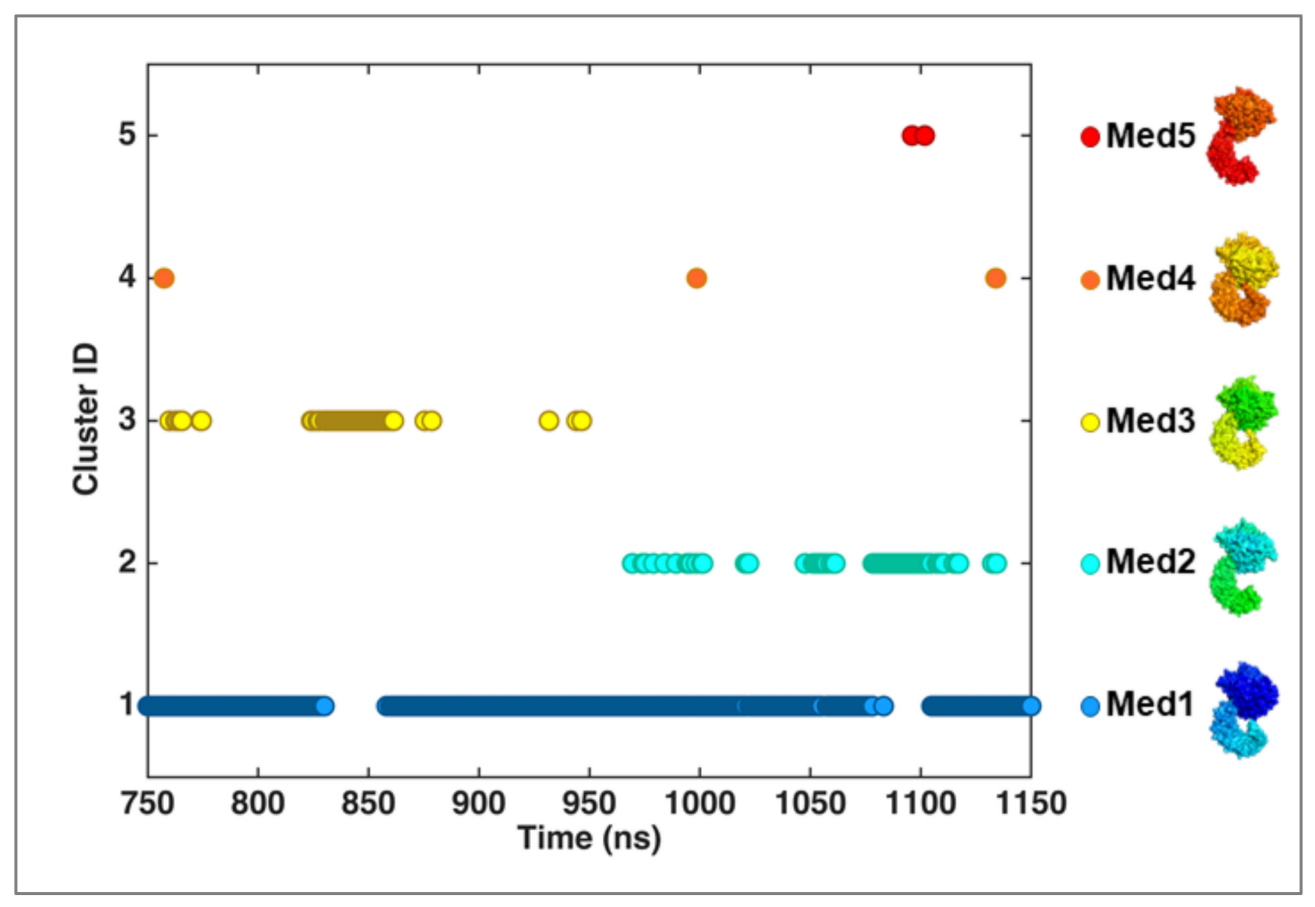
Cluster ID over simulation time (ns). For comparison, a surface representation of the medoid structure belonging to each cluster ID is displayed on the right.

We next investigated the potential binding mode of the model compounds (**6**, **9**, **13** and **18**) with NLRP3. The NLRP3 protein in complex with ADP and Mg^2+^ ion was modelled on PDB entry 6NPY and submitted to extended (1.150 µs) plain molecular dynamics (MD). The Root Mean Square Deviation (RMSD) of the backbone atoms was calculated for checking the structural convergence of the protein (Figure S1). As the structure was obtained by homology modelling (see Methods), the average RMSD along simulation time was quite high. According to the RMSD plot, the 750–1150 ns time frame showed a less dispersed profile, suggesting the achievement of a more stable conformation. To better investigate the structural convergence of the trajectory, the covariance matrix was calculated and diagonalised. Then, eigenvectors were sorted according to their eigenvalues. The resulting principal component analysis (PCA) plot highlighted a large movement along the first eigenvector during the first part of the trajectory, while the last part of the trajectory showed a large displacement along the second eigenvector (Figure S2). The order of magnitude of the principal modes was quite high, reflecting NLRP3 intrinsic flexibility. In agreement with the RMSD analysis, the last part of the simulation (750–1150 ns) explored a less dispersed scattering in the essential space, and finally concentrated in an ellipsoid restricted area during the last 150 ns. Finally, the RMSD matrix was calculated, for assessing the difference among the conformations explored during the MD simulation. Consistently with the previous analyses, the matrix showed a fragmented pattern of minor conformational clusters until approximately 750 ns, while from 750–1150 ns, macro clusters were detected (Figure S3). In accordance with the results shown by the RMSD plot, PCA and RMSD matrices, we retained the 750–1150 ns time frame of the MD simulation to perform the clustering with the Gromos method and a 0.4 nm cut-off. The cluster analysis highlighted five major clusters in the 750–1150 ns timeframe; the representative structure for each of these was extracted. Figure 6 shows the distribution of the five clusters along simulation time, highlighting that the first, second and third cluster well represent the trajectory. The medoid structure of each of these three clusters was extracted and used as target structure for docking studies. From here on, these medoids will be referred to as Med1, Med2 and Med3, i.e., the representative structure of the first, second and third most populated clusters, respectively.

**Figure 7:**
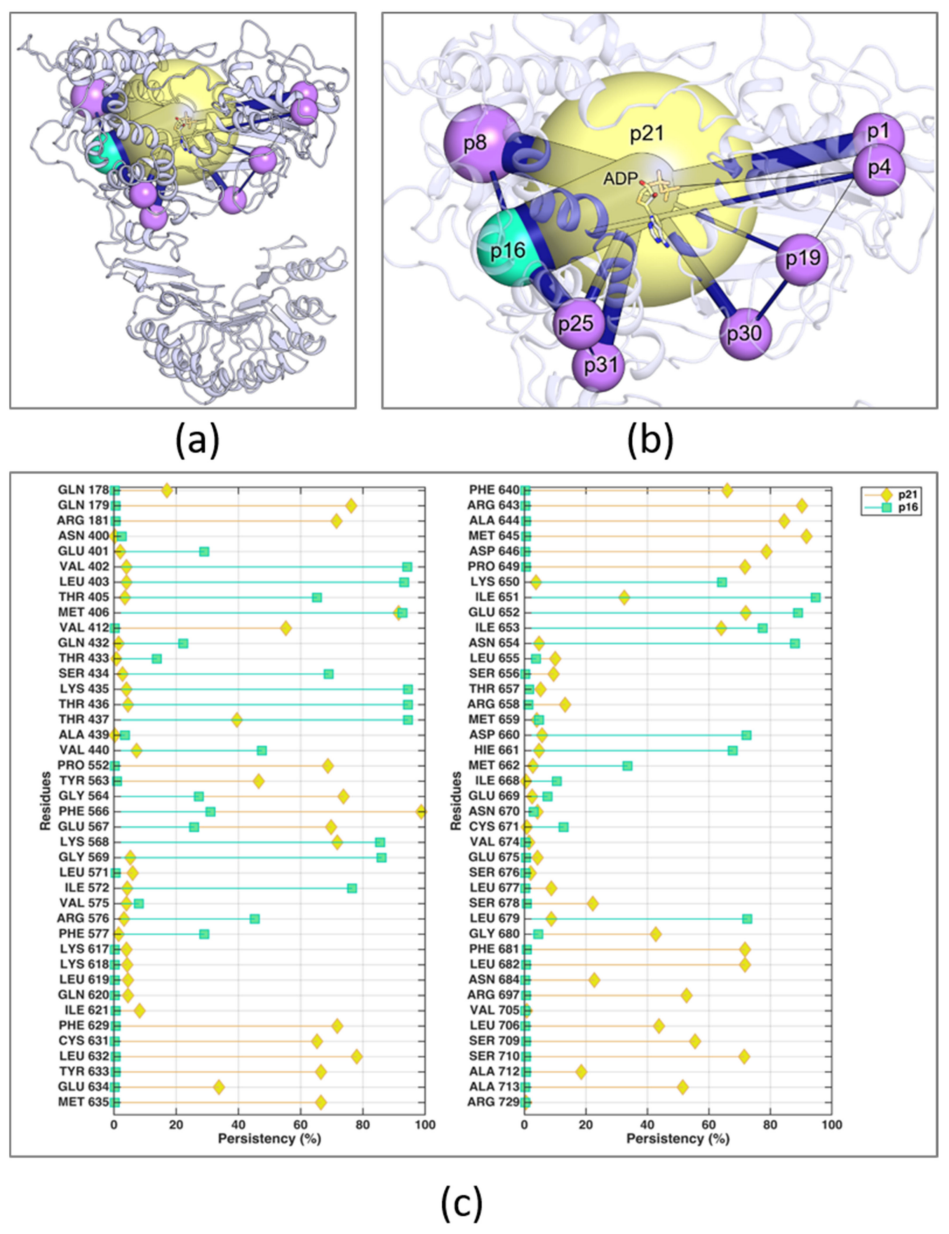
(**a**) Dynamic pocket network. Pockets with 90% or higher persistency are represented as spheres, with the sphere radius being proportional to pocket volume. Each pocket represents a node in the network, and dark blue edges represent the cross talk between pockets, with their thickness being proportional to the degree of correlation. Protein and ADP molecules are reported as cartoons and sticks, respectively. (**b**) Close-up of the NACHT domain. Pockets with 90% or higher persistency are labelled according to their pocket ID. ADP pocket (p21) is highlighted in yellow, p16 pocket is coloured in bright green. (**c**) Communication between the ADP pocket and p16. Persistency (% of frames) of residues belonging to the ADP pocket (p21, yellow circles) compared to those belonging to p16 (green squares).

The quality of the NLRP3 model was assessed by calculating the Ramachandran plot for the initial structure and for the medoids extracted from the trajectory and used for subsequent docking studies (Figures S4–S7). With respect to the initial model, MD structures showed a decreased number of outliers and an increased percentage of residues in allowed/favoured regions (Table S1). We thus analysed the three medoids looking for pockets close to the ADP binding site in Med1, Med2 and Med3. Pockets were calculated with FLAPsite to assess the extent of the cavity (Figure S8). The volume of the ADP pocket greatly varies along the MD simulation, as the ADP molecule is bound between two subdomains that are known to undergo drastic reorientation upon protein activation [2]. In our MD simulation, the ADP pocket merged (Figure S8, panel a) or split (Figure S8, panels b and c) to other minor pockets, considerably changing its shape and extent. We extensively investigated the docking pose of the mentioned compounds in this variable and very large ADP pocket, which we separated into several sub-pockets identified by corresponding centroids having a radius of 8–10 Å (Table S2). Since docking studies in these pockets showed no consensus poses and low scores, we investigated all the pocketomes in NLRP3 to check whether other pockets in communication with the ADP site could better and more consistently accommodate the compounds. Pockets coupled to the ADP site along the MD trajectory (750–1150 ns timeframe) were detected with Pocketron (see Methods section), and among these, the ones showing less than 90% persistency were discarded. The retained cavities with 90% or more persistency, namely p1, p4, p8, p16, p25, p30 and p31, were further investigated in docking studies (Figure 7).

The compounds were docked in these pockets, searching for consensus among the five top-ranked poses. The best results were achieved in pocket p16, which showed the highest dynamic correlation with the ADP pocket. The cross talk between the ADP pocket and surrounding pockets was monitored using the Pocketron tool implemented in the BiKi software suite as detailed in the Methods section. Overall, residues Met408, Thr439, Lys570, Glu520 and Ile521 are frequently exchanged between the ADP pocket and p16, highlighting a hydrophobic communication path between these two sites (Figure 7c). Consequently, the binding of ligands at the p16 site could affect this cross talk and have an impact on the ADP binding site. Docking of **9** (INF120) and other model compounds **6**, **13** and **18** in p16 showed a consensus in the overall binding poses (Figures S9–S11). A hydrophobic sub-pocket lined by residues Val404, Leu405, Met408 and Ile653 hosts the 2-chlorophenyl moiety of ligands. The amide linker is usually engaged in variable polar contacts with surrounding residues such as Thr439 and the backbone of Lys437 and Asn656. The environment interacting with the benzimidazole-2-one moiety is mainly composed by the basic side chains of residues Arg578, His663 and Lys437, which open the door to the modulation with hydrogen bond acceptor substituents as done in **18** (INF172). Additional hydrophobic interactions of the benzimidazol-2-one involve Leu681.

To better investigate how **9** binds to p16, induced fit docking studies were carried out, as described in the Methods section. The best outcome was related to docking in Med3, in which the LRR domain appears closer to the NACHT domain than in Med1 and Med2.

**Figure 8:**
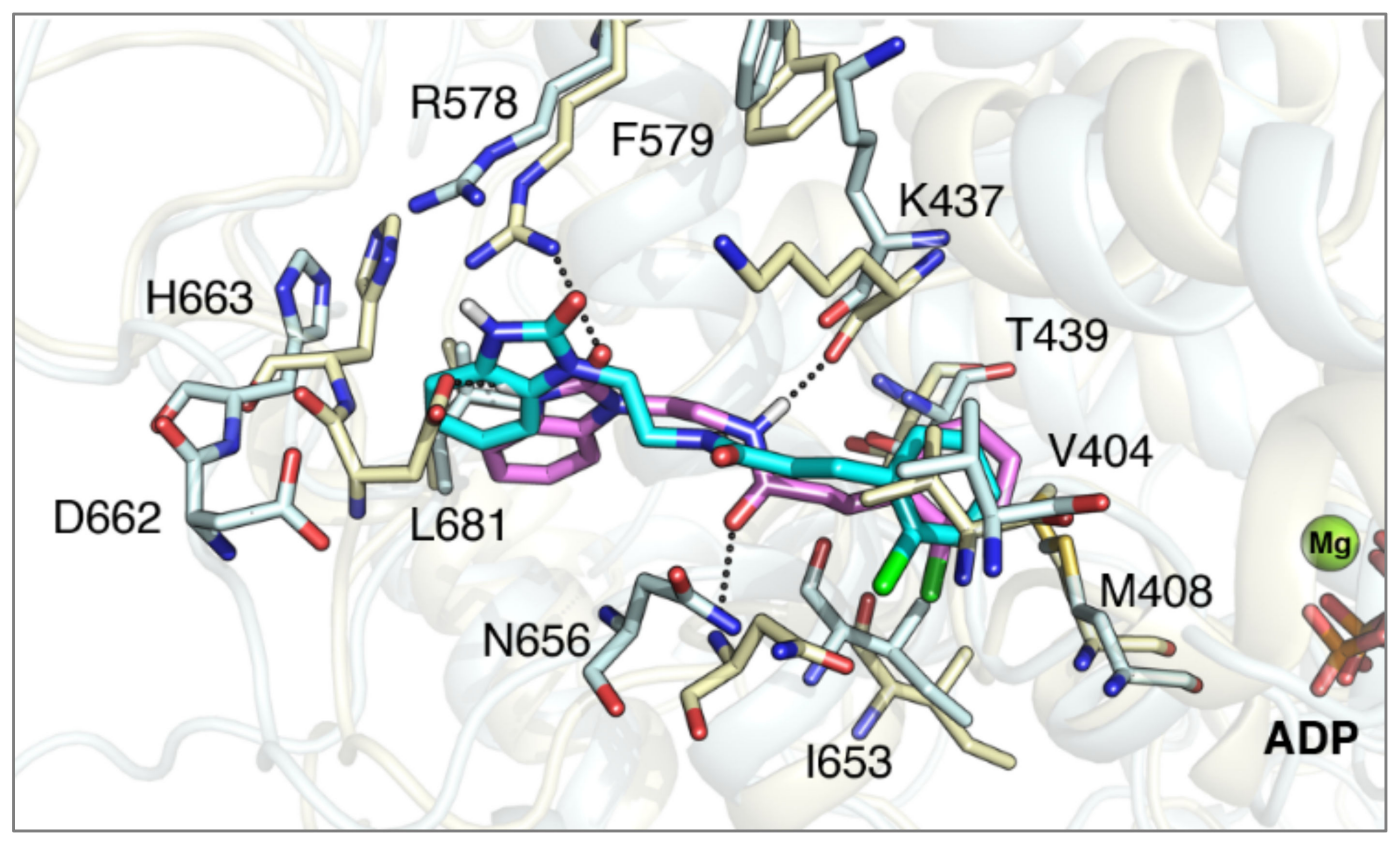
Compound **9** (INF120) in Med3 before and after MD simulation. Alignment of **9** (INF120) docking pose (pink) and **9** pose in the last frame of the MD simulation (cyan). Protein (docking: light yellow; post MD: light cyan) is represented as cartoon, residue side chains, ADP molecule, and **9** (pink) are shown as sticks, Mg ion is depicted as a green sphere. Residues, ADP and Mg ions are labelled. Hydrogen bonds are reported as dashed black lines. For clarity, only polar hydrogens of **9** are shown.

Compound **9** docking pose in Med3 (Figure 8) was chosen for 100 ns MD in NLRP3, for checking whether the pose was stable inside the p16 pocket. The RMSD plot (Figure S12) shows a slight rearrangement in the binding pose, which, however, was conserved throughout the MD simulation.

## 3. Materials and Methods

### 3.1. Chemistry

All the reactions were monitored by Thin Layer chromatography (TLC) on Merck 60 F254 (0.25 mm) plates, which were visualised by UV inspection (254 nm) and/or by spraying KMnO_4_ (0.5 g in 100 mL 0.1 N NaOH). Na_2_SO_4_ was used as drying agent for the organic phases. Flash chromatography (FC) purifications were performed using silica gel Merck with 60 mesh particles. Unless otherwise specified, all reagents were used as received without further purification. Dichloromethane was dried over P_2_O_5_ and freshly distilled under nitrogen prior to use. DMF was stored over 3 Å molecular sieves. Anhydrous THF was freshly distilled under nitrogen from Na/benzophenone ketyl. ^1^H and ^13^C-NMR spectra were registered on JEOL ECZR600 spectrometer, at 600 and 151 MHz. Coupling constants (J) are given in Hertz (Hz) and chemical shifts (δ) are given in ppm, calibrated to solvent signal as internal standard. Following abbreviations are used to describe multiplicities: s= singlet, d = doublet, t = triplet, q = quadruplet, m = multiplet and br= broad signal. The following abbreviations are used to identify exact proton: ArH = Aromatic proton, BzImH= benzimidazolone ring, Pip = piperidine. ESI-mass spectra were recorded on a Waters Micromass Quattro Micro equipped with an ESI source. Melting points were measured with a capillary apparatus (Büchi 540). The purity of the final compounds was determined by RP-HPLC. Analyses were performed with a HP1100 chromatograph system (Agilent Technologies, Palo Alto, CA, USA) equipped with a quaternary pump (G1311A), a membrane degasser (G1379A) and a diode-array detector (DAD) (G1315B) integrated into the HP1100 system. Data analyses were processed using a HP ChemStation system (Agilent Technologies). The analytical column was a LiChrospher^®^ 100 C18-e (250 × 4.6 mm, 5 µm) (Merck KGaA, 64271 Darmstadt, Germany) eluted with CH_3_CN 0.1% TFA/H_2_O 0.1% TFA in a ratio that depended on the characteristics of the compound. All compounds were dissolved in the mobile phase at a concentration of about 0.01 mg/mL and injected through a 20 μL loop. HPLC retention times (t_R_) were obtained at flow rates of either 1.0 or 1.2 mL/min and the column effluent was monitored using the DAD. The DAD acquired the UV spectra in the range from 190 to 800 nm, and the HPLC chromatogram was recorded at 226, 254, 580 and 660 nm (with 800 nm as the reference wavelength). The purity of the test samples was evaluated as the percentage ratio between the areas of the main peak and of possible impurities at the three wavelengths, and also using a DAD purity analysis of the chromatographic peak. The purity of all the target compounds was found to be ≥ 95%.

### 3.2. Synthesis of Target Compounds

#### 3.2.1. *Tert-butyl 3-(2-chlorophenyl)-2-(diethoxyphosphoryl)propanoate* (**24**) 

The reaction was conducted in nitrogen atmosphere. Sodium hydride (60%, 2.13 g, 21.9 eq) was added to a stirred solution of tert-butyl diethylphosphonoacetate (6.17 mL, 26.3 eq) in DMF (40 mL) at 0 °C. The reaction mixture was stirred 2 h at room temperature. p-chlorobenzilbromide (2.84 mL, 21.9 eq) was added dropwise at 0 °C, and the solution was stirred 2 h at room temperature. The reaction mixture was cooled to 0° and water was added (20 mL). The solvent was reduced under reduced pressure. The residue was dissolved in diethyl ether and washed with water (2 × 10 mL), brine (15 mL), dried (Na_2_SO_4_) and concentrated under reduced pressure to give **24** (8.20 g, 99.7%) as a white solid.

#### 3.2.2. *Tert-butyl 2-(2-chlorobenzyl)acrylate* (**25**) 

K_2_CO_3_ was dissolved in water (80 mL) and added to a stirred solution of **24** (8.25 g, 21.9 eq) and paraformaldehyde (5.25 mL, 175 eq) in water (80 mL). The reaction mixture was heated under reflux for 4 days. The mixture was cooled to room temperature and extracted with EtOAc (3 × 40 mL). The combined organic phases were washed with brine (15 mL), dried (Na_2_SO_4_), and concentrated under reduced pressure. The crude product was purified by silica gel chromatography (PE/EtOAc 95:5) to give 65 (4.98 g, 90.1%) as a colourless oil. Rf = 0.29 (PE/EtOAc 95:5); MS (ESI): *m/z* 275/277 [MNa^+^]; ^1^H NMR (300 MHz, CDCl_3_): δ = 7.36–7.15 (m, 4H, ArH), 6.17 (s, 1H, C=CHH), 5.25 (m, 1H, C=CHH), 3.71 (s, 2H, CH_2_), 1.45 (s, 9H, CH_3_); ^13^C NMR: (151 MHz, CDCl3): δ = 166.13, 139.88, 136.94, 134.47, 130.92, 129.59, 127.85, 126.83, 125.96, 80.91, 35.47, 28.05.

#### 3.2.3. *1-(1-(2-(2-chlorobenzyl)acryloyl)piperidin-4-yl)-1,3-dihydro-2H-benzo[d]imidazol-2-one* (**1**)

Compound **25** (0.960 g, 3.80 eq) was dissolved in a stirred solution of TFA/DCM (10%, 11.0 mL) at room temperature. After 12 h, the mixture was concentrated under reduced pressure. Crude product from the previous step (0.241 g, 1.23 eq) was dissolved in a stirred solution of **26** (0.399 g, 1.84 eq), DIPEA (0.313 mL, 1.84 eq), HOBt (0.025 g, 0.184 eq) and HBTU (0.813 g, 1.84 eq) in DMF (9 mL) at room temperature, and the mixture was stirred overnight. The solvent was evaporated under reduced pressure and a solution of NaHCO_3_ 10% (15 mL) was added. The mixture was extracted with EtOAc (3 × 10 mL). The combined organic phases were washed with brine (15 mL), dried (Na_2_SO_4_), and concentrated under reduced pressure. The crude product was purified by silica gel chromatography (DCM/MeOH 98:2) to give 20 (0.223 g, 45.7%) as a white solid. MS (ESI): *m/z* 396/398 [M + H]^+^; ^1^H NMR (300 MHz, CDCl_3_): δ = 10.00 (s, 1H, NH), 7.37–7.02 (m, 4H, ArH), 6.84–6.88 (m, 1H, C=CHH), 5.22–5.15 (m, 1H, C=CHH), 4.91–4.72 (m, 1H, NCHH), 4.60–4.37 (m, 1H, CH), 4.24–4.02 (m, 1H, NC’HH), 3.81 (d, J = 20.8, 2H, ArCH_2_), 2.83–2.58 (m, 2H, NCH_2_), 2.29–2.02 (m, 2H, CHCH2), 1.95–1.57 (m, 2H, CHCH_2_); ^13^C NMR: (151 MHz, CDCl_3_): δ = 170.54, 155.50, 141.85, 135.61, 134.94, 132.32, 130.12, 129.05, 128,90, 128.53, 127.51, 121.86, 121.38, 116.47, 110.36, 109.88, 50.88, 47.01, 41.62, 38.91, 30.30, 29.35.

#### 3.2.4. General Procedure for the Preparation of Compounds **2**–**4**

The appropriate chlorophenyl carboxylic acid (1 eq) was added to a stirred solution of CDI (1 eq) in DCM (10 mL) at room temperature. After 30 min, 1-(piperidin-4-yl)-2,3-dihydro-1,3-benzodiazol-2-one (1 eq) was added, and the mixture was stirred overnight. The mixture was washed with water (3 × 10 mL), brine (10 mL), dried (Na_2_SO_4_) and concentrated under reduced pressure.

*1-(1-(3-(2-chlorophenyl)propanoyl)piperidin-4-yl)-1,3-dihydro-2H-benzo[d]imidazol-2-one* (**2**):

The reaction was run with **29** (0.500 g, 2.71 mmol), CDI (0.439 g, 2.71 mmol) and **26** (0.588 g, 2.71 mmol) in DCM (10 mL). The crude product was purified by silica gel chromatography (DCM/MeOH 95:5) to give **2** (0.820 g, 78.8%) as a white solid. Rf = 0.54 (DCM/MeOH 9:1); mp: 97.3–101.2 °C; MS (ESI): *m/z* 384/386 [M + H]^+^; ^1^H NMR (600 MHz, CDCl_3_): δ = 10.55 (s, 1H, NH), 7.36 (dd, J = 7.9, 1H, ArH_3_), 7.34 (dd, J = 7.6, 1H, ArH_6_), 7.24 (t, J = 7.4, 1H, ArH_4_), 7.19 (t, J = 7.7, 1H, ArH_5_), 7.18–7.04 (m, 4H, BzImH), 4.92 (d, J = 13.0, 1H, NCHH), 4.54 (tt, J = 12.4, 4.2, 1H, CH), 4.04 (d, J = 13.5, 1H, NC’HH), 3.16 (td, J = 7.9, 2.6, 2H, ArCH_2_), 3.12 (d, J = 12.3, 1H, NCHH), 2.82–2.64 (m, 2H, COCH_2_), 2.70 (m, 1H, NC’HH), 2.33–2.22 (m, 2H, CHCHH), 1.89 (t, J = 11.1, 2H, CHCHH).

*2-1-(1-(2-chlorobenzoyl)piperidin-4-yl)-1,3-dihydro-2H-benzo[d]imidazol-2-one* (**3**): The reaction was run with **27** (0.100 g, 0.626 mmol), CDI (0.101 g, 0.626 mmol) and **26** (0.140 g, 0.626 mmol) in DCM (5 mL). The crude product was purified by silica gel chromatography (DCM/MeOH 98:2) to give **3** (0.081 g, 36.4%) as a white solid. Rf = 0.21 (DCM/MeOH 95:5); mp: 153.6–158.8 °C; MS (ESI): *m/z* 356/358 [M + H]^+^; ^1^H NMR (600 MHz, CDCl_3_): δ = 10.48 (s, 1H, NH), 7.41 (d, J = 7.0, ArH3), 7.39 (d, J = 7.9, 1H, ArH6), 7.30 (t, J = 7.5, ArH_5_), 7.22 (t, J = 7.8, ArH_4_), 7.11–6.95 (m, 4H, BzImH), 4.90 (d, J = 13.3, 1H, NCHH), 4.56 (tt, J = 12.3, 4.2, 1H, CH), 4.05 (d, J = 13.5, NC’HH), 3.92 (m, 2H, CHCH_2_), 3.21 (t, J = 12.6 Hz, NCHH), 2.74 (t, J = 12.4, NC’HH), 2.32–2.12 (m, 2H, CHCH_2_), 1.85 (dd, J = 39.6, 12.4, 2H, COCH_2_). ^13^C NMR: (151 MHz, CDCl3): δ = 166.82, 154.97, 135.75, 135.64, 130.30, 130.28, 129.75, 128.73, 127.63, 127.31, 121.50, 121.23, 109.96, 109.15, 50.42, 46.40, 41.33, 29.57, 28.86.

*1-(1-(2-(2-chlorophenyl)acetyl)piperidin-4-yl)-1,3-dihydro-2H-benzo[d]imidazol-2-one* (**4**): The reaction was run with **28** (0.100 g, 0.586 mmol), CDI (0.095 g, 0.586 mmol) and **26** (0.127 g, 0.586 mmol) in DCM (5 mL). The crude product was purified by silica gel chromatography (DCM/MeOH 95:5) to give **4** (0.176 g, 81.6%) as a white solid. Rf = 0.56 (DCM/MeOH 9:1); mp: 200.3–201.1 °C; MS (ESI): *m/z* 370/372 [M + H]^+^; ^1^H NMR (600 MHz, CDCl_3_): δ = 10.17 (d, J = 5.6, 1H, NH), 7.49–7.27 (m, 4H, ArH), 7.18–7.00 (m, 4H, BzImH), 5.04 (t, J = 15.8, 1H, NCHH), 4.60 (m, CH), 3.61 (d, J = 8.8, NC’HH), 3.35–3.11 (m, 1H, NCHH), 2.93 (m, 1H, NC’HH), 2.64–2.22 (m, 2H, CHCHH), 2.06–1.70 (m, 2H, CHCHH). ^13^C NMR: (151 MHz, CDCl_3_): δ = 168.67, 155.05, 133.75, 133.23, 130.63, 129.56, 128.59, 128.48, 128.11, 127.03, 121.39, 120.97, 109.96, 109.18, 50.27, 45.62, 41.81, 29.38, 28.85.

*1-(1-(2-chlorobenzyl)piperidin-4-yl)-1,3-dihydro-2H-benzo[d]imidazol-2-one* (**5**): p-chlorobenzylbromide (0.473 g, 2.301 mmol) was added to a stirred solution of **26** (0.500 g, 2.301 mmol) and TEA (0.320 mL, 2.301 mmol) in acetonitrile (10 mL). The reaction mixture was kept at room temperature for 2 h. The mixture was concentrated under reduced pressure. Next, 10% Na_2_CO_3_ solution was added (20 mL), and the product was extracted with EtOAc (3 × 25 mL). The combined organic phases were washed with brine, dried (Na_2_SO_4_), filtered and concentrated under reduced pressure. The crude product was purified by silica gel chromatography (DCM/MeOH 9:1) to give **5** (0.737 g, 93.7%) as a white solid. ^1^H NMR (600 MHz, CDCl_3_): δ =7.37 (dd, J = 7.8, 1H, ArH_3_), 7.31 (dd, J = 7.6, 1H, ArH_6_), 7.25 (t, J = 7.4, 1H, ArH_4_), 7.20 (t, J = 7.7, 1H, ArH_5_), 7.18–7.04 (m, 4H, BzImH), 4.33 (d, J = 13.0, 2H, NCHH), 4.54 (tt, J = 12.4, 4.2, 1H, CH), 3.66 (s,2H, ArCH_2_), 2.91 (d, J = 12.3, 2H, NCHH),2.33–2.22 (m, 2H, CHCHH), 1.89 (t, J = 11.1, 2H, CHCHH).

*1-[3-(2-chlorophenyl)propanol]pyrrolidine-2,5-dione* (**29a**): DCC (0.335 g, 1.625 mmol) and *N*-hydroxysuccinimide (0.317 g, 2.76 mmol) were added to a stirred solution of 3-(2-chlorophenyl)propanoic acid (0.300 g, 1.625 mmol) in dry THF (7 mL) at 0 °C. The reaction mixture was kept at 0 °C for 10 min, then stirred at room temperature overnight. The mixture was filtered (3 times) to remove the white solid (DCU), diluted with water (15 mL) and extracted with EtOAc (3 × 15 mL). The combined organic phases were washed with brine (20 mL), dried (Na_2_SO_4_), filtered and concentrated under reduced pressure. The product was purified by silica gel chromatography (DCM) to give **29a** (0.325 g, 71.1%) as a white solid. Rf = 0.67 (DCM); ^1^H NMR (300 MHz, CDCl_3_) δ= 7.31–7.16 (m, ArH), 3.07 (m, 2H, COCH_2_), 2.08 (s, 2H, ArCH_2_), 2.72 (s, 4H, COCH_2_CH_2_CO).

#### 3.2.5. General Procedure for the Preparation of Compounds **32** and **33**

To a stirred solution of **29a** (1 mmol) in DMF (10 mL), the appropriate amine (1 mmol) and the appropriate base (2 mmol) were added at room temperature. When the reaction was complete (TLC), the mixture was concentrated under reduced pressure. Next, 10% Na_2_CO_3_ solution was added (20 mL), and the product was extracted with EtOAc (3 × 25 mL). The combined organic phases were washed with brine, dried (Na_2_SO_4_), filtered and concentrated under reduced pressure.

*Methyl 4-(3-(2-chlorophenyl)propanamido)butanoate* (**33**): The reaction was run with **29a** (0.500 g, 1.77 mmol), methyl 4-aminobutyrate hydrochloride (0.545 g, 3.54 mmol) and triethylamine (0.742 mL, 5.33 mmol) in DMF (6 mL). The crude product was purified by silica gel chromatography (DCM/MeOH 98:2) to give **33** (0.210 g, 41.7%) as a colourless oil. Rf = 0.21 (DCM/MeOH 98:2); MS (ESI): *m/z* 284/286 [M + H]^+^; ^1^H NMR (600 MHz, CDCl_3_): δ = 7.36–7.14 (m, 4H, ArH), 7, 5.31 (d, J = 0.9, 1H, NH), 3.68 (s, 3H, CH_3_), 3.27 (q, J = 6.4, 2H, NHCH2), 3.09 (t, J = 7.7, 2H, ArCH_2_), 2.50 (t, J = 7.6, 2H, NCOCH_2_), 2.30 (t, J = 7.1, 2H, OCOCH_2_), 1.79 (p, J = 7.0, 2H, CH_2_CH_2_CH_2_).

*Ethyl (3-(2-chlorophenyl)propanoyl)glycinate* (**32**): The reaction was run with **29a** (0.156 g, 0.843 mmol), glycine ethyl ester hydrochloride (0.118 g, 0.843 mmol) and triethylamine (0.118 mL, 0.843 mmol) in DMF (3 mL). The crude product was purified by silica gel chromatography (DCM/MeOH 98:2) to give **32** (0.185 g, 97%). Rf = 0.81 (DCM/MeOH 95:5). ^1^H NMR (600 MHz, CDCl_3_): δ = 7.31–7.16 (m, 4H, ArH), 4.09–4.16 (m, 4H), 2.95 (t, J = 7.6, 2H, ArCH_2_), 1.21 (t, J = 7.8, 3H).

#### 3.2.6. *(3-(2-chlorophenyl)propanoyl)glycine* (**34**)

Compound **33** (0.185, 0.700 mmol) was dissolved in a stirred solution of aqueous NaOH (2.5 N, 0.730 mL) in EtOH (3 mL). After 2 h, a solution of HCl was added (5 N, 1 mL) and the solvent was evaporated under reduced pressure. The residue was suspended in HCl 0.5 N (10 mL) and filtered to give **34** as a white solid (0.061 g, 36%). ^1^H NMR (600 MHz, CDCl_3_): δ = 7.61 (d, J = 7.2, 1H), 7.45–7.10 (m, 3H, ArH), 4.09 (s, 2H, NHCH_2_), 2.95 (t, J = 7.6, 2H, ArCH_2_), 2.22 (t, J = 7.6, NCOCH_2_); ^13^C NMR: (151 MHz, CDCl_3_): δ = 176.90, 173.21, 146.87, 133.12, 129.10, 128.31, 128.16, 127.03, 42.79, 35.81, 29.15.

#### 3.2.7. *4-(3-(2-chlorophenyl)propanamido)butanoic acid* (**35**)

Compound **33** (0.210, 0.740 mmol) was dissolved in a stirred solution of aqueous NaOH (2.5 N, 0.770 mL) in MeOH (15 mL). After 12 h, the mixture’s pH was brought to 2 with a solution of HCl (5 N), and the methanol was evaporated under reduced pressure. The acidic aqueous phase was extracted with DCM (4 × 15 mL). The combined organic phases were washed with brine (10 mL), dried (Na_2_SO_4_) and concentrated under reduced pressure. The crude product was purified by silica gel chromatography (DCM/MeOH 95:5) to give **35** (0.149 g, 74.5%) as a white solid. Rf = 0.15 (DCM/MeOH 95:5); MS (ESI): *m/z* 268/270 [M + H]^+^; ^1^H NMR (600 MHz, CDCl_3_): δ = 7.45–6.97 (m, 4H, ArH), 4.87 (s, 1H, NH), 3.09 (t, J = 6.8, 2H, NHCH_2_), 2.95 (t, J = 7.6, 2H, ArCH_2_), 2.41 (t, J = 7.6, NCOCH_2_), 2.04 (t, J = 7.3, 2H, OCOCH_2_), 1.62 (p, J = 7.1, CH_2_CH_2_CH_2_); ^13^C NMR: (151 MHz, CDCl_3_): δ = 175.90, 173.80, 138.57, 133.85, 130.76, 129.53, 128.06, 127.19, 38.71, 35.86, 31.15, 29.65, 24.75.

#### 3.2.8. General Procedure for the Preparation of Compounds **36** and **6**–**8**

To a stirred solution of the appropriate carboxylic acid (1 mmol) in DMF (2 mL), DIPEA (2 mmol), HOBt (0.15 mmol), HBTU (1.5 mmol) and the appropriate amine (1.1 mmol) were added at room temperature and the mixture was stirred overnight. The solvent was evaporated under reduced pressure and a solution of NaHCO_3_ 10% (20 mL) was added. The mixture was extracted with EtOAc (3 × 20 mL). The combined organic phases were washed with brine (15 mL), dried (Na_2_SO_4_) and concentrated under reduced pressure.

*Tert-butyl N-{3-oxo-3-[4-(2-oxo-2,3-dihydro-1,3 benzodiazol-1-yl)piperidin 1 yl]propyl} carbamate* (**36**): The reaction was run using Boc-β-alanine (0.287 g, 1.52 mmol), DIPEA (0.70 mL, 4.14 mmol), HOBt (0.028 g, 0.21 mmol), HBTU (0.915 g, 2.07 mmol) and 1-(piperidin-4-yl)-2,3-dihydro-1,3-benzodiazol-2-one (0.300 g, 1.38 mmol) in DMF (5 mL). The crude product was purified by silica gel chromatography (PE/EtOAc/MeOH, 7:2.5:0.5) to give **36** (0.330 g, 61.5%) as a white foam. Rf = 0.22 (petroleum ether/EtOAc/MeOH 7:2:1); MS (ESI): *m/z* 389/391 [M + H]^+^; ^1^H NMR (600 MHz, CDCl_3_): δ = 10.12 (s, 1H, BzImNH), 7.16–7.01 (m, 4H, ArH), 4.85 (d, J = 12.5, 1H, PipNCHH), 4.53 (tt, J = 12.4, 4.1, 1H, CH), 4.01 (q, J = 12.7, 1H PipNC’HH), 3.47 (t, J = 5.2, 2H, NHCH_2_), 3.18 (t, J = 12.5, 1H, PipNCHH), 2.69 (t, J = 12.6, 1H, PipNC’HH), 2.60 (dt, J = 10.9, 5.6, 2H, COCH_2_), 2.48–2.21 (m, 2H, CHCH_2_), 1.90 (m, 2H, CHCH_2_), 1.43 (s, 9H, CH_3_); ^13^C NMR (151 MHz, CDCl_3_): δ = 170.20, 156.21, 155.04, 128.88, 128.11, 121.63, 121.35, 110.06, 109.32, 79.35, 50.72, 45.08, 41.45, 36.59, 33.59, 29.73, 28.99, 28.52.

*3-(2-chlorophenyl)-N-(2-oxo-2-(4-(2-oxo-2,3-dihydro-1H-benzo[d]imidazol-1-yl)piperidin-1-yl)ethyl)propenamide* (**6**): The reaction was run with **34** (0.061 g, 0.252 mmol), **26** (0.60 g, 0.277 mmol), HBTU (0.167 g, 0.378 mmol), HOBt (0.034 g, 0.025 mmol) and DIPEA (0.129 mL, 0.756 mmol) in DMF (3 mL). The crude product was purified by silica gel chromatography (DCM/MeOH, 95:5) to give **6** as a white solid (0.100 g, 89.8%). Rf = 0.23 (DCM/MeOH 95:5); MS (ESI): *m/z* 441/443 [M + H]^+^; ^1^H NMR (600 MHz, CDCl_3_): δ = 9.81 (s, 1H, BzImNH), 7.21–7.00 (m, 4H, ArH), 7.02–6.96, (m, 4H, BzImH), 4.85 (d, J = 12.5, PipNCHH), 4.47 (tt, J = 12.2, 4.0, CH), 4.21 (d, J = 8.1, NHCH_2_), 3.95 (d, J = 12.5, 1H, PipNC’HH), 3.13 (t, J = 12.0, 1H, PipNCHH), 3.07–3.03 (m, 2H, ArCH_2_), 2.65 (t, J = 12.0, 1H, PipNC’HH), 2.57–2.51 (m, 2H, CHCHH), 2.48 (t, J = 7.8, 2H, NHCOCH_2_), 2.38–2.23 (m, 2H, CHCHH), ^13^C NMR: (151 MHz, CDCl_3_): δ = 172.37, 170.30, 155.80, 154.92, 138.50, 134.02, 130.81, 129.65, 128.99, 127.91, 127.03, 121.70, 121.43, 110.03, 109.25, 50.83, 45.15, 41.55, 40.73, 36.33, 29.80, 29.71, 29.00.

*3-(2-chlorophenyl)-N-{3-oxo-3-[4-(2-oxo-2,3-dihydro-1,3-benzodiazol-1-yl)piperidin-1-yl] propyl}propanamide* (**7**): The reaction was run with **29** (0.093 g, 0.506 mmol), **37** (0.224 g, 0.557 mmol), HBTU (0.336 g, 0.759 mmol), HOBt (0.007 g, 0.051 mmol) and DIPEA (0.258 mL, 1,52 mmol) in DMF (5 mL) at room temperature. The crude product was purified by silica gel chromatography (DCM/MeOH, 95:5) to give **7** as a white solid (0.142 g, 58.2%). Rf = 0.43 (DCM/MeOH 9:1); mp: 114.7–116.2 °C; MS (ESI): *m/z* 455/457 [M + H]^+^; ^1^H NMR (600 MHz, CDCl_3_): δ = 9.85 (s, 1H, BzImNH), 7.25–7.21 (m, 1H, ArH_3_), 7.18–7.16 (m, 1H, ArH_6_), 7.08–7.05 (m, 2H, ArH_4-5_), 7.07–6.93, (s, 4H, BzImH), 4.82 (d, J = 12.6, 1H, PipNCHH), 4.50 (tt, J = 12.3, 4.1, 1H, CH), 3.97 (d, J = 12.5, 1H, PipNC’HH), 3.65–3.49 (m, 2H, NHCH_2_), 3.16 (t, J = 12.1, 1H, PipNCHH), 3.08 (t, J = 7.8, 2H, ArCH_2_), 2.69 (t, J = 12.2, 1H, PipNC’HH), 2.58 (dd, J = 9.7, 4.9, 2H, CHCHH), 2.52 (t, J = 7.7, 2H, NHCOCH_2_), 2.41–2.24 (m, 2H, CHCHH), 1.90 (t, J = 10.8, 2H, NCOCH_2_); _13_C NMR: (151 MHz, CDCl_3_): δ = 172.32, 170.26, 155.75, 154.88, 138.44, 133.98, 130.74, 129.58, 128.94, 127.83, 126.95, 121.65, 121.38, 109.98, 109.15, 50.72, 45.05, 41.51, 36.26, 35.23, 33.03, 29.73, 29.64, 28.96.

*3-(2-chlorophenyl)-N-(4-oxo-4-(4-(2-oxo-2,3-dihydro-1H-benzo[d]imidazol-1-yl)piperidin-1-yl)butyl)propenamide* (**8**): The reaction was run with **35** (0.149 g, 0.551 mmol), **26** (0.132 g, 0.606 mmol), HBTU (0.366 g, 0.824 mmol), HOBt (0.011 g, 0.083 mmol) and DIPEA (0.280 mL, 1.65 mmol) in DMF (5 mL). The crude product was purified by silica gel chromatography (DCM/MeOH 95:5) to give as a rose foam. The product was recrystalised from iPrOH (2 mL) by adding dropwise (iPr)_2_O to obtain **8** (0.149 g, 74.5%) as a white solid. Rf = 0.51 (DCM/MeOH 9:1); MS (ESI): *m/z* 467/469 [M-H]^−^; ^1^H NMR (600 MHz, CDCl_3_): δ = 9.95 (s, 1H, BzImNH), 7.43–6.90 (m, 8H, ArH), 4.84 (m, 1H, PipNCHH), 4.49 (t, J = 12.3, CH), 4.01 (m, 1H, PipNC’HH), 3.32 (t, J = 5.8,1H, PipNCHH), 3.18 (m, 2H, NHCH_2_), 3.09 (t, J = 7.5, 1H, PipNCHH), 2.68 (m, 2H, ArCH_2_), 2.56 (d, J = 5.9, 2H, CHCHH), 2.44 (m, 2H, NHCOCH_2_), 2.33 (m, 2H, NCOCH_2_), 1.88 (m, 2H, CHCHH), 1.21 (m, 2H, CCH_2_C); ^13^C NMR: (151 MHz, CDCl_3_): δ = 174.82, 173.25, 156.27, 139.64, 134.93, 131.79, 130.57, 130.53, 129.66, 129.11, 128.22, 122.64, 122.39, 110.58, 52.18, 46.39, 42.65, 39.92, 36.89, 31.26, 30.69, 30.66, 29.95, 26.18.

*1-[1-(3-aminopropanoyl)piperidin-4-yl]-2,3-dihydro-1,3-benzodiazol-2-onetrifluoroacetate salt* (**37**): Compound **36** (0.330 g, 0.849 mmol) was added to a stirred solution of trifluoracetic acid (0.5 mL, 2,94 mmol) in DCM (5 mL). The reaction mixture was kept at r.t. for 6 h. The solvent was evaporated under reduced pressure and washed 5 times with DCM to strip all the trifluoracetic acid. The residue was dried under reduced pressure to give **37** (0.342 g, 100%) as a white foam. The product was very hygroscopic and, within a few minutes, it became a sticky yellow semisolid. Rf = 0.23 (DCM/MeOH 9:1).

#### 3.2.9. General Procedure for the Preparation of Compounds **40** and **41**

p-fluoronitrobenezene (1 mmol) was dissolved in a stirred solution of N-Boc alchildiamine (1.1 mmol) and a suspension of fine-powdered K_2_CO_3_ (2 mmol) in dry DMF under nitrogen gas. The reaction mixture was kept at 70 °C overnight. The mixture was concentrated under reduced pressure. Brine (20 mL) was added and the product was extracted with EtOAc. The combined organic phases were washed with brine (15 mL), dried (Na_2_SO_4_) and concentrated under reduced pressure.

*Tert-butyl (3-((2-nitrophenyl)amino)propyl)carbamate* (**40**): The reaction was run with p-fluoronitrobenezene (0.310 mL, 3.04 mmol), **38** (0.582 g, 3.34 mmol) and K_2_CO_3_ (0.923 g, 6.68 mmol) in dry DMF under nitrogen gas. The crude product was purified by silica gel chromatography (PE/EtOAc 8:2) to give **40** (0.752 g, 94%) as an orange solid. Rf = 0.22 (PE/EtOAc 8:2); MS (ESI): *m/z* 496/498 [M + H]^+^; ^1^H NMR (300 MHz, CDCl_3_): δ = 8.15 (d, J = 1.4, 1H, ArH3), 8.05 (s, 1H, ArNH), 7.43 (t, J = 11.4, 1H, ArH_5_), 6.86 (d, J = 8.6, 1H, ArH_6_), 6.64 (t, J = 7.8, 1H, ArH_4_), 4.75 (s, 1H, CONH), 3.30 (dd, J = 12.8, 6.4, 2H, ArNHCH_2_), 3.20 (q, J = 6.6, 2H, CONHCH_2_), 1.92 (p, J = 6.8, 2H, CCH_2_C), 1.44 (s, 9H, CH_3_).

*Tert-butyl (3-((2-nitrophenyl)amino)ethyl)carbamate* (**41**): The reaction was run with p-fluoronitrobenezene (0.341 mL, 3.24 mmol), **39** (0.675 g, 4.21 mmol) and K_2_CO_3_ (1.12 g, 8.10 mmol) in dry DMF under nitrogen gas. The crude product was purified by silica gel chromatography (PE/EtOAc 9:1) to give **41** (0.740 g, 81.1%) as an orange solid. Rf = 0.19 (PE/EtOAc 8:2); ^1^H NMR (300 MHz, CDCl_3_): δ = 8.18 (d, J = 1.5, 1H, ArH_3_), 8.05 (s, 1H, ArNH), 7.45 (t, J = 8.6, 1H, ArH_5_), 6.94 (d, J = 8.6, 1H, ArH_6_), 6.68 (t, J = 7.8, 1H, ArH_4_), 4.82 (s, 1H, CONH), 3.59–3.33 (m, 4H, NCH_2_), 1.42 (m, 11H, CCH_2_C, CH_3_).

#### 3.2.10. General Procedure for the Preparation of Compounds **42** and **43**

The appropriate p-nitroaniline derivatives (1 mmol) were dissolved in a stirred suspension of Pd/C (10%, 0.1 mmol) in the reaction solvent. The reaction mixture was kept overnight under hydrogen atmosphere (1 atm). The suspension was filtered and concentrated under reduced pressure.

*Tert-butyl (3-((2-aminophenyl)amino)propyl)carbamate* (**42**): The reaction was run with **40** (0.727 g, 2.46 mmol) and Pd/C (10%, 0.073 g) in MeOH (10 mL) under atmospheric H_2_. The crude product was purified by silica gel chromatography (PE/EtOAc 7:3) to give **42** (0.553 g, 85.0%) as a violet solid. Rf = 0.24 (PE/EtOAc 1:1); ^1^H NMR (300 MHz, CDCl_3_): δ = 6.90–6.60 (m, 4H, ArH), 4.71 (s, 1H, ArNHC), 3.41 (s, 2H, ArNH_2_), 3.30 (dd, J = 12.8, 6.4, 2H, CONHCH_2_), 3.20 (t, J = 6.6, 2H, ArNHCH_2_), 1.85 (p, J = 6.6, 2H, CCH_2_C), 1.47 (s, 9H, CH_3_); ^13^C NMR: (75 MHz, CDCl_3_): δ = 156.56, 137.85, 134.82, 120.97, 119.11, 116.89, 112.30, 79.75, 41.87, 38.78, 30.16, 28.82.

*Tert-butyl (3-((2-aminophenyl)amino)ethyl)carbamate* (**43**): the reaction was run with **41** (0.740 g, 2.63 mmol) and Pd/C (10%, 0.073 g) in dry THF (10 mL) under atmospheric H_2_. The crude product was purified by silica gel chromatography (PE/EtOAc 7:3) to give **43** (0.560 g, 85.0%) as a violet solid. Rf = 0.29 (PE/EtOAc 1:1).

#### 3.2.11. General Procedure for the Preparation of Compounds **44** and **45**

The appropriate p-diaminobenzene (1 mmol) derivatives were added to a stirred solution of CDI (1.1 mmol) in THF. The reaction was kept at room temperature overnight. The reaction mixture was concentrated under reduced pressure. The residue was dissolved in DCM and washed with water (2 × 15 mL), with brine (15 mL), dried (Na_2_SO_4_) and concentrated under reduced pressure.

*Tert-butyl (3-(2-oxo-2,3-dihydro-1H-benzo[d]imidazol-1-yl)propyl) carbamate* (**44**): The reaction was run with **42** (0.538 g, 2.03 mmol) and CDI (0.365 g, 2.25 mmol) in THF (45 mL). The crude product was purified by silica gel chromatography (DCM/MeOH 98:2 and PE/EtOAc 6:4) to give **44** (0.385 g, 65.3%) as a dark yellow solid. Rf = 0.12 (PE/EtOAc 1:1); MS (ESI): *m/z* 292 [M + H]^+^; ^1^H NMR (300 MHz, CDCl_3_): δ = 10.55 (s, 1H, BzImNH), 7.22–6.95 (m, 4H, ArH), 5.57 (s, 1H, NH), 4.01 (t, J = 6.4, 2H NCH_2_), 3.16 (dd, J = 12.3, 6.2, 2H, NHCH_2_), 1.94 (p, J = 6.4, 2H, CCH_2_C), 1.47 (s, 9H, CH_3_); ^13^C NMR: (75 MHz, CDCl_3_): δ = 156.61, 156.61, 130.28, 128.55, 122.16, 121.86, 110.29, 108.29, 79.56, 38.14, 37.39, 28.85, 28.58.

*Tert-butyl (3-(2-oxo-2,3-dihydro-1H-benzo[d]imidazol-1-yl)ethyl)carbamate* (**45**): The reaction was run with **43** (0.568 g, 2.23 mmol) and CDI (0.361 g, 2.23 mmol) in THF (10 mL). The crude product was purified by silica gel chromatography (PE/EtOAc 6:4 to 5:4.5:0.5) to give **45** (0.292 g, 47.3%) as a white solid. Rf = 0.58 (DCM/MeOH 9:1); MS (ESI): *m/z* 278 [MH+]; ^1^H NMR (300 MHz, CDCl_3_): δ = 9.59 (s, 1H, BzImNH), 7.43–6.90 (m, 4H, ArH), 4.11 (q, J = 7.1, 2H, NCH_2_), 3.09 (t, J = 7.5, 2H, NHCH_2_), 1.21 (s, 9H, CH_3_); ^13^C NMR: (75 MHz, CDCl_3_): δ = 156.17, 155.76, 130.70, 127.91, 121.74, 121.63, 109.57, 108.21, 79.60, 40.56, 39.70, 28.52.

#### 3.2.12. *1-(3-aminopropyl)-1,3-dihydro-2H-benzo[d]imidazol-2-one* (**46**)

Compound **44** (0.292 g, 1.05 mmol) was dissolved in a stirred solution of TFA/DCM (10%, 5.5 mL) at room temperature. After 4 h, the reaction mixture was concentrated under reduced pressure to give **46** (0.191 g, 62.5%) as a white foam. MS (ESI): *m/z* 192 [M + H]^+^; ^1^H NMR (300 MHz, CDCl_3_): δ = 7.12–7.02 (m, 4H, ArH), 3.85 (t, J = 6.7, NCH_2_), 2.90 (m, 2H, NH_2_CH_2_), 1.99 (m, 2H, CCH_2_C).

#### 3.2.13. *1-(3-aminoethyl)-1,3-dihydro-2H-benzo[d]imidazole-2-one* (**47**)

Compound **45** (0.337 g, 1.16 mmol) was dissolved in a stirred solution of TFA/DCM (10%, 8.8 mL) at room temperature. After 3 h, the reaction mixture was concentrated under reduced pressure to give **47** (0.191 g, 62.5%) as a white-yellow foam. ^1^H NMR (600 MHz, MeOD): δ = 7.16–7.05 (m, 4H, ArH), 4.17 (t, J = 5.9, 2H, NCH_2_), 3.30 (dd, J = 11.0, 5.0, 2H, NH_2_CH_2_). 13C NMR: (75 MHz, MeOD): δ = ^13^C NMR (151 MHz, MeOD) δ 155.87, 129.76, 128.57, 121.96, 121.38, 109.44, 107.52, 38.41, 38.19.

#### 3.2.14. General Procedure for the Preparation of Compounds **32** and **33**

To a stirred solution of **29a** (1 mmol) in DMF (10 mL), the appropriate amine (1 mmol) and the appropriate base (2 mmol) were added at room temperature. When the reaction was complete (TLC), the mixture was concentrated under reduced pressure. Na_2_CO_3_ 10% solution was added (20 mL), and the product was extracted with EtOAc (3 × 25 mL). The combined organic phases were washed with brine, dried (Na_2_SO_4_), filtered and concentrated under reduced pressure.

*3-(2-chlorophenyl)-N-(2-(2-oxo-2,3-dihydro-1H-benzo[d]imidazol-1-yl)ethyl) propanamide* (**9**): The reaction was run with **29a** (0.100 g, 0.354 mmol), **47** (0.097 g, 0.354 mmol) and DIPEA (0.088 mL, 0.532 mmol) in DMF (3 mL). The crude product was purified by silica gel chromatography (DCM/MeOH 95:5) to give **9** (0.118 g, 86.4%) as a white solid. Rf = 0.21 (DCM/MeOH 95:5); MS (ESI): *m/z* 366/368 [M+Na]^+^; ^1^H NMR (300 MHz, CDCl_3_): δ = 10.75 (s, 1H, BzImNH), 7.97 (t, J = 5.9, 1H, ArH), 7.35–6.83 (m, 7H, ArH), 3.99 (s, 1H, NH), 3.71 (t, J = 6.1, 2H, NCH_2_), 3.22–3.16 (m, 2H, NHCH_2_), 2.72 (t, J = 7.2, 2H, ArCH_2_), 2.17 (t, J = 7.0, 2H); ^13^C NMR: (75 MHz, CDCl_3_): δ = 172.17, 155.09, 139.40, 133.68, 131.42, 131.30, 130.03, 129.11, 128.82, 128.14, 121.56, 121.29, 109.56, 108.32, 40.31, 38.10, 35.79, 29.44.

*3-(2-chlorophenyl)-N-(3-(2-oxo-2,3-dihydro-1H-benzo[d]imidazol-1-yl)propyl) propanamide* (**10**): The reaction was run with **29a** (0.130 g, 0.458 mmol), **46** (0.140 g, 0.458 mmol) and DIPEA (0.116 mL, 0.687 mmol) in DMF (5 mL). The crude product was purified by silica gel chromatography (DCM/MeOH 97:3) to give **10** (0.141 g, 85.9%) as a white solid. Rf = 0.27 (DCM/MeOH 98:2); MS (ESI): *m/z* 358/360 [MH+]; ^1^H NMR (300 MHz, CDCl_3_): δ = 10.40 (s, 1H, BzImNH), 7.26–6.90 (m, 8H, ArH), 4.73 (s, 1H, NH), 3.73 (t, J = 5.9, NHCH_2_), 3.13 (m, 2H, NCH_2_), 3.05 (m, 2H, COCH_2_); 2.52 (t, J = 7.7, 2H, ArCH_2_), 1.79 (m, 2H, CCH_2_C); ^13^C NMR: (75 MHz, CDCl_3_): δ = 172.83, 156.50, 138.64, 134.30, 131.17, 130.09, 129.93, 128.42, 128.23, 127.32, 122.41, 122.07, 110.38, 108.34, 37.88, 36.69, 36.02, 30.24, 27.81.

#### 3.2.15. *Tert-butyl 2-oxo-2,3-dihydro-1H-benzo[d]imidazole-1-carboxylate* (**48a**) 

Benzimidazolone (2.5 g, 18.6 mmol) was dissolved in dry DMF (45 mL) under nitrogen atmosphere. NaH (60%, 0.818 g, 20.5 mmol) was added portionwise to the solution at 0 °C. (Boc)_2_O (0.406 g, 18.6 mmol) was dissolved in DMF (10 mL) and added dropwise in the reaction mixture and stirred at room temperature overnight. The mixture was concentrated under reduced pressure. Water (100 mL) was added, and the product was extracted with EtOAc (5 × 50 mL). The combined organic phases were washed with brine (15 mL), dried (Na_2_SO_4_) and concentrated under reduced pressure. The residue was purified by silica gel chromatography (PE/EtOAc 7:3) to give **48a** (3.74 g, 85.9%) as a white solid. Rf = 0.21 (PE/EtOAc 1:1); ^1^H NMR (600 MHz, CDCl_3_): δ = 10.45 (s, 1H, BzImNH), 7.25–6.85 (m, 4H, BzImH), 1.45 (s, 9H, CH_3_). ^13^C NMR: (75 MHz, CDCl^3^): δ = 158.85, 155.79, 130.14, 128.11, 122.13, 121.58, 110.09, 107.95, 82.89, 28.09.

#### 3.2.16. *Tert-butyl 3-(3-bromopropyl)-2-oxo-2,3-dihydro-1H-benzo[d]imidazole-1-carboxylate* (**48**)

Compound **48a** (1.00 g, 4.27 mmol) was added to a stirred solution of dibromopropane (4.35 mL, 42.7 mmol), fine-powdered K_2_CO_3_ (2.95 g, 21,35 mmol) and KI (0.070 g, 0.425 mmol) in ACN (20 mL). The reaction mixture was kept at room temperature overnight. The solvent was evaporated under reduced pressure, saturated NH_4_Cl (20 mL) was added and the product was extracted with DCM (6 × 20 mL). The combined organic phases were washed with brine (15 mL), dried (Na_2_SO_4_) and concentrated under reduced pressure. The residue was purified by silica gel chromatography (PE/EtOAc 9:1) to give **48** (0.998 g, 66.2%) as a white solid. Rf = 0.57 (PE/EtOAc 7:3); ^1^H NMR (600 MHz, CDCl_3_): δ = 7.82 (d, J = 7.7, 1H, BzImH), 7.20 (t, J = 7.7, 1H, BzImH), 7.12 (d, J = 14.5, 1H, BzImH), 7.07 (d, J = 7.9, 1H, BzImH), 4.00 (t, J = 6.8, 2H, NCH_2_), 3.43 (t, J = 6.4, 2H, BrCH_2_), 2.31 (p, J = 6.5, 2H, CCH_2_C), 1.66 (s, 9H, CH_3_); ^13^C NMR: (151 MHz, CDCl_3_): δ = 151.14, 148.87, 129.44, 126.25, 124.14, 122.36, 114.67, 107.62, 84.91, 39.62, 31.03, 30.35, 28.19.

#### 3.2.17. *Tert-butyl 3-(3-bromopropyl)-2-oxo-2,3-dihydro-1H-benzo[d]imidazole-1-carboxylate* (**49**)

Compound **48** (1.00 g, 4.27 mmol) was added to a stirred solution of dibromopropane (4.35 mL, 42.7 mmol), fine-powdered K_2_CO_3_ (2.95 g, 21,35 mmol) and KI (0.070 g, 0.425 mmol) in ACN (20 mL). The reaction mixture was kept at room temperature overnight. The solvent was evaporated under reduced pressure, saturated NH_4_Cl (20 mL) was added and the product was extracted with DCM (6 × 20 mL). The combined organic phases were washed with brine (15 mL), dried (Na_2_SO_4_) and concentrated under reduced pressure. The residue was purified by silica gel chromatography (PE/EtOAc 9:1) to give **49** (0.998 g, 66.2%) as a white solid. Rf = 0.57 (PE/EtOAc 7:3); ^1^H NMR (600 MHz, CDCl_3_): δ = 7.82 (d, J = 7.7, 1H, BzImH), 7.20 (t, J = 55 7.7, 1H, BzImH), 7.12 (d, J = 14.5, 1H, BzImH), 7.07 (d, J = 7.9, 1H, BzImH), 4.00 (t, J = 6.8, 2H, NCH_2_), 3.43 (t, J = 6.4, 2H, BrCH_2_), 2.31 (p, J = 6.5, 2H, CCH_2_C), 1.66 (s, 9H, CH_3_); ^13^C NMR: (151 MHz, CDCl_3_): δ = 151.14, 148.87, 129.44, 126.25, 124.14, 122.36, 114.67, 107.62, 84.91, 39.62, 31.03, 30.35, 28.19.

#### 3.2.18. *Tert-butyl 3-(3-(3-(2-chlorophenyl)-N-methylpropanamido)propyl)-2-oxo-2,3-di-hydro-1H-benzo[d]imidazole-1-carboxylate* (**50**)

Compound **29** (0.056 g, 0.304 mmol) was dissolved in a solution of CDI (0.049 g, 0.304 mmol) in DCM (3 mL). After 30′, **49** (0.093 g, 0.304mmol) was added, and the mixture was stirred overnight at room temperature. The reaction mixture was washed with NaHCO_3_ 10% (3 × 15 mL), brine (50 mL), dried (Na_2_SO_4_) and concentrated under reduced pressure. The residue was purified by silica gel chromatography (DCM/MeOH 97:3) to give **50** (0.081 g, 56.1%) as a white solid. Rf = 0.39 (DCM/MeOH 95:5); ^1^H NMR (600 MHz, CDCl_3_): δ = 7.39–7.02 (m, 4H, ArH), 3.97 (t, J = 7.2, 2H, BzImNCH_2_), 3.53 (t, J = 7.2, 2H, NCH_2_), 3.09–3.02 (m, 2H, COCH_2_), 2.97 (s, 3H, CH_3_), 2.67 (dd, J = 14.5, 6.4, 2H, ArCH_2_), 2.09–1.96 (m, 2H, CCH_2_C), 1.66 (s, 9H, CH_3_).

#### 3.2.19. *3-(2-chlorophenyl)-N-methyl-N-(3-(2-oxo-2,3-dihydro-1H-benzo[d]imidazole-1-yl)propyl)propenamide* (**11**)

Compound **50** (0.080 g, 0.169 mmol) was dissolved in TFA (1 mL) and stirred 1 h at room temperature. TFA was evaporated under reduced pressure and the residue was dissolved in DCM. The solution was washed with saturated NaHCO_3_ (2 × 10 mL), dried (Na_2_SO_4_) and concentrated under reduced pressure. The residue was purified by silica gel chromatography (DCM/MeOH 95:5) to give **11** (0.035 g, 55.5%) as a white solid. Rf = 0.45 (DCM/MeOH 95:5); ^1^H NMR (600 MHz, CDCl_3_): δ = 9.97 (s, 1H, NH), 7.34–7.05 (m, 4H, ArH), 3.90 (t, J = 7.3, 2H, BzImNCH_2_), 3.48 (t, J = 7.3, 2H, NCH_2_), 3.07–3.01 (m, 2H, COCH_2_), 2.95 (s, 3H, CH_3_), 2.67 (dd, J = 14.5, 6.4, 2H, ArCH_2_), 2.07–1.92 (m, 2H, CCH_2_C); ^13^C NMR: (151 MHz, CDCl_3_): δ = 173.62, 160.55, 138.10, 133.87, 130.96, 129.63, 129.57, 129.50, 128.14, 127.43, 127.24, 127.19, 116.02, 108.64, 45.98, 38.94, 35.84, 33.27, 29.97, 29.63.

#### 3.2.20. *Tert-butyl (1-(3-(2-chlorophenyl)propanoyl)piperidin-4-yl)carbamate* (**51**)

A solution of thionyl chloride (1.20 mL, 16.3 mmol) in DCM (5 mL) was added dropwise over 15′ in a solution of **29** (2.0 g, 10.83 mmol) and DMF (0.100 mL) in dry DCM (33 mL) at 0 °C under nitrogen atmosphere. The reaction mixture was kept 2 h at room temperature under nitrogen atmosphere. The solvent was evaporated under reduced pressure. The residue was dissolved in a solution of DIPEA (3.70 mL, 21.7 mmol) in DCM (30 mL), and a solution of tert-butyl piperidin-4-ylcarbamate (2.39 g, 11.9 mmol) was added dropwise at 0 °C. The mixture was stirred for 3h at room temperature. The reaction mixture was washed with NaHCO_3_ 10% (3 × 15 mL), brine (50 mL), dried (Na_2_SO_4_) and concentrated under reduced pressure. The residue was purified by silica gel chromatography (PE/EtOAc 7:3 to PE/EtOAc/MeOH 5:3:2) to give **51** (3.42 g, 86.3%) as a white solid. MS (ESI): *m/z* 389/391 [M+Na]^+^; ^1^H NMR (600 MHz, CDCl_3_): δ = 7.31 (dd, J = 7.8, 1.5, 1H, ArH_3_), 7.27–7.21 (m, 1H, ArH_6_), 7.14 (dtd, J = 18.8, 7.4, 1.7, 2H, ArH_4-5_), 4.48 (s, 1H, NH), 3.74 (d, J = 13.2, 1H, NCHH), 3.65–3.56 (m, 1H, CH), 3.04 (t, J = 7.9, 2H, ArCH_2_), 2.99–2.97 (m, 1H, NC’HH), 2.69 (t, J = 12.6, 2H, NCH_2_), 2.60 (td, J = 7.7, 3.7, 2H, OCCH_2_), 1.89 (d, J = 12.7, 2H, CHCH_2_), 1.41 (s, 9H, CH_3_), 1.26–1.08 (m, 2H, CHCH_2_); ^13^C NMR: (151 MHz, CDCl_3_): δ = 170.45, 155.15, 138.80, 133.94, 131.03, 129.60, 127.91, 127.08, 79.64, 47.90, 44.45, 33.05, 32.17, 29.84, 28.47.

#### 3.2.21. *Phenyl-N-(1-(3-(2-chlorophenyl)propanoyl)piperidin-4-yl)-N’-cyanocarbamimidate* (**52**)

Compound **51** (3.30 g, 8.99 mmol) was dissolved in a stirred solution of TFA (7.00 mL) in DCM (40 mL). The reaction mixture was kept at room temperature overnight. The mixture was concentrated under reduced pressure. The product was dissolved in HCl 1 N (40 mL) and washed with diethyl ether (2 × 25 mL). The aqueous phase was basified with NaOH pellets to pH 12 and extracted with DCM (5 × 20 mL). The combined organic phases were washed with brine, dried (Na_2_SO_4_), filtered and concentrated under reduced pressure. The product obtained (2.4 g, 8.99 mmol) was dissolved in a stirred solution of diphenyl cyanocarbonimidate (2.36 g, 9.89 mmol) in DCM (20 mL). After 1 h the solvent was evaporated under reduced pressure. The residue was purified by silica gel chromatography (petroleum ether/EtOAc/MeOH 6.5:3:0.5 to 6:3:1) to give **52** (3.40 g, 96.0%) as a white foam. R_f_ = 0.61 (DCM/MeOH 95:5); MS (ESI): *m/z* 425/427 [M + H]^+^; ^1^H NMR (600 MHz, CDCl_3_): δ = 7.35–7.14 (m, 9H, ArH), 6.34 (s, 1H, NH), 4.94 (d, J = 14.6, 1H, OCH_2_), 3.75 (s, 1H, NHCHH), 3.68–3.53 (m, 1H, CH), 3.02–2.97 (m, 1H, NHC’HH), 2.95 (d, J = 11.4, 1H, NHCHH), 2.89 (t, J = 7.8, 2H, ArCH_2_), 2.65 (t, J = 12.2, 1H, NHC’HH), 2.55 (dd, J = 15.8, 7.5, 2H, OCCH_2_), 1.80 (s, 2H, CHCH_2_), 1.16–0.96 (m, 1H, CHCH_2_); ^13^C NMR: (151 MHz, CDCl_3_): δ = 170.54, 170.33, 159.16, 140.94, 138.40, 136.13, 133.74, 130.79, 129.50, 129.11, 128.49, 128.37, 128.26, 127.87, 127.14, 126.97, 126.21, 118.10, 48.76, 45.79, 44.00, 40.28, 34.83, 32.85, 32.27, 31.50, 29.66.

#### 3.2.22. *1-(1-(3-(2-chlorophenyl)propanoyl)piperidin-4-yl)-2-cyano-3-methylguanidine* (**12**)

Compound **52** (0.600 g, 1.46 mmol) was dissolved in a solution of ethanolic ethylamine (33%, 2.72 mL, 29.2 mmol) and stirred 1.5 h at room temperature. The reaction mixture was cooled to 0 °C and filtered to isolate **12** (0.450 g, 88.6%) as a white solid. R_f_ = 0.35 (DCM/MeOH 95:5); mp: 208.6–210.0 °C; MS (ESI): *m/z* 348/350 [M + H]^+^; ^1^H NMR (600 MHz, CDCl_3_): δ = 7.29–7.24 (m, 1H, ArH_3_), 7.18 (dd, J = 7.3, 1.8, 1H, ArH_6_), 7.15–7.11 (m, 1H, ArH_5_), 7.09 (td, J = 7.6, 2.0, 1H, ArH_4_), 4.47 (d, J = 13.6, 1H, NCHH), 3.76 (d, J = 15.0, 1H, CH), 3.72 (s, 1H, NC’HH), 2.96 (dd, J = 8.0, 2.5, 2H, ArCH_2_), 2.94 (d, J = 9.8, 1H, NCHH), 2.74 (s, 3H, CH_3_), 2.58 (d, J = 8.1, 1H, NC’HH), 2.54–2.56 (m, 2H, OCCH_2_), 1.86 (dd, J = 28.1, 12.5, 2H, CHCH_2_), 1.22 (dqd, J = 64.3, 12.4, 4.2, 2H, CHCH_2_); ^13^C NMR: (151 MHz, CDCl_3_): δ = 171.10, 159.70, 138.29, 133.77, 130.77, 129.58, 128.02, 127.11, 119.00, 48.91, 44.65, 40.94, 33.03, 32.45, 31.44, 29.70, 28.23

#### 3.2.23. *1-benzyl-3-(1-(3-(2-chlorophenyl)propanoyl)piperidin-4-yl)-2-cyanoguanidine* (**13**)

Compound **52** (0.200 g, 0.487 mmol) was dissolved in a solution of benzylamine (0.265 mL, 2.43 mmol) in iPrOH (2 mL) and stirred for 24 h at room temperature. The solvent was evaporated under reduced pressure. The residue was dissolved in DCM (20 mL), washed with NaHCO_3_ 10% (3 × 15 mL), brine (50 mL), dried (Na_2_SO_4_) and concentrated under reduced pressure. The residue was purified by silica gel chromatography (DCM to DCM/MeOH 95:5) to give **13** (0.170 g, 81.9%) as a white solid; ^1^H NMR (600 MHz, CDCl_3_): δ = 7.35–7.14 (m, 9H, ArH), 6.34 (s, 2H, NHCH_2_Ar), 4.94 (d, J = 14.6, 1H, NCHH), 3.75 (s, 1H, CH), 3.68–3.53 (m, 1H, NC’HH), 2.95 (d, J = 11.4, 1H, NCHH), 2.89 (t, J = 7.8, 2H, ArCH_2_), 2.65 (t, J = 12.2, 1H, NC’HH), 2.55 (dd, J = 15.8, 7.5, 2H, OCCH_2_), 1.85–1.75 (m, 2H, CHCH_2_), 1.15–0.95 (m, 2H, CHCH_2_); ^13^C NMR: (151 MHz, CDCl_3_): δ = 170.54, 170.33, 159.16, 140.94, 138.40, 136.13, 133.74, 130.79, 129.50, 129.11, 128.49, 128.37, 128.26, 127.87, 127.14, 126.97, 126.21, 118.10, 48.76, 45.79, 44.00, 40.28, 34.83, 32.85, 32.27, 31.50, 29.66.

#### 3.2.24. *Tert-butyl 4-(1H-benzo[d]imidazol-1-yl)piperidine-1-carboxylate* (**54**)

Compound **53** (0.500 g, 1.79 mmol) was dissolved to a stirred suspension of benzimidazole (0.211 g, 1.79 mmol) and K_2_CO_3_ (0.49 g, 3.58 mmol) in DMF (2 mL). The mixture was heated under reflux for 2 days. The reaction mixture was concentrated under reduced pressure. The residue was dissolved in EtOAc and washed with water (2 × 15 mL), brine (15 mL), dried (Na_2_SO_4_) and concentrated under reduced pressure. The crude product was purified by silica gel chromatography (DCM/MeOH 95:5) to give **54** (0.101 g, 18.7%) as a yellow oil. R_f_ = 0.74 (DCM/MeOH 95:5).

#### 3.2.25. *1-(piperidin-4-yl)-1H-benzo[d]imidazole, trifluoroacetate salt* (**55**)

Compound **54** (0.081 g, 0.270 mmol) was dissolved in a stirred solution of TFA/DCM (10%, 4.4 mL) at room temperature. After 2 h, the reaction mixture was concentrated under reduced pressure to give **55** (0.084 g, 99.6%) as a white foam. Rf = 0.08 (DCM/MeOH 95:5 + NH_3_). MS (ESI): *m/z* 202 [M + H]^+^; ^1^H NMR (600 MHz, MeOD): δ = 9.58 (s, 1H, BzH_2_), 8.11–8.02 (m, 1H, BzH_4_), 7.91–7.83 (m, 1H, BzH_7_), 7.73–7.63 (m, 2H, BzH_5-6_), 3.73–3.59 (m, 2H, NCH_2_), 3.36–3.31 (m, 2H, CH), 2.61–2.49 (m, 2H, CHCH_2_), 2.47–2.37 (m, 2H, CHCH_2_); ^13^C NMR: (151 MHz, MeOD): δ = 139.26, 131.36, 130.62, 127.15, 126.66, 114.80, 112.97, 52.89, 42.89, 28.02.

#### 3.2.26. *1-(4-(1H-benzo[d]imidazol-1-yl)piperidin-1-yl)-3-(2-chlorophenyl)propan-1-one* (**14**)

Compound **55** (0.084g, 0.270 mmol) was added to a stirred solution of **29a** (0.076 g, 0.270 mmol) and DIPEA (0.138 mL, 0.811 mmol) in DMF (1 mL). The mixture was kept at room temperature overnight. The reaction mixture was concentrated under reduced pressure. Water (10 mL) was added, and the product was extracted with EtOAc (3 × 25 mL). The combined organic phases were washed with brine, dried (Na_2_SO_4_), filtered and concentrated under reduced pressure. The crude product was purified by silica gel chromatography (DCM/MeOH 97:3) to give **14** (0.083 g, 83.6%) as a colourless oil. Rf = 0.23 (DCM/MeOH 97:3). MS (ESI): *m/z* 368/370 [M + H]^+^; ^1^H NMR (600 MHz, CDCl_3_): δ = 7.96 (s, 1H, BzH_2_), 7.81–7.77 (m, 1H, ArH_3_), 7.39–7.14 (m, 7H, BzH, ArH), 4.92–4.86 (m, 1H, NCHH), 4.37 (tt, J = 12.0, 3.9, 1H, CH), 4.05–3.97 (m, 1H, NC’HH), 3.13 (m, 3H, NCHH, ArCH_2_), 2.81–2.60 (m, 3H, NC’HH, COCH_2_), 2.14 (dd, J = 31.0, 12.2, 2H, CHCH_2_), 1.88 (qd, J = 12.5, 4.4, CHCHH), 1.65 (qd, J = 12.5, 4.3, 1H, CHCHH); ^13^C NMR: (151 MHz, CDCl_3_): δ = 170.63, 143.35, 140.12, 138.59, 133.98, 132.97, 131.30, 129.72, 128.11, 127.11, 123.21, 122.71, 120.59, 109.95, 53.70, 44.94, 41.15, 32.80, 32.65, 31.90, 29.96.

#### 3.2.27. *Tert-butyl 4-((2-nitrophenyl)amino)piperidine-1-carboxylate* (**56**)

1-fluoro-2-nitrobenzene (0.756 mL, 7.08 mmol) was dissolved in a stirred solution of tert-butyl 4-aminopiperidine-1-carboxylate (1.99 g, 9.92 mmol) and a suspension of fine-powdered K_2_CO_3_ (2.16 g, 15.6 mmol) in dry DMF (5 mL) under nitrogen gas. The reaction mixture was kept at 70 °C overnight. The mixture was concentrated under reduced pressure. Brine (20 mL) was added, and the product was extracted with EtOAc. The combined organic phases were washed with brine (15 mL), dried (Na_2_SO_4_) and concentrated under reduced pressure. The crude product was purified by silica gel chromatography (PE/EtOAc 9:1 to PE/EtOAc/MeOH 8.5:1:0.5) to give **56** (2.20 g, 96%) as an orange solid.

#### 3.2.28. *Tert-butyl 4-((2-aminophenyl)amino)piperidine-1-carboxylate* (**57**)

Compound **56** (2.00 g, 6.22 mmol) was dissolved in a stirred suspension of Pd/C (10%, (10%, 0.660 g, 0.62 mmol) in the methanol (40 mL) under atmospheric pressure of H_2_. The suspension was filtered and concentrated under reduced pressure. The crude product was purified by silica gel chromatography (DCM to DCM/MeOH 99:1) to give **57** (1.79 g, 99.0%) as a violet solid, stored under nitrogen atmosphere.

#### 3.2.29. *Tert-butyl 4-(2-(cyanoimino)-2,3-dihydro-1H-benzo[d]imidazol-1-yl)piperidine-1-carboxylate* (**58**)

Compound **57** (0.300 g, 1.03 mmol) was dissolved to a stirred solution of diphenyl cyanocarbonimidate (0.245 g, 1.03 mmol) and DIPEA (0.350 mL, 2.06 mmol) in ACN (24 mL). The reaction was heated under reflux and nitrogen atmosphere overnight. The reaction mixture was cooled to 0 °C and filtered to isolate **58** (0.218 g, 62.3%) as a white solid. Rf = 0.35 (DCM/MeOH 97:3). MS (ESI): *m/z* 342 [M + H]^+^; ^13^C NMR: (151 MHz, CDCl_3_): δ = 154.72, 153.28, 129.39, 129.18, 122.87, 122.54, 118.46, 110.84, 110.52, 80.27, 52.35, 28.66, 28.18.

#### 3.2.30. *N-(1-(piperidin-4-yl)-1,3-dihydro-2H-benzo[d]imidazol-2-ylidene)cyanamide* (**59**)

Compound **58** (0.218 g, 0.639 mmol) was dissolved in a stirred solution of TFA/DCM (10%, 11.0 mL) at room temperature. After 5 h, the reaction mixture was concentrated under reduced pressure. NaHCO_3_ was added, and the product was extracted with DCM (5 × 15 mL). The combined organic phases were washed with brine, dried (Na_2_SO_4_), filtered and concentrated under reduced pressure to give **58** (0.130 g, 84.4%) as a white-yellow solid.

#### 3.2.31. *N-(1-(1-(3-(2-chlorophenyl)propanoyl)piperidin-4-yl)-1,3-dihydro-2H-benzo[d]imidazol-2-ylidene)cyanamide* (**15**)

Compound **29** (0.184 g, 0.539 mmol) was dissolved in solution of CDI (0.087 g, 0.539 mmol) in DCM (3 mL). After 30′, a solution of **59** (0.093 g, 0.304mmol) and DIPEA (0.458 mL, 2.69 mmol) in DCM (2 mL) was added to the reaction mixture and stirred overnight at room temperature. The reaction mixture was washed with water (3 × 15 mL), brine (50 mL), dried (Na_2_SO_4_) and concentrated under reduced pressure. The residue was purified by silica gel chromatography (DCM/MeOH 95:5) to give **15** (0.080 g, 36.4%) as a white solid. R_f_ = 0.25 (DCM/MeOH 95:5); MS (ESI): *m/z* 409/411 [MH^+^]; ^1^H NMR (600 MHz, DMSO-d6): δ = 7.43–7.38 (m, 4H, ArH), 7.29–7.06 (m, 4H, BzH), 4.79 (d, J = 13.0, 1H, NCHH), 4.64–4.58 (m, 1H, CH), 4.03 (d, J = 13.7, 1H, NC’HH), 3.10 (t, J = 12.8, 1H, NCHH), 2.95 (t, J = 7.6, 2H, ArCH_2_), 2.76–2.72 (m, 1H, NC’HH), 2.71–2.60 (m, 2H, COCH_2_), 2.23 (tt, J = 12.5, 7.8, 1H, CHCHH), 2.10 (qd, J = 12.7, 4.6, 1H, CHC’HH), 1.77 (d, J = 12.1, 2H, CHCH_2_); ^13^C NMR: (151 MHz, DMSO-d6): δ = 172.58, 170.03, 162.86, 139.26, 133.50, 131.35, 131.31, 129.70, 128.51, 127.80, 122.21, 112.00, 52.55, 44.94, 41.23, 40.57, 36.33, 32.60, 29.19, 21.60.

#### 3.2.32. *1-(3-(2-chlorophenyl)propanoyl)-1,3-dihydro-2H-benzo[d]imidazol-2-one* (**16**)

Compound **29** (1 g, 5.42 mmol) was dissolved in SOCl_2_ (5 mL) and heated under reflux for 1 h. Meanwhile, in a second flask, the 1,3-dihydro-2H-benzo[d]imidazol-2-one (726 mg, 5.42 mmol) and sodium hydride 60% dispersion in mineral oil (217 mg, 5.42 mmol) were dissolved in DMF and stirred at room temperature for 1h. Solvent was evaporated from flask one and the formed acyl chloride was dissolved in dry DMF, slowly transferred in the reaction mixture of flask 2 and stirred for 3 h at room temperature. The solvent was evaporated under reduced pressure. Extraction was performed with ethyl acetate (3 × 50 mL) and a saturated solution of ammonium chloride (50 mL). The combined organic phases were washed with brine (150 mL), dried (Na_2_SO_4_) and concentrated under reduced pressure. The crude product was purified by silica gel chromatography (petroleum ether/ethyl acetate 9:1 to 7:3) to give **16** as a white solid. Rf = 0.40 (petroleum ether/ethyl acetate/MeOH 8:1.5:0.5). ^1^H NMR (600 MHz, CDCl_3_) δ 11.37 (s, 1H, NH), 7.97 (d, J = 7.9 Hz, 1H, 3′ArH), 7.42–7.36 (m, 2H, 4′, 7′BzImH), 7.29–7.11 (m, 3H, 4′, 5′, 6′ArH), 7.09–6.97 (m, 2H, 5′, 6′ BzImH), 3.34 (t, J = 7.6 Hz, 2H, COCH_2_CH_2_), 3.05 (t, J = 7.5 Hz, 2H, COCH_2_CH_2_).

#### 3.2.33. General Procedure for Compounds **17**, **20** and **60**–**62**

Compound **16** (1 mmol) and the appropriate base (2 mmol) were dissolved in THF. The appropriate α-bromoacetate ester, acrylate ester or acetonitrile (2.5 mmol) was added. The reaction mixture was stirred at room temperature overnight. The solvent was concentrated under vacuum.

*Ethyl 2-(3-(3-(2-chlorophenyl)propanoyl)-2-oxo-2,3-dihydro-1H-benzo[d]-imidazol-1-yl)acetate* (**17**): The reaction was run with **16** (150 mg, 0.499 mmol), ethyl bromoacetate (208 mg, 1.24 mmol) and DBU (150 μL, 0.997 mmol) in THF (5 mL). After completion, the reaction mixture was extracted with DCM (3 × 10 mL) and HCl 0.5N (10 mL). The combined organic phases were washed with brine (30 mL), dried (Na_2_SO_4_) and concentrated under reduced pressure to give **17** as a white solid (147mg, 76.2%). Rf = 0.25 (petroleum ether/ethyl acetate 95:5). ^1^H NMR (600 MHz, CDCl_3_) δ = 8.23 (d, J = 7.9 Hz, 1H, 3′ArH), δ = 7.34 (m, J = 7.6 Hz, 2H, 4′, 5′ArH), δ = 7.23–7.10 (m, 4H, BzImH), δ = 6.85 (d, J = 7.6 Hz, 1H, 6′ArH), δ = 4.57 (s, 2H, NCH_2_COOEt), 4.23 (q, J = 7.0 Hz, 2H, OCH_2_CH_3_), δ = 3.50 (t, J = 7.6 Hz, 2H, COCH_2_CH_2_), δ = 3.21 (t, J = 7.6 Hz, 2H, COCH_2_CH_2_), δ = 1.27 (t, J = 7.0 Hz, 3H, OCH_2_CH_3_).

*Ethyl 2-(3-(3-(2-chlorophenyl)propanoyl)-2-oxo-2,3-dihydro-1H-benzo[d] imidazol-1-yl)propanoate* (**20**): Compound **16** (120 mg, 0.400 mmol) was dissolved in a solution of DBU (120 μL, 0.800 mmol) in ethyl acrylate (1 mL). After completion, the reaction mixture was extracted with DCM (3 × 10 mL) and HCl 0.5N (10 mL). The combined organic phases were washed with brine (30 mL), dried (Na_2_SO_4_) and concentrated under reduced pressure to give **20** as a white solid (112 mg, 70.8%). Rf = 0.62 (petroleum ether/ethyl acetate 9:1). ^1^H NMR (600 MHz, CDCl_3_) δ = 8.23 (d, J = 7.9 Hz, 1H, 3′ArH), 7.34 (m, J = 7.6 Hz, 2H, 4′,5′ArH), 7.21 (t, J = 7.8 Hz, 1H, ArH), 7.17 (t, J = 7.5 Hz, 1H, ArH) 7.23–7.10 (m, 2H, BzImH), 7.05 (d, J = 7.6 Hz, 1H, ArH), 4.11 (t, J = 7.1 Hz, 2H, CH_2_CH_3_), 4.10–4.07 (m, 2H, NCH_2_COOEt), 3.53–3.43 (m, 2H, ArCH_2_), 3.19 (t, J = 7.7 Hz, 2H, CH_2_COOEt), 2.75 (t, J = 7.0 Hz, 2H, CH_2_COON), 1.19 (t, J = 7.2 Hz, 3H, CH_3_). ^13^C NMR (151 MHz, CDCl_3_), δ = 172.49, 170.94, 152.03, 138.21, 134.13, 130.75, 129.55, 129.45, 127.82, 126.93, 126.32, 124.65, 122.84, 115.90, 107.81, 61.08, 37.16, 37.09, 32.61, 28.04, 14.14.

*Tert-butyl 2-(3-(3-(2-chlorophenyl)propanoyl)-2-oxo-2,3-dihydro-1H-benzo-[d]imidazol-1-yl)acetate* (**60**): The reaction was run with **16** (160 mg, 0.532 mmol) and sodium hydride (21.3 mg, 0.532 mmol) in THF (2 mL) and DMF (1 mL) under nitrogen atmosphere at 0°. The reaction mixture was stirred at room temperature for 2 h. Tert-butyl bromoacetate (79 μL, 0.532 mmol) was added dropwise and the reaction mixture was stirred for 1 h at room temperature. Solvents were evaporated, water was added (15 mL) and the mixture was extracted with DCM (3 × 15 mL), washed with brine, dried (Na_2_SO_4_) and concentrated under reduced pressure. The crude product was purified by silica gel chromatography (DCM/petroleum ether 5:5 to 6:4) to give **60** as a white solid (100 mg, 45.2%). Rf = 0.22 (petroleum ether/ethyl acetate 7:3). ^1^H NMR (600 MHz, CDCl_3_) δ = 8.21 (d, J = 7.8 Hz, 1H, 3’ArH), δ = 7.40–7.27 (m, J = 7.8 Hz, 2H, 4’,5’ArH), δ = 7.22–7.03 (m, 4H, BzImH), δ = 6.83 (d, J = 7.7 Hz, 1H, 6’ArH), δ = 4.46 (s, 2H, NCH_2_CO), δ = 3.55–3.42 (t, 2H, COCH_2_CH_2_), δ = 3.20 (t, J = 7.7 Hz, 2H, COCH_2_CH_2_), 1.45 (s, 9H, OCC_3_H_9_). ^13^C NMR (151 MHz, CDCl_3_) δ = 172.53, 166.19, 152.30, 138.26, 134.19, 130.80, 129.60, 127.87, 127.00, 126.38, 124.74, 123.18, 116.08, 107.53, 83.31, 77.42, 77.20, 76.99, 42.88, 37.20, 29.81, 28.09.

*2-(3-(3-(2-chlorophenyl)propanoyl)-2-oxo-2,3-dihydro-1H-benzo[d]imidazol-1-yl)acetonitrile* (**61**): Compound **16** (100 mg, 0.333 mmol) was dissolved in dry DMF (3 mL). Sodium hydride 60% dispersion in mineral oil was added and the reaction mixture stirred for 1 h at room temperature. Bromoacetonitrile was then added (35 μL, 0.499 mmol) and the reaction mixture stirred for 2 h. The mixture was diluted with water (5 mL) and extracted with DCM (3 × 10 mL), washed with brine (30 mL), dried (Na_2_SO_4_) and concentrated under reduced pressure. The crude product was purified by silica gel chromatography (petroleum ether/ethyl acetate 8:2) to give **61** as a solid (60 mg, 54.5%). Rf = 0.21 (petroleum ether/ethyl acetate 9:1). ^1^H NMR (600 MHz, CDCl_3_) δ = 8.24 (d, J = 7.9 Hz, 1H, 3’ArH), δ = 7.34 (td, J = 7.5, 1.3 Hz, 2H,4’,5’ArH), δ = 7.30 (td, J = 7.7, 0.8 Hz, 1H,6’ArH), δ = 7.24 (td, J = 7.9, 1.0 Hz, 1H, 4’BzImH), δ = 7.21–7.13 (m, 2H,5′, 6′BzImH), δ = 7.12–7.06 (m, 1H, 7′BzImH), δ = 4.76 (s, 2H, NCH_2_CN), δ = 3.48 (t, J = 7.7 Hz, 2H, COCH_2_CH_2_), δ = 3.21 (t, J = 7.6 Hz, 2H, COCH_2_CH_2_). ^13^C NMR (151 MHz, CDCl_3_) δ = 172.21, 151.33, 137.97, 134.19, 130.86, 129.71, 128.05, 127.68, 127.07, 126.46, 125.27, 124.31, 116.51, 113.23, 107.70, 77.37, 77.16, 76.95, 37.23, 28.82, 28.09.

*Tert-butyl 2-(3-(3-(2-chlorophenyl)propanoyl)-2-oxo-2,3-dihydro-1H-benzo-[d]imidazol-1-yl)propanoate* (**62**): Compound **16** (110 mg, 0.366 mmol) was dissolved in a solution of DBU (109 μL, 0.732 mmol) in t-Butyl acrylate (1 mL). After completion, the reaction mixture was extracted with DCM (3 × 10 mL) and NH_4_Cl (Saturated solution, 10 mL). The combined organic phases were washed with brine (30 mL), dried (Na_2_SO_4_) and concentrated under reduced pressure. The crude product was purified by silica gel chromatography (Petroleum ether/Ethyl Acetate 95:5 to 9:1) to give **62** as a white solid (97.3 mg, 62.1%). Rf= 0.62 (petroleum ether/ethyl acetate 9:1). ^1^H NMR (600 MHz, CDCl_3_) δ = 8.19 (t, J = 10.7 Hz, 1H, ArH), 7.21 (t, J = 7.7 Hz, 1H, ArH), 7.18 (t, J = 7.3 Hz, 1H, ArH), 7.16–7.10 (m, 2H, ArH), 7.05 (d, J = 7.8 Hz, 1H), 4.09 (t, J = 7.2 Hz, 2H, NCH_2_), 3.49 (t, J = 7.7 Hz, 2H, ArCH_2_), 3.20 (t, J = 7.7 Hz, 2H, CH_2_CON), 2.67 (t, J = 7.2 Hz, 2H, CH_2_COOt-Bu), 1.38 (s, 9H, CH_3_).^13^C NMR (151 MHz, CDCl_3_), δ = 172.59, 170.17, 152.06, 138.25, 134.17, 130.78, 129.60, 129.50, 127.85, 126.97, 126.36, 124.68, 122.87, 115.94, 107.92, 81.53, 37.20, 33.82, 28.09, 28.06.

*2-(3-(3-(2-chlorophenyl)propanoyl)-2-oxo-2,3-dihydro-1H-benzo[d]-imidazol-1-yl)acetic acid* (**18**): Compound **60** (100 mg, 0.291 mmol) was dissolved in DCM (2ml), and trifluoroacetic acid (185 μL, 2.41mmol) was added and the reaction stirred at room temperature overnight. Solvent was evaporated under reduced pressure and the obtained solid was washed with iced DCM to give **18** as a white solid (102.1 mg, 98.2%). R_f_ = 0.25 (DCM/MeOH 8:2); MS (ESI): *m/z* 359/361 [MH^+^]; ^1^H NMR (600 MHz, CDCl_3_) δ = 8.21 (d, J = 7.8 Hz, 1H, 3’ArH), δ = 7.40–7.27 (m, J = 7.8 Hz, 2H, 4’,5’ArH), δ = 7.22–7.03 (m, 4H, BzImH), δ = 6.83 (d, J = 7.7 Hz, 1H, 6’ArH), δ = 4.46 (s, 2H, NCH_2_CO), δ = 3.55–3.42 (t, 2H, COCH_2_CH_2_), δ = 3.20 (t, J = 7.7 Hz, 2H, COCH_2_CH_2_). ^13^C NMR (151 MHz, CDCl_3_) δ = 172.53, 166.19, 152.30, 138.26, 134.19, 130.80, 129.60, 127.87, 127.00, 126.38, 124.74, 123.18, 116.08, 107.53, 83.31, 77.42, 77.20, 76.99, 42.88, 37.20, 29.81.

#### 3.2.34. *1-((2H-tetrazol-5-yl)methyl)-3-(3-(2-chlorophenyl)propanoyl)-1,3-dihydro-2H-benzo[d]imidazol-2-one* (**19**)

Compound **61** (60 mg, 0.177 mmol) was dissolved in dry DMF (2 mL). Sodium azide (35 mg, 0.530 mmol) and ammonium chloride (9.5mg, 0.177mmol) were added, and the reaction mixture was stirred at 120 °C for 2 h. The solvent was evaporated under reduced pressure. Extraction was performed with ethyl acetate (3 × 10 mL) and a saturated ammonium chloride aqueous solution (10 mL). The combined organic phases were washed with brine (30 mL), dried (Na_2_SO_4_) and concentrated under reduced pressure. The crude product was purified by silica gel chromatography (petroleum ether/ethyl acetate/methanol 80:15:5) to give **19** as a white solid (45 mg, 66.1%). Rf = 0.13 (petroleum ether/ethyl acetate 7:3).

#### 3.2.35. *2-(3-(3-(2-chlorophenyl)propanoyl)-2-oxo-2,3-dihydro-1H-benzo[d]- imidazol-1-yl)propionic acid* (**21**)

Compound **62** (100 mg) was dissolved in a solution of trifluoroacetic acid (0.7 mL) in DCM (7 mL), and the mixture was stirred at room temperature overnight. Solvent was evaporated under reduced pressure. The crude product was purified by silica gel chromatography (DCM/MeOH 97:3) to give **21** as a white solid (87 mg, 99.5%). Rf = 0.51 (DCM/MeOH 9:1). ^1^H NMR (600 MHz, CDCl_3_) δ = 8.17 (d, J = 7.9 Hz, 1H, ArH), 7.31 (dd, J = 7.8, 1.2 Hz, 2H, ArH), 7.19 (t, J = 7.6 Hz, 1H, ArH), 7.16 (t J = 7.4 Hz, 1H, ArH), 7.14–7.09 (m, 2H, ArH), 7.04 (d, J = 7.8 Hz, 1H, ArH), 4.09 (t, J = 7.1 Hz, 2H, NCH_2_), 3.46 (t, J = 7.7 Hz, 2H, ArCH_2_), 3.18 (t, J = 7.7 Hz, 2H, CH_2_CON), 2.75 (t, J = 7.1 Hz, 2H, CH_2_COOH). ^13^C NMR (151 MHz, CDCl_3_) δ = 173.96, 172.62, 152.13, 138.15, 134.11, 130.75, 129.57, 129.38, 127.86, 126.97, 126.31, 124.72, 122.95, 115.95, 107.88, 77.37, 77.16, 76.95, 37.17, 36.96, 32.20, 28.04.

#### 3.2.36. *N-(2-chlorophenethyl)-2-nitroaniline* (**63**)

2-(2-chlorophenyl)ethan-1-amine (0.649 mL, 4.61 mmol) was added to a stirred solution of 2-fluoronitrobenzene (0.373 mL, 3.54 mmol) and K_2_CO_3_ in DMF (3 mL). The reaction was kept under nitrogen atmosphere at 70 °C for 18h. The solvent was evaporated under reduced pressure. The residue was dissolved in DCM (20 mL) and washed with water (3 × 20 mL). The crude product was purified by silica gel chromatography (Pe/EtOAc 9:1) to give **63** as an orange solid (978 mg, 99.5%). Rf = 0.58 (Pe/EtOAc 9:1). ^1^H NMR (600 MHz, CDCl_3_) δ = 8.16 (dd, J = 8.5, 1.5 Hz, 1H, ArH), 7.44–7.41 (m, 1H, ArH), 7.38 (dd, J = 7.7, 1.5 Hz, 1H, ArH, 7.27 (dd, J = 7.2, 1.7 Hz, 1H, ArH), 7.24–7.18 (m, 2H, ArH), 6.91 (d, J = 7.9 Hz, 1H, ArH), 6.65–6.63 (m, 1H, ArH), 3.60 (t, J = 7.2 Hz, 2H, NCH_2_), 3.14 (t, J = 7.2 Hz, 2H, ArCH_2_)

#### 3.2.37. *N-(2-chlorophenethyl)benzene-1,2-diamine* (**64**)

Compound **63** (0.93 g, 3.66 mmol) was dissolved in a stirred suspension of Pd/C (10%, 0.358 g, 0.34 mmol) in methanol (20 mL) under atmospheric pressure of H_2_. The suspension was filtered and concentrated under reduced pressure. The crude product was purified by silica gel chromatography (PE/EtOAc 9:1) to give **64** (764 mg, 92.0%) as a white solid. Rf = 0.21 (Pe/EtOAc 9:1). ^1^H NMR (600 MHz, CDCl_3_) δ = 7.39–7.37 (m, 1H), 7.34–7.31 (m, 1H), 7.27–7.18 (m, 3H), 6.85–6.77 (m, 2H), 6.75–6.72 (m, 1H), 3.41 (t, J = 7.2 Hz, 2H, NCH_2_), 3.00 (t, J = 7.2 Hz, 2H, ArCH_2_).

#### 3.2.38. *1-(2-chlorophenethyl)-1,3-dihydro-2H-benzo[d]imidazol-2-one* (**65**)

CDI (1.05 g, 6.49 mmol) was added to a stirred solution of **64** (0.80 mg, 3.24 mmol) in DCM (50mL). After monitoring the end of the reaction (TLC), the solution was washed with water (3 × 20 mL). The organic solvent was evaporated under reduced pressure. The crude product was purified by silica gel chromatography (Pe/EtOAc 7:3) to give **65** as a white solid (170 mg, 78.0%). Rf = 0.39 (Pe/EtOAc/MeOH 8:1.5:0.5). ^1^H NMR (600 MHz, DMSO-D6) δ = 10.78 (s, 1H, BzImNH), 7.36–7.34 (m, 1H, ArH), 7.24–7.18 (m, 3H, ArH), 6.94–6.90 (m, 4H, ArH), 3.98 (t, J = 7.2 Hz, 2H, NCH_2_), 3.03 (t, J = 7.2 Hz, 2H, ArCH_2_).

#### 3.2.39. *Ethyl 2-(3-(2-chlorophenethyl)-2-oxo-2,3-dihydro-1H-benzo[d]imidazol-1-yl) acetate* (**22**)

Compound **65** (200 mg, 0.733 mmol) was dissolved in a solution of DBU (0.219 μL, 1.466 mmol) and ethyl 2-bromoacetate (0.163 mL, 1.47 mmol) in dry THF (5 mL). After completion, water (10 mL) was added, and the product was extracted with DCM (3 × 10 mL). The combined organic phases were washed with brine (30 mL), dried (Na_2_SO_4_) and concentrated under reduced pressure. The crude product was purified by silica gel chromatography (Pe/EtOAc 8:2) to give **22** as a white solid (190 mg, 73.2%). Rf = 0.53 (Pe/EtOAc/MeOH 8:1.5:0.5). ^1^H NMR (600 MHz, CDCl_3_) δ = 7.29 (d, J = 1.7 Hz, 1H), 7.28–7.33 (m, 2H), 7.25 (d, J = 1.7 Hz, 1H), 7.23 (d, J = 2.1 Hz, 1H), 7.18–7.26 (m, 1H), 6.85–6.84 (m, 2H), 4.86–4.83 (m, 2H), 3.72 (t, J = 6.5 Hz, 2H), 2.97 (m, 4H), 1.84–1.82 (m, 3H).

#### 3.2.40. *Tert-butyl 2-(3-(2-chlorophenethyl)-2-oxo-2,3-dihydro-1H-benzo[d]imidazol-1-yl) acetate* (**66**)

Compound **65** (100 mg, 0.367 mmol) was dissolved in a solution of DBU (0.110 μL, 0.733 mmol) and tert-butyl 2-bromoacetate (0.108 mL, 0.733 mmol) in dry THF (3 mL). After completion, water (10 mL) was added, and the product was extracted with DCM (3 × 10 mL). The combined organic phases were washed with brine (30 mL), dried (Na_2_SO_4_) and concentrated under reduced pressure. The crude product was purified by silica gel chromatography (Pe/EtOAc 8:2) to give **66** as a white solid (102 mg, 75.1%). Rf = 0.50 (Pe/EtOAc/MeOH 8:1.5:0.5).). ^1^H NMR (600 MHz, CDCl_3_) δ = 8.25–8.35 (m, 2H), 7.65 (d, J = 7.5 Hz, 1H), 7.41–7.50 (m, 2H), 7.21–7.10 (m, 3H), 4.59 (t, J = 7.1 Hz, 2H), 4.05 (q, J = 7.9 Hz, 2H), 3.32 (t, J = 6.5 Hz, 2H), 2.89 (t, J = 7.1 Hz, 4H), 2.61 (t, J = 7.9 Hz, 4H), 1.38 (t, J = 7.8 Hz, 3H). ^13^C NMR (151 MHz, CDCl_3_) δ = 166.8, 154.1, 135.8, 134.2, 131.5, 129.6, 128.9, 128.7, 128.4, 127.2, 121.8, 121.4, 107.9, 107.8, 43.1, 43.0, 41.0, 34.8, 32.8, 28.1

#### 3.2.41. *2-(3-(2-chlorophenethyl)-2-oxo-2,3-dihydro-1H-benzo[d]imidazol-1-yl)acetic acid* (**23**)

Compound **66** (100 mg) was added in a stirred solution of TFA (0.300 mL) in DCM (3 mL). After 18 h, the solvents were evaporated under reduced pressure. The crude product was purified by preparative HPLC (ACN/H_2_O 6:4) to give **23** as a white solid (45 mg, 50.5%). Rf = 0.12 (DCM/MeOH 9:1). ^1^H NMR (600 MHz, DMSO-D6) δ = 7.37 (d, J = 7.2 Hz, 1H), 7.27 (d, J = 6.9 Hz, 1H), 7.19 (t, J = 7.6 Hz, 2H), 7.09–7.12 (m, 1H), 7.01–6.98 (m, 3H), 4.04 (t, J = 7.1 Hz, 2H), 3.04 (t, J = 7.1 Hz, 2H), 2.46 (s, 3H). ^13^C NMR (151 MHz, DMSO-D6) δ = 170.2, 153.9, 138.8, 136.2, 133.7, 131.9, 129.8, 129.3, 127.9, 126.9, 121.6, 121.4, 108.7, 108.1, 40.7, 34.4, 32.2.

### 3.3. Biological Evaluation

#### 3.3.1. Cells and Treatments

Human myelomonocytic THP-1 cells were cultured in RPMI 1640 medium (Aurogene, Rome, Italy) supplemented with fetal bovine serum (10%; Aurogene), L-glutamine (2 mM; Aurogene), penicillin (100 IU/mL; Aurogene) and streptomycin (100 mg/mL; Aurogene). Cell culture medium was replaced every 2−3 days, and the cultures were maintained at 37 °C and 5% CO_2_ in a fully humidified incubator. The day before each experiment, cells were plated in 48-well culture plates (90.000 cells/well) and were differentiated by treatment with PMA (50 nM, 24 h; Sigma-Aldrich). PMA-differentiated THP-1 cells were washed twice with phosphate-buffered saline (PBS) and primed with LPS (10 μg/mL, 4 h; Sigma-Aldrich) in serum-free medium. Cells were then incubated with compounds dissolved in medium containing 0.1% DMSO for 1 h and cell death was triggered with ATP (5 mM, 90 min; Sigma-Aldrich). MCC950 (Sigma-Aldrich batch #45216 and batch #85021 and from Crysdot (product n. CD31002496; OS05876-18070932) was used in the experiments.

#### 3.3.2. LDH Release Measurement

Cell death was quantified by using the CytoTox 96 nonradioactive cytotoxicity assay (Promega Corporation, Madison, MI, USA): LDH activity was determined in differentiated THP-1 supernatant, after 1.5 h from ATP treatment, by measuring absorbance using the Victor X4 (PerkinElmer, Waltham, MA, USA) at λ = 490 nm. Cell death was expressed according to the manufacture’s instruction.

#### 3.3.3. IL-1β Release

IL-1β release was quantified in differentiated THP-1 supernatant, obtained as previously described, using Human IL-1 beta Uncoated ELISA kit (Invitrogen, Waltham, MA, USA), according to the manufacture’s instruction.

#### 3.3.4. Cytotoxicity Assay

THP-1 were plated in 96-wells culture plates (15.000 cells/well) and were treated with increasing concentrations (0.1–100 μM) of each compound. Cell viability was measured at 72 h by the MTT assay, a colorimetric assay based on the conversion of the water-soluble 3-(4,5-dimethylthiazol-2-yl)- 2,5-diphenyltetrazolium bromide (MTT; Sigma-Aldrich) to an insoluble purple formazan by actively respiring cells. The formazan concentration was determined by measuring absorbance in the Victor X4 at a λ = 570 nm.

### 3.4. In Vitro Measurement of NLRP3 ATPase Activity

#### 3.4.1. Generation and Capture of NLRP3-GFP Protein

Human NLRP3 (NM_004895.4) was cloned into the pAcGFP-N1 vector (ClonTech, Product# 632469) as previously described [46]. For heterologous expression, the NLRP3-GFP plasmid was transfected into HEK293T cells with PolyJet (SignaGen Laboratories, Catalog #SL100688) according to the manufacturer’s instructions. Approximately 12 h after transfection, cell lysates were prepared in GFP-Trap Lysis Buffer (10 mM Tris-HCl pH 7.5, 150 mM NaCl, 0.5 mM EDTA with 0.5% (*v/v*) NP-40). Immobilised GFP-Trap Sepharose resin was produced according to the reported procedure [49]. The GFP-Trap clone was a gift from Dr. Laura Trinkle-Mulcahy (University of Ottawa). The capture of NLRP3-GFP protein from HEK293 lysates with GFP-Trap was performed according to the Chromotek GFP-Trap protocol with some modifications. Cleared lysates were diluted 1:2 with GFP-Trap Wash Buffer (10 mM Tris-HCl pH 7.5, 150 mM NaCl, 0.5 mM EDTA) and incubated with GFP-Trap beads with end-over-end mixing for 1.5 h at 4 °C. The bead slurry was transferred to a small gravity column, and the GFP-Trap resin was washed sequentially with High-Salt Wash Buffer (10 mM Tris-HCl pH 7.5, 500 mM NaCl, 0.5 mM EDTA) and GFP-Trap Wash Buffer. For measurement of ATPase enzymatic activity, the GFP-Trap beads were subjected to a final wash of 4 × 2 mL with ADP-Glo Reaction Buffer A (40 mM Tris-HCl pH 7.5, 20 mM MgCl_2_ and 0.1 mg/mL BSA).

#### 3.4.2. Measurement of NLRP3 ATPase Activity Using ADP Glo

The ATPase activity of the NLRP3-GFP protein was determined “on-bead” following GFP-Trap capture using the luminescent ADP-Glo Kinase Assay Kit (Promega). The GFP-Trap beads were resuspended in 375 μL of ADP-Glo Reaction Buffer A, and 20 μL aliquots of the bead slurry were distributed to wells of a 96-well plate. All ATPase reactions were performed in Reaction Buffer A (40 mM Tris-HCl pH 7.5, 20 mM MgCl_2_ and 0.1 mg/mL BSA) supplemented with cOmplete™, EDTA-free Protease Inhibitor Cocktail Tablets (Millipore Sigma Cat# 11836170001). Reactions were incubated with NLRP3 inhibitor or vehicle (DMSO) and then started by addition of ATP (to give × mM final concentration). The reactions were carried out in white, flat-bottomed 96-well plates (Corning^®^ 96 Well Solid Polystyrene Microplate, Sigma Aldrich, Cat# CLS3917) with gentle-vibrational mixing at 37 °C for 10 min. After the reaction incubation period, the assay was terminated by addition of ADP-Glo Reagent (25 μL) and incubation on a shaker at room temperature for 40 min. Subsequently, Kinase Detection Reagent (50 μL) was added to each well and incubated with shaking at room temperature for 60 min before luminescence was recorded with a Spectra Max M2 plate reader. A standard curve for ATP to ADP conversion was included on each 96-well plate to convert Relative Luminescence Units (RLU) into the amount of ADP present. To account for background signal, RLUs of blank wells containing no ATP were subtracted from the reaction well values. All assays were determined to be linear with respect to time and NLRP3-GFP content.

### 3.5. Molecular Modelling

#### 3.5.1. MD Setup

Uniprot sequence Q96P20 was modelled using the SWISS-MODEL webserver, using the Cryo-EM structure of human NLRP3 (PDB ID 6npy) as a template and retaining ADP [50]. The PYD domain, already missing in the Cryo-EM structure was not modelled, given the absence of structural indication about its position with respect to the other domains. The NEK7 protein, originally present in 6npy, was removed to reduce the structural dimension and allow more profitable MD simulation. NEK7 is fundamental for NLRP3 assembly and activation but, apparently, not for ATPase activity. Despite it being unnecessary, we thus kept the NEK7 while looking for a putative binding site for the tested compounds. For assessing the quality of the model, Ramachandran plots were generated using MolProbity website [51]. As mentioned, the ADP pose was retained as in the Cryo-EM structure 6npy. The Mg^2+^ ion, known to be essential for NLRP3 activity was added and coordinated by the beta-phosphate group of ADP, as reported for other AAA+ proteins [52]. Parameters for ADP and Mg^2+^ were retrieved from Meagher et al. [53] and Allnér et al. [54], respectively. Ionizable residues were assigned the default tautomeric state at pH = 7.4, while all histidines were treated as N_ε_-H (HIE), the only exception being His522, directly involved in a polar interaction with ADP and treated as N_d_-H (HID). The system was embedded in a cubic 130 × 130 × 130 Å box, filled with TIP3P water molecules and 11 Na^+^ atoms to neutralize the protein charge. Periodic boundary conditions were set, the bond length between hydrogens and heavy atoms was kept at equilibrium distance with the LINCS algorithm. The Particle Mesh Ewald (PME) method was used for computing long-ranged interactions, and a cutoff value of 11 Å was set for both Van der Waals and electrostatic interactions. The protein converged to Fmax after 6284 steepest descent and 277 Polak–Ribière conjugate gradient minimization cycles. To gradually heat up the system from 0 to 300 K, six 1 ns thermalization steps were run with 1 fs time step in NVT ensemble using backbone restraints when heating from 0 to 200K. Just before the long MD production (1.150 µs), the system underwent a short equilibration (2 ns) in the NPT ensemble. All analyses on the trajectory were carried out using Gromacs 4.6.1, unless otherwise specified.

**Essential dynamics.** Principal modes of the protein were extracted by first fitting the trajectory to a reference structure (last equilibration step) to remove roto-translations. Then, the covariance matrix was calculated and diagonalized and eigenvectors were sorted out in descending order according to their eigenvalue. Principal component analysis (PCA) plot was obtained by plotting the trajectory in the orthogonal space described by the first vs. the second eigenvector.

#### 3.5.2. Clusters

The 750–1150 ns timeframe of the trajectory was clustered on backbone atoms, using the GROMOS method, a cutoff = 0.4 nm and a 100 ps stride. Three medoids deriving from the cluster analyses well represented the equilibrated part of the trajectory and were further analysed in docking experiments (Figure 4).

#### 3.5.3. Static Pocket Analysis

Pockets were calculated with FLAPSite [55] with standard parameters for each of the three extracted medoids, to better evaluate the immediate surroundings of the ADP site.

#### 3.5.4. Dynamic Pocket Analysis

Pockets were calculated with the Pocketron, the pocket tracker tool implemented in BiKi Life Science v. 1.3.5 software suite (http://www.bikitech.com/) [56], along the equilibrated part of the trajectory (750–1150 ns, stride = 100, with a 3 Å probe radius). This tool is aimed at identifying pockets and monitoring the crosstalk between them, i.e., the exchange of atoms between adjacent pockets [57]. The persistency of the pockets was quantified, and only pockets with >90% or more persistency were extracted and evaluated in docking studies.

#### 3.5.5. Rigid Docking

INF ligands (**6**, **9**, **13** and **18**) were drawn using MolDraw and their protonation state at pH = 7 was checked with MoKa v. 3.2.1 (Molecular Discovery Ltd.) [58]. Docking studies were carried out in GOLD v.2.2 [59] with 50 run per ligand, using default parameters and the CHEMPLP scoring functions.

#### 3.5.6. Induced Fit Docking

First, the target structures were prepared with the Protein Preparation Wizard in Maestro and protonated at a pH of 7.4 using PROPKA. The alpha carbon of Thr304 was chosen to define the target docking grid. INF ligands **6**, **9**, **13** and **18** were docked with Glide SP [60].

#### 3.5.7. Ligand Parametrization

The top-ranked pose of **9** in Med3 was chosen for assessing the stability of ligands in pocket p16. Ligand **9** was parametrized using the ab initio RESP charge fitting method included in the BiKi Life Science v. 1.3.5 software suite. Ligand-NLRP3 binary complex was then submitted to the same minimization/equilibration protocol reported above, and a 100 ns MD production in the NVT ensemble was carried out for checking out the stability of the docking pose.

## 4. Conclusions

In our continuous effort to advance the discovery of new NLRP3 inhibitors, we employed a pharmacophore-hybridization strategy to identify new structural templates endowed with the ability to dampen NLRP3 function. The merging of the pharmacophore of INF39 and HS-203873 allowed the identification of compound **1** as a prototypical template. With the aim of obtaining new non-electrophilic derivatives able to target NLRP3, the chemical modulation of **1** was performed. A preliminary screening of the synthesised compounds in an established cellular model of NLRP3 activation led to the selection of a subset of active molecules. Compounds **6**, **9**, **13** and **18**, proved able to moderately reduce NLRP3-dependent pyroptosis and IL-1β release in human macrophages. Interestingly, the selected compounds proved able to decrease the ATPase activity of immobilised NLRP3. A homology model of the inactive state of NLRP3 was built and molecular simulations and docking studies allowed for the identification of putative binding pockets for the selected compounds. A full pharmacological and mechanistic characterization of the selected compounds was out of the scope of this work, this representing an obvious limitation. However, thanks to the preliminary studies reported herein and to the model built with the use of the active scaffold, we are now working toward improved NLRP3 inhibitors.

## Figures and Tables

**Table 1:** Inhibitory effect of synthesised compounds on pyroptotic cell death, IL-1β release in differentiated THP-1 cells and cytotoxicity in THP-1 cells.

Compound.	Pyroptosis Decrease ^a^	IL-1β Inhibition ^b^	Cytotoxicity ^c^
	% inhibition at 10 µM	% inhibition at 10 µM	TC_50_ (µM)
**1**	24.9 ± 6.3 ^d^	14.4 ± 5.0	32.9 ± 19.0
**2**	14.9 ± 5.8	10.3 ± 5.8	64.1 ± 4.7
**3**	30.8 ± 9.8 ^d^	15.9 ± 5.5	65.7 ± 1.0
**4**	NA	NT	80.9 ± 3.2
**5**	NA	NT	72.6 ± 3.3
**6** (INF148)	35.0 ± 6.9 ^d^	21.0 ± 3.2 ^d^	61.7 ± 1.0
**7**	34.5 ± 5.2 ^d^	17.8 ± 7.5	62.4 ± 13.3
**8**	32.2 ± 10.6 ^d^	NT	86.3 ± 5.1
**9** (INF120)	39.2 ± 6.6 ^d^	20.3 ± 1.3 ^d^	87.1 ± 1.9
**10**	NA	NT	>100
**11**	NA	NT	76.8 ± 3.2
**12**	15.7 ± 9.1	19.0 ± 0.4	>100
**13** (INF156)	45.0 ± 8.8 ^e^	25.2 ± 4.9 ^d^	85.7 ± 5.6
**14**	NA	NT	92.6 ± 4.0
**15**	NA	NT	83.4 ± 0.8
**16**	37.7 ± 7.6 ^d^	14.9 ± 8.8	77.3 ± 3.7
**17**	25.1 ± 4.0 ^d^	24.2 ± 11.2	95.4 ± 1.1
**18** (INF172)	36.4 ± 11.1 ^d^	21.1 ± 3.1 ^d^	>100
**19**	26.2 ± 1.6 ^d^	<10	>100
**20**	24.4 ± 6.8	13.8 ± 5.4	57.6 ± 3.2
**21**	NA	NT	80.1 ± 1.4
**22**	NA	NT	92.8 ± 2.1
**23**	26.3 ± 3.5 ^d^	13.9 ± 16.4	62.4 ± 1.2
**MCC950**	42.5 ± 0.9 ^e^	37.6 ± 6.0 ^e^	>100

^a^ Pyroptosis of differentiated THP-1 cells was triggered using LPS/ATP. Data are reported as the % inhibition of pyroptosis of cells treated with 10 µM conc. of test compound vs. vehicle-treated cells. Data are the mean ± SEM of three to five experiments run in triplicate. ^b^ IL-1β inhibition was measured in the cell supernatants form the same experiments. Data are reported as % inhibition ± SEM of three to five experiments run in triplicate. ^c^ Cytotoxicity was determined after 72 h treatment of THP-1 cells with increasing conc. (0.1–100 µM) of test compounds. Data are reported as TC_50_ ± SEM of three experiments. ^d^ *p* < 0.05 vs. vehicle treated cells (t-student); ^e^ *p* < 0.01 vs. vehicle treated cells (t-student); NA = not active at 10 µM; NT = not tested.

## Data Availability

Data are available upon request from the authors.

## Supplementary Materials

Analysis of the MD trajectory, validation of the extracted reference states and pocket identification (Figures S1–S8 and Tables S1 and S2). Docking of compounds **1**, **2**, **6**, **9**, **13** and **18** in pocket p16 (Figures S9–S11). RMSD of compound **9** along 100 ns Molecular Dynamic simulation (Figure S12).

## Sample Availability

Samples of the compounds are available upon request from the authors.
