# Supplementary material for: Chemical Modulation of the 1-(Piperidin-4-yl)-1,3-dihydro-2H-benzo[d]imidazole-2-one Scaffold as a Novel NLRP3 Inhibitor
Source: Molecules. 2021 Jun 29;26(13):3975. doi: 10.3390/molecules26133975 (PMC8271538; doi:10.3390/molecules26133975)
Supplement: Supplementary file 1 [file molecules-26-03975-s001.zip › molecules-1279491-SI.pdf]

molecules

MDPI

Article

Chemical modulation of the 1-(piperidin-4-yl)-1,3-dihydro-2H-benzo[d]imidazole-2-one scaffold as a novel NLRP3 inhibitor

Simone Gastaldi [1], Valentina Boscaro [1], Eleonora Gianquinto [1], Christina F. Sandall [2], Marta Giorgis [1], Elisabetta Marini [1], Federica Blua[1], Margherita Gallicchio [1], Francesca Spyrakis [1], Justin A. MacDonald [2] and Massimo Bertinaria [1,*]

[1] Department of Drug Science and Technology, University of Turin, Via Giuria 9, 10125, Torino, Italy; simone.gastaldi@unito.it (S.G.), valentina.boscaro@unito.it (V.B.), eleonora.gianquinto@unito.it (E.G.), marta.giorgis@unito.it (M.Gi.), elisabetta.marini@unito.it (E.M.), federica.blua@unito.it (F.B.), margherita.gallicchio@unito.it (M.Ga.), francesca.spyrakis@unito.it (F.S.)

[2] Department of Biochemistry & Molecular Biology, Cumming School of Medicine, University of Calgary, 3280 Hospital Drive NW, Calgary, AB T2N 4Z6, Canada; cfsandal@ucalgary.ca (C.F.S.); jmacdo@ucalgary.ca (J.A.M.)

* Correspondence: massimo.bertinaria@unito.it (M.B.); Tel.: +39 011 6707146

Supplementary Materials

Content

Figure S1: RMSD of backbone (black) and protein (red) atoms along simulation time.

Figure S2: Principal component analysis (PCA) plot.

Figure S3: Root Mean Square Deviation (RMSD) matrix.

Table S1: Summary data from Ramachandran plots of the homology model and of Med1, Med2 and Med3.

Figure S4: Ramachandran plot for NLRP3 pre-MD homology model.

Figure S5: Ramachandran plot for NLRP3 Med1.

Figure S6: Ramachandran plot for NLRP3 Med2.

Figure S7: Ramachandran plot for NLRP3 Med3.

Figure S8: ADP pocket in medoids extracted from MD simulation.

Table S2: Centroids and radii used for docking studies in the ADP pocket.

Figure S9. Docking of **9**, **6**, **13**, **18** in pocket p16 of Med1.

Figure S10. Docking of **9**, **6**, **13**, **18** in pocket p16 of Med2.

Figure S11. Docking of **9**, **6**, **13**, **18** in pocket p16 of Med3.

Figure S12. RMSD of **9** along 100 ns MD simulation.

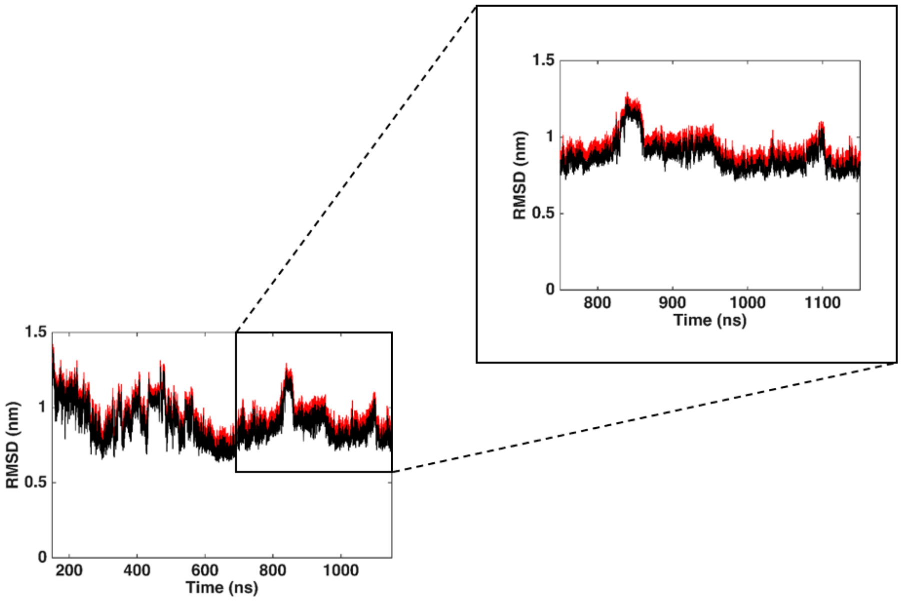

Figure S1. RMSD of backbone (black) and protein (red) atoms along simulation time (ns). The first 150ns (equilibration) were excluded from this plot and from all the subsequent analyses, the equilibrated part of the trajectory (750-1150ns) is highlighted in the rectangle**.**

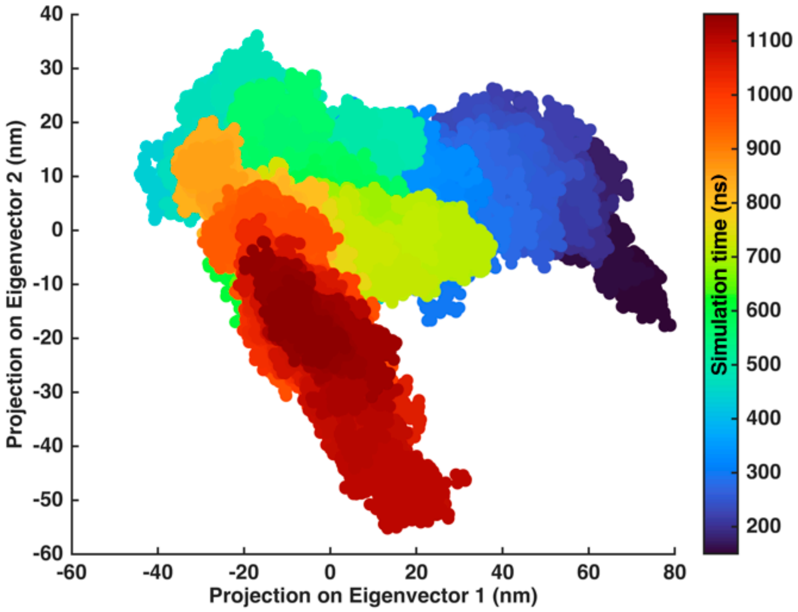

Figure S2. Principal component analysis (PCA) plot. The displacement (nm) along the first and the second eigenvector is colored according to simulation time (ns), with a rainbow palette ranging from blue to red.

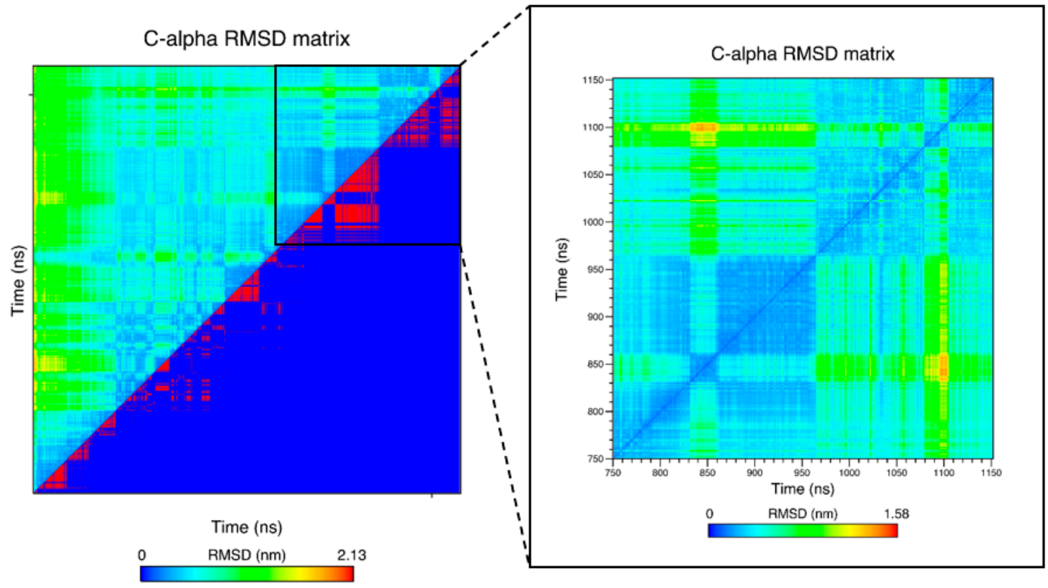

Figure S3. Root Mean Square Deviation (RMSD) matrix. RMSD matrix calculated for the trajectory (left), with a focus on the 750-1150 ns timeframe (right).

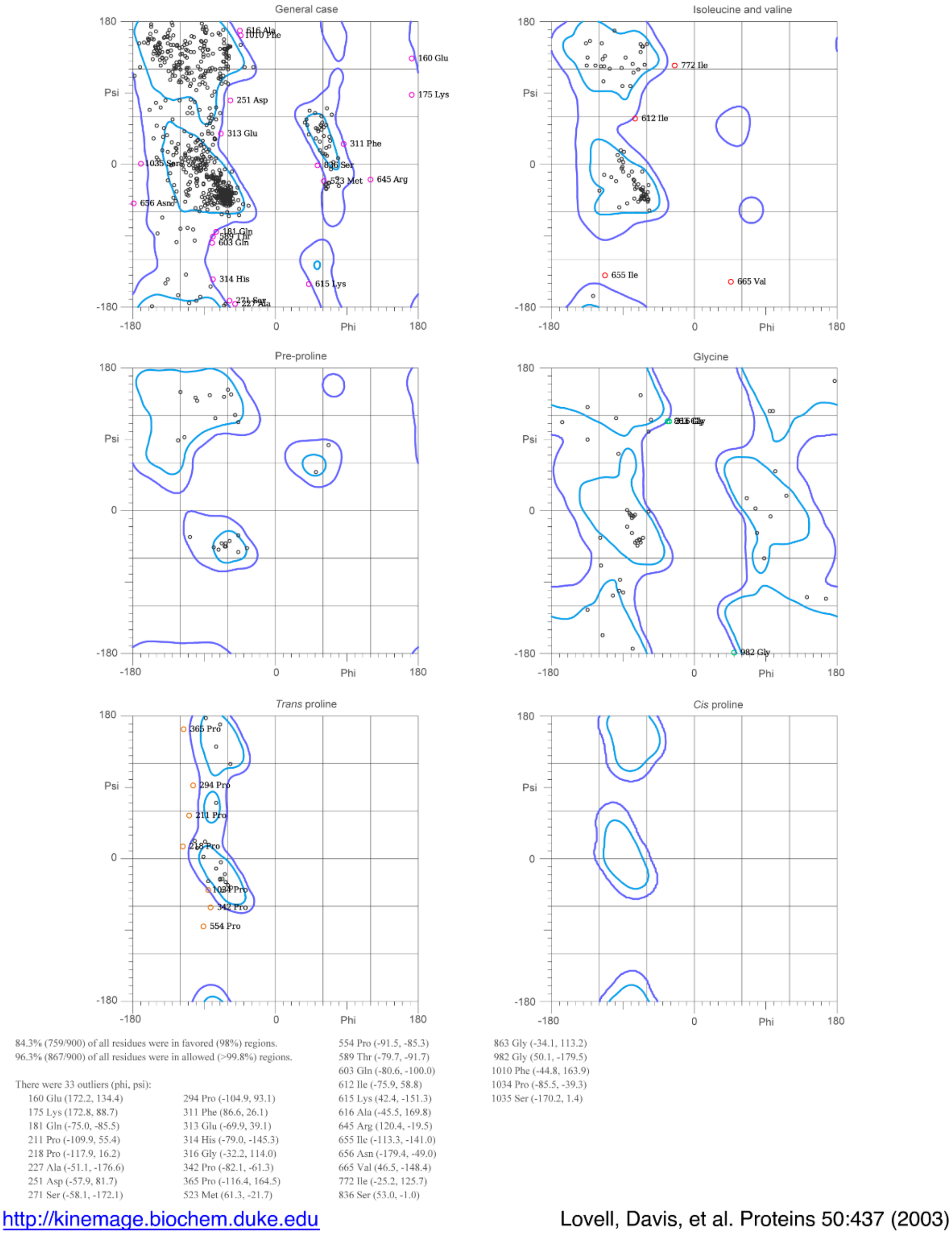

Figure S4. Ramachandran plot for NLRP3 pre-MD homology model.

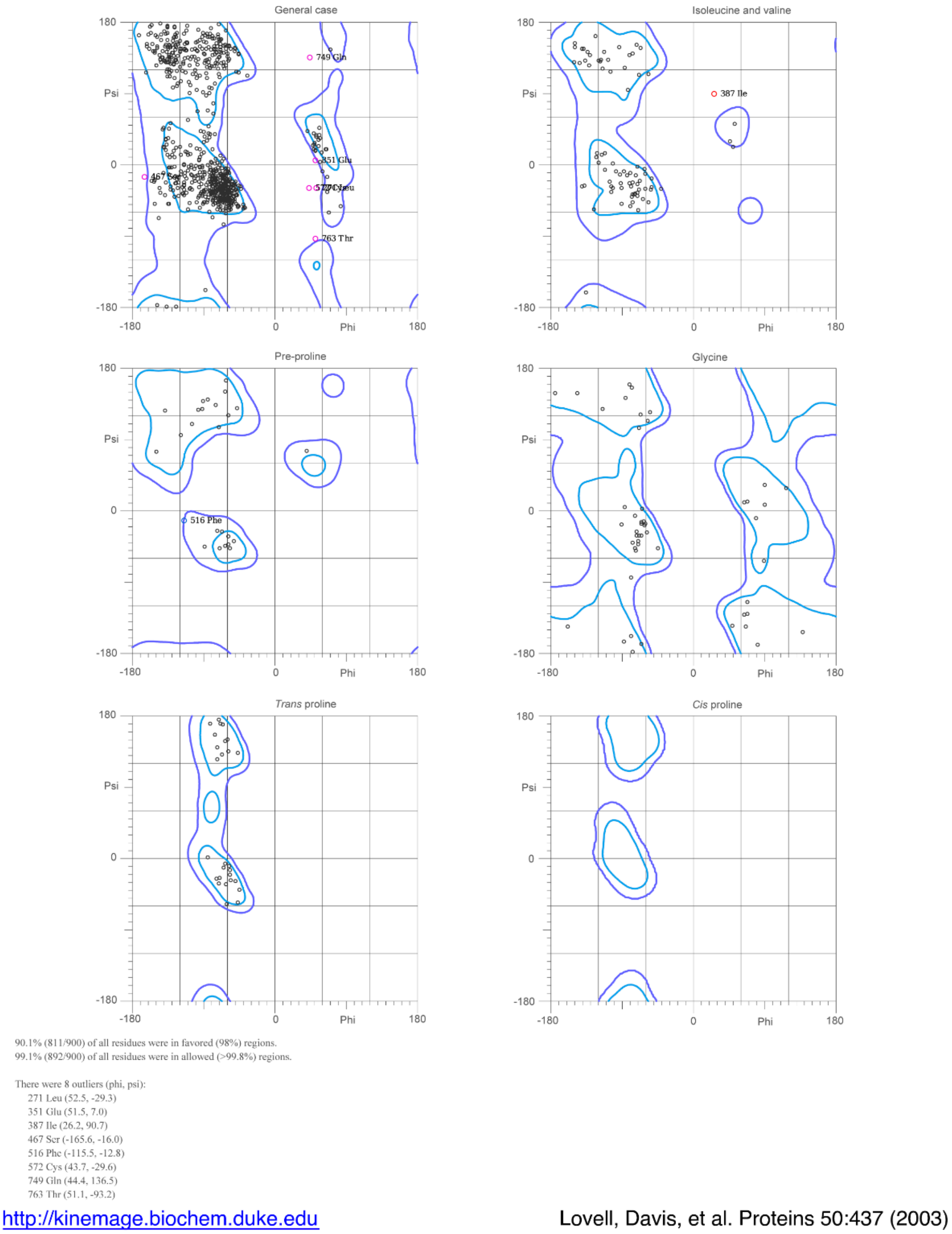

Figure S5. Ramachandran plot for NLRP3 Med1.

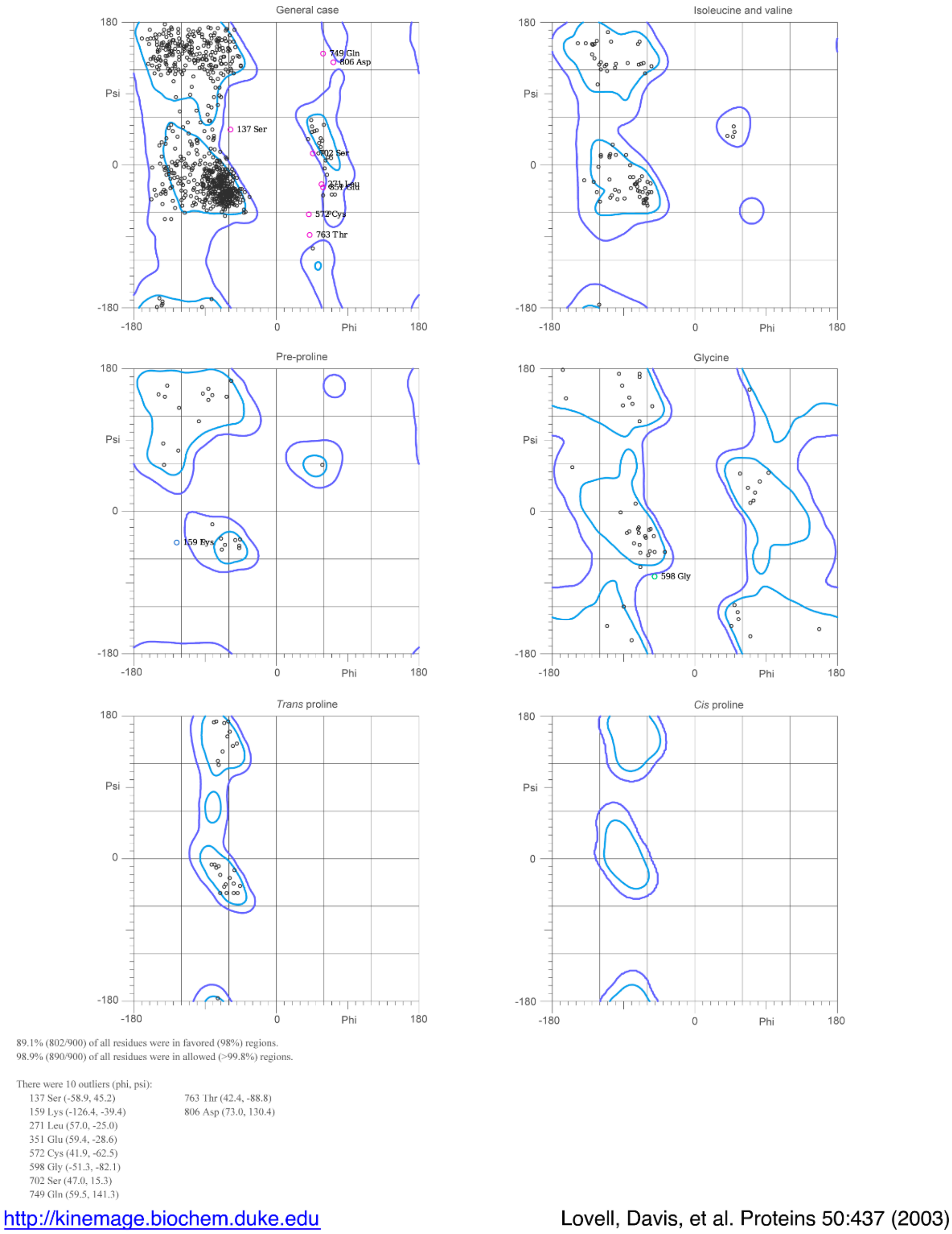

Figure S6. Ramachandran plot for NLRP3 Med2.

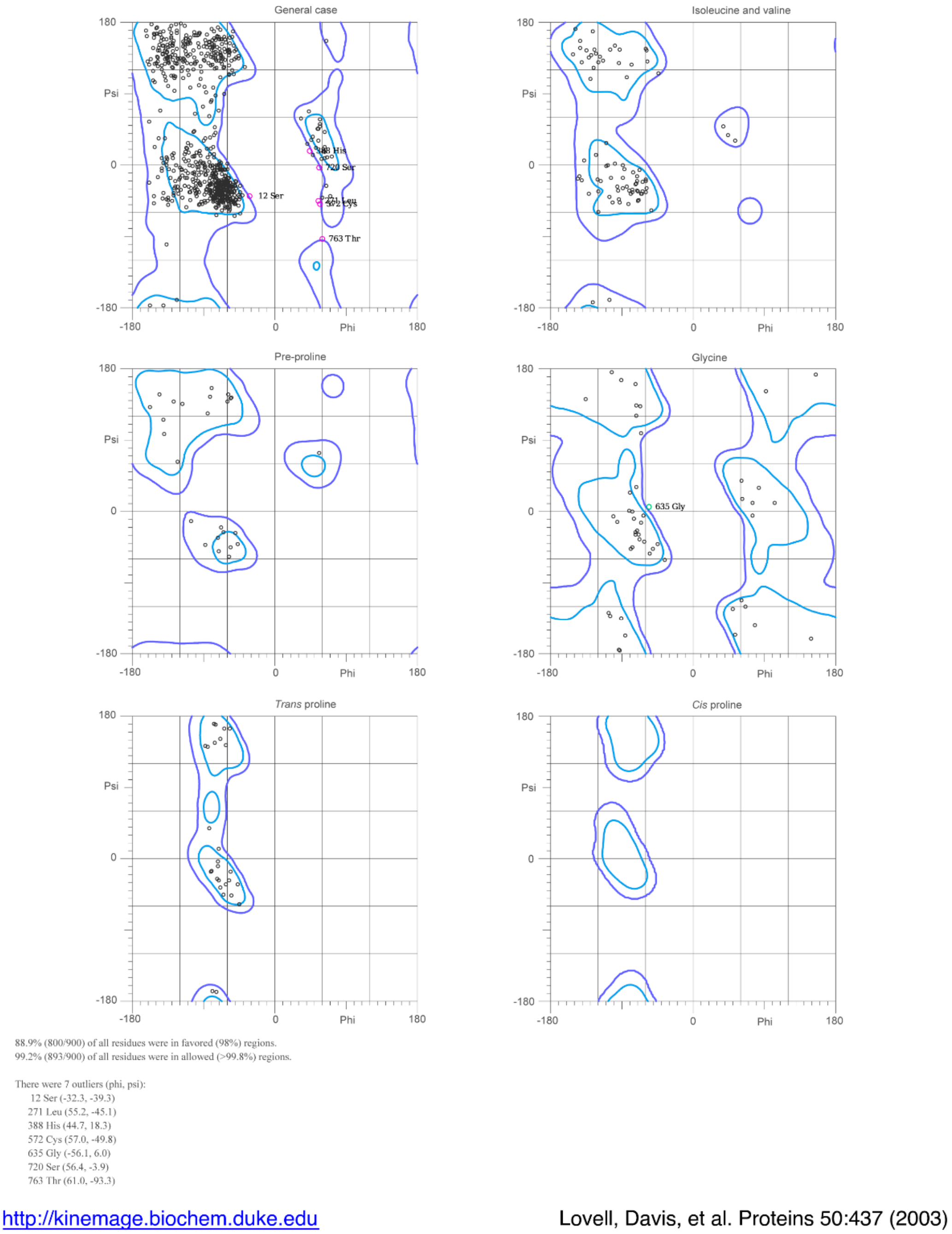

Figure S7. Ramachandran plot for NLRP3 Med3.

	Homology Model	Med1	Med2	Med3
Residues in favored regions (%)	84.3	90.1	89.1	88.9
Residues in allowed regions (%)	96.3	99.1	98.9	99.2
No. Outlier Residues	33	8	10	7

Table S1. Summary data from Ramachandran plots of the homology model, and of Med1, Med2 and Med3.

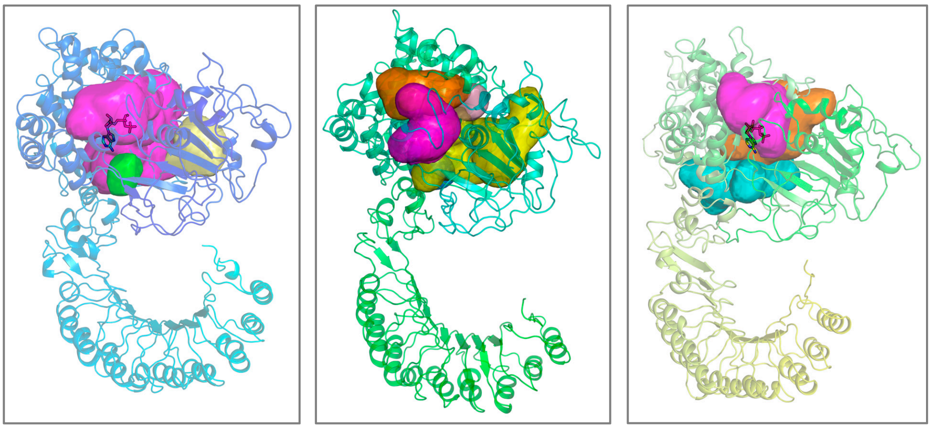

(**a**) (**b**) (**c**)

Figure S8. ADP pocket in medoids extracted from MD simulation. ADP pocket (magenta) in (**a**) Med1, (**b**) Med2, (**c**) Med3.

Centroid	Radius (Å)
A228	8
R351	10
Q526	10
N506	10
K238	10
M647	10
V223	10
L634	10

Table S2. Centroids and radii used for docking studies in the ADP pocket. These sites were investigated in Med1, Med2 and Med3.

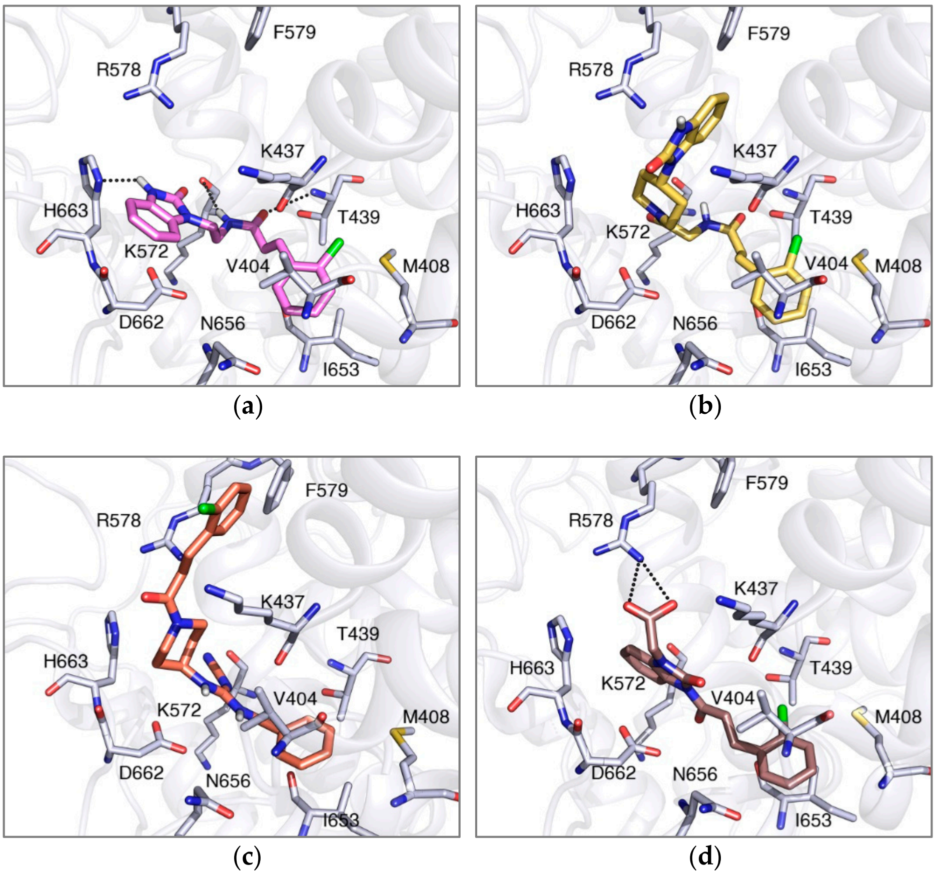

Figure S9. Docking of derivatives 9, 6, 13, 18 in pocket p16 of Med1. Med1 protein is represented as light blue cartoon, site residues and ligands are shown as sticks. (**a**) **9** (pink), (**b**) **6** (yellow), (**c**) **13** (orange), (**d**) **18** (brown).

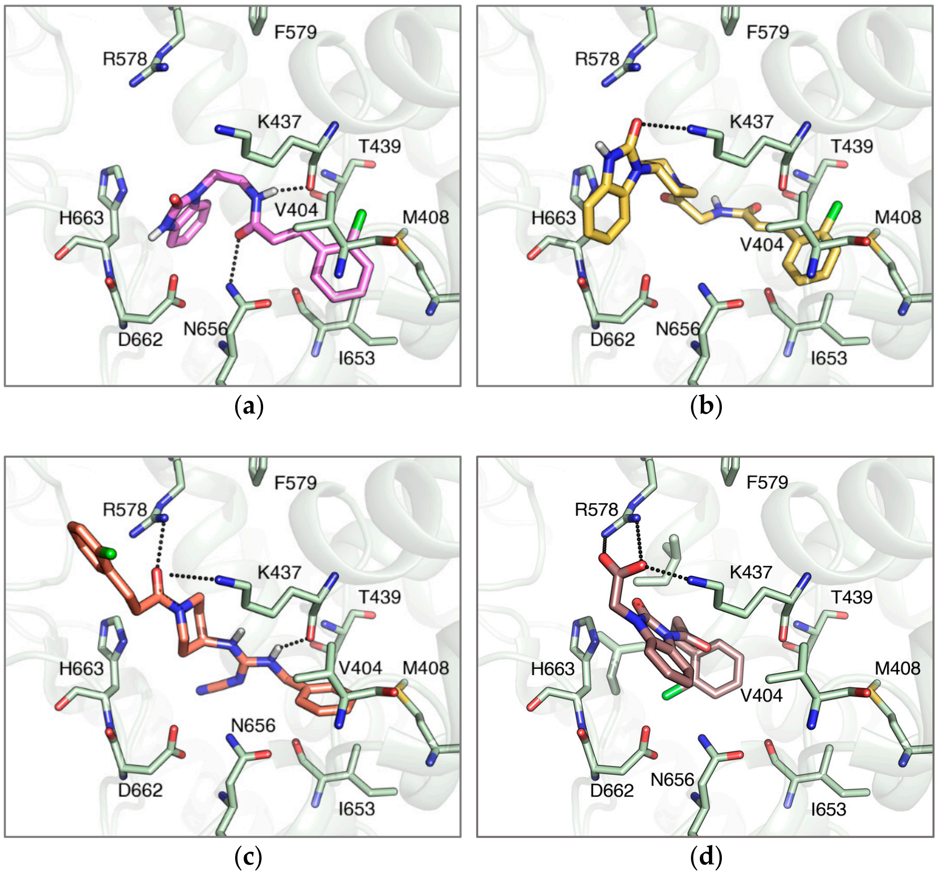

(**a**) (**b**)

(**c**) (**d**)

Figure S10. Docking of derivatives 9, 6, 13, 18 in pocket p16 of Med2. Med2 protein is represented as light green cartoon, site residues and ligands are shown as sticks. (**a**) **9** (pink), (**b**) **6** (yellow), (**c**) **13** (orange), (**d**) **18** (brown).

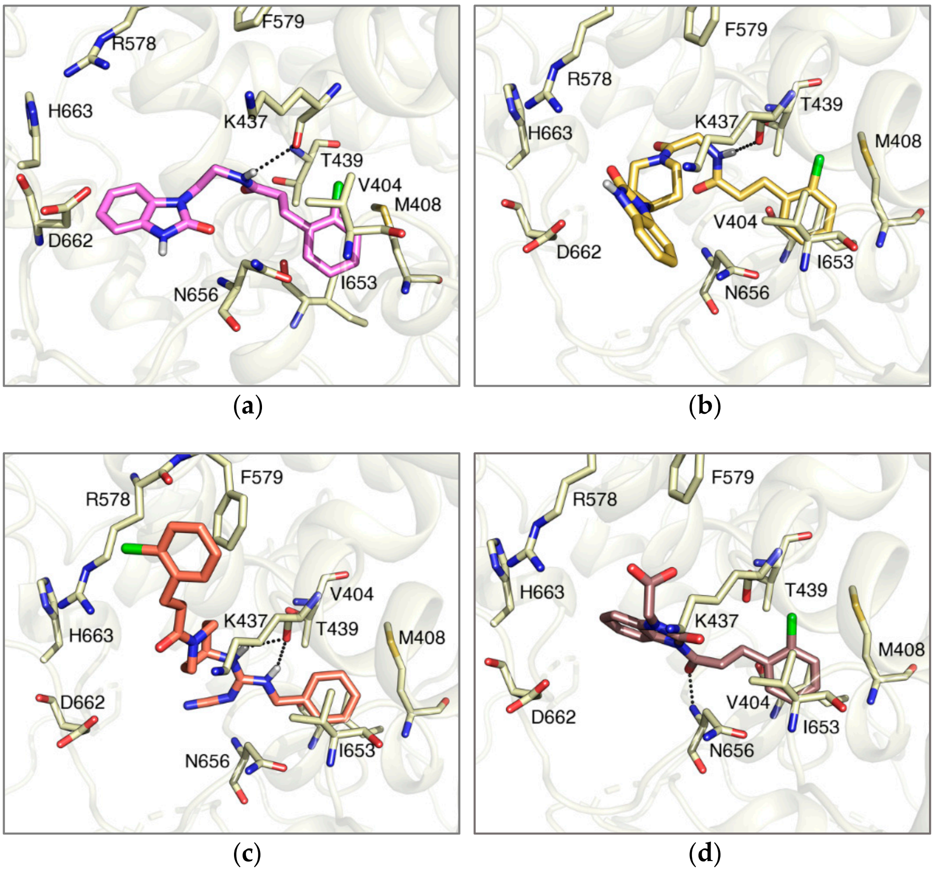

Figure S11. Docking of derivatives 9, 6, 13, 18 in pocket p16 of Med3. Med3 protein is represented as light yellow cartoon, site residues and ligands are shown as sticks. (**a**) **9** (pink), (**b**) **6** (yellow), (**c**) **13** (orange), (**d**) **18** (brown).

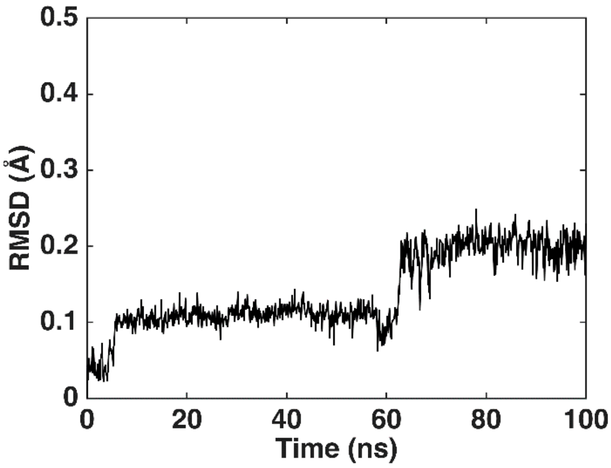

Figure S12. RMSD of compound 9 (INF120) along 100 ns MD simulation.
